# Prevalence and representation of comorbidities and multimorbidity in randomised controlled trials in sepsis or septic shock: a systematic review

**DOI:** 10.1186/s13054-026-06049-y

**Published:** 2026-05-06

**Authors:** Sinziana Maria Radulescu, Stella Prizeman-Green, Sohan Seth, Nazir I. Lone, Annemarie B. Docherty

**Affiliations:** 1 Centre for Medical Informatics, Usher Institute, University of Edinburgh, Edinburgh, UK; 2 School of Informatics, University of Edinburgh, Edinburgh, UK; 3 Centre for Population Sciences, Usher Institute, University of Edinburgh, Edinburgh, UK

**Keywords:** Sepsis, Critical care, Randomised controlled trials, Multimorbidity, Comorbidity

## Abstract

**Background:**

Multimorbidity is prevalent among critically ill patients with sepsis yet remains underreported in critical care randomised controlled trials (RCTs). Inadequate reporting limits the generalisability of findings and the ability to understand whether chronic disease burden modifies treatment effects. We aimed to systematically map and evaluate how comorbidities and multimorbidity are represented, reported, and analysed in RCTs involving Intensive Care (ICU) patients with sepsis or septic shock.

**Methods:**

We searched MEDLINE, Embase, CENTRAL, LILACS, Web of Science, medRxiv and clinical trial registries for RCTs between January 1992 and August 2025 for trials enrolling adult ICU patients with sepsis or septic shock. Two reviewers independently screened studies and extracted data using a predefined protocol registered on PROSPERO (CRD42024510506). Trials were categorised according to whether baseline comorbidity information was reported. Reported comorbidities were mapped to a Delphi-derived classification framework.

**Results:**

From 15,830 records, 591 RCTs met inclusion criteria. Of these, 209 trials (35.4%) reported baseline comorbidity data, enrolling a total of 48,429 patients (median 91 per trial). Trials reporting comorbidity information differed systematically from those that did not: they were larger, more likely to have publicly available protocols, more frequently used mortality as a primary outcome, and more often demonstrated low risk of bias in several methodological domains. Reporting of comorbidities increased over time (ρ = 0.80, p < 0.001), with the odds of reporting baseline comorbidity data increasing by approximately 8% per year (OR 1.08, 95% CI 1.06–1.11). Among trials reporting comorbidity data, the most frequently reported conditions were diabetes (86.1% of trials), hypertension (65.1%), chronic kidney disease (56%), and cancer (53.1%). Despite the high prevalence of comorbidities, only four trials (1.9%) explicitly reported multimorbidity and just 12 trials (5.7%) used a structured framework such as the Charlson Comorbidity Index. Nearly 80% of trials excluded participants based on at least one comorbidity.

**Conclusions:**

Baseline comorbidity data are absent from most sepsis RCTs, and trials that report such information differ systematically from those that do not. Standardised frameworks and transparent reporting of comorbidities and multimorbidity are needed to improve representativeness, enable subgroup analyses, and enhance the external validity of sepsis research.

**Supplementary Information:**

The online version contains supplementary material available at 10.1186/s13054-026-06049-y.

## Introduction

Sepsis is defined as a life-threatening organ dysfunction caused by a dysregulated host response to an infection [1], and remains a leading cause of critical illness [2]. Despite advances in early recognition, standardised resuscitation bundles, and antimicrobial therapy, sepsis continues to carry high mortality and poor long-term outcomes [3]. Numerous critical care interventions for sepsis, despite being theoretically sound, have not demonstrated a positive impact in randomised controlled trials (RCTs) [3–5]. Recent progress in omics technologies, big data analytics and machine learning has underscored the marked heterogeneity of sepsis as a syndrome [5] - heterogeneity that may be driven in part by the wide spectrum of comorbidity and multimorbidity profiles encountered in the ICU [6, 7]. Despite this population variability, trials often underrepresent real-world ICU populations in terms of multimorbidity [8], socioeconomic status [9, 10] and ethnicity [11].

Multimorbidity, defined as the coexistence of two or more long-term conditions [12], is rising in prevalence worldwide [13] and poses an increasingly complex challenge for critical care [6]. In the UK, more than 60% of the critical care population has two or more comorbidities [14]. Beyond its impact on prognosis, multimorbidity has important implications for how patients respond to treatment [15]. Evidence from individual participant data meta-analyses across multiple therapeutic areas has shown that comorbid conditions can modify both treatment efficacy and the risk of adverse events, contributing to heterogeneity of treatment effects (HTEs) [16–18]. Despite this, multimorbidity (and individual comorbidities) remain underrepresented and underreported in clinical trials, which limits not only generalisability, but the capacity to assess whether the burden of chronic disease modifies HTEs and risk of adverse events [8, 19]. These effects may be particularly relevant in critical care [20], where patients frequently receive complex interventions with narrow therapeutic margins. Differences in comorbidity burden may therefore influence both the benefits and harms of treatment and may partly explain why many promising sepsis interventions have failed to demonstrate consistent benefit in RCTs.

However, evaluation of HTEs requires that comorbidities are systematically measured and reported in trial populations. Without consistent reporting of baseline comorbidity information, it is difficult to determine whether trial populations are representative of real-world ICU populations, and impossible to examine whether treatment effects differ across comorbidity strata [21]. While prior reviews have examined subgroup effects of age [22, 23], multimorbidity and frailty-related vulnerability [23] in older critically ill populations, none have systematically characterised how comorbidities are defined, reported and operationalised across ICU trials.

In this systematic review, we aimed to investigate the prevalence of multimorbidity and individual comorbid conditions within adult patient populations with sepsis and/or septic shock in ICUs across existing RCTs. Specifically, we sought to (1) quantify how frequently baseline comorbidity information is reported in sepsis trials, (2) characterise the types of comorbidities reported and the frameworks used to describe them, and (3) examine how comorbidity reporting practices have changed over time. By mapping how comorbidities are currently reported in sepsis RCTs, we aimed to identify gaps that may limit the ability of trials to evaluate treatment effect heterogeneity and to inform future reporting standards in critical care research.

## **Methods**

The study protocol was registered on PROSPERO (CRD42024510506). This study followed the Preferred Reporting Items for Systematic Reviews and Meta-analyses (PRISMA) reporting guidelines (Additional file 1).

### Search strategy

We conducted a systematic search of the literature using public-domain databases including MEDLINE, Embase, Cochrane Central Register of Controlled Trials (CENTRAL), LILACS, medRxiv, Web of Science as well as the clinical trials registries and conference proceedings. We aimed to identify all RCTs published from January 1992 until August 2025 that had evaluated any intervention in adult critically ill ICU patients with sepsis and/or septic shock. We chose 1992 as the starting point for our search, as this marks the publication of the first formal definitions of sepsis, which emerged from the consensus statement meeting held in August 1991 [24]. We stratified the results into three periods: 1992–2000, 2001–2015 and 2016–2025. This stratification was based on the first [24], second [25] and third [8] international sepsis definitions conferences.

For the search, medical subject heading (MeSH) terms and text words were used within the following three categories: (1) sepsis or septic shock 2) setting (Critical Care/Intensive care unit) and 3) randomised controlled trials. Additional search strategy information can be found in the Supplementary Material (Additional file 2).

## Selection criteria

We defined a priori the following criteria for study inclusion: (a) primary report of a RCT testing the efficacy or effectiveness of any intervention in patients with sepsis and/or septic shock (b) the study reports original data (protocols, posttrial follow-up studies, secondary or separate subgroup analyses were excluded); (c) the trial was published in English; (d) the RCT was conducted in ICU; (e) the RCT enrolled only adult subjects (> 18 years). A proportion of non-RCT studies were automatically removed using the Cochrane RCT classifier [26]. Two reviewers (SMR, SPG) independently screened both abstracts and the full text of studies identified through the literature search. All RCTs meeting these criteria were retained to characterise overall trial features. Trials were then categorised according to whether baseline comorbidity information was reported. Trials that reported at least one comorbidity (either as number or percentage of participants) were included in the comorbidity analysis. Trials that did not report any comorbidity data were retained for comparison of trial characteristics but were excluded from analyses requiring comorbidity information.

## Data extraction

Each included article was extracted by the first author using a standardised data extraction form that can be found in the Supplementary Material (Additional file 3). Data extraction was performed in two stages.

First, trial-level characteristics were extracted for all eligible RCTs with available study information. Information on region, year of publication, trial registration, sample size, funding, primary outcome assessed, intervention type and risk of bias were abstracted to describe overall characteristics of included studies. Risk of bias was assessed using the first Cochrane Risk of Bias Tool [27], evaluating the following domains: sequence generation, allocation concealment, blinding of participants and personnel, blinding of outcome assessment, incomplete outcome data and selective reporting.

Second, detailed extraction of comorbidity data was undertaken only for trials that reported at least one baseline comorbidity. Alongside comorbidity information, we extracted information on participant characteristics such as sex, age and ethnicity. For participant characteristics, including sex distribution, age, ethnicity, we calculated weighted summaries across all included trials, weighting each study by its sample size. To assess the reliability of data extraction and comorbidity mapping, a random 10% sample of included trials (21/209) was independently re-extracted by a second reviewer (SPG) blinded to the original extraction. Agreement was evaluated across key extracted variables, including comorbidity reporting, mapped comorbidity categories, intervention classification and exclusion-criteria coding. No material discrepancies were identified.

We evaluated whether trials reported the presence of comorbidities by reviewing the participant characteristics tables, included either in the main text or supplementary material. We further distinguished between a broad description of comorbidities in the study (e.g., cardiovascular disease) and a specific chronic condition (e.g., hypertension). We manually extracted the comorbidities reported and the number and/or percentage reported in either patients’ characteristics table and/or supplementary material for each study. Multimorbidity was defined as the presence of two or more pre-existing conditions [28] in a single trial participant. Multimorbidity was considered to be reported only when trial publications provided patient-level or structured comorbidity data permitting identification of individuals with ≥ 2 chronic conditions; trials that listed individual comorbidities without such a framework were not considered to report multimorbidity.

All reported comorbidities were extracted verbatim as presented in the original publications. An iterative consensus-based mapping framework was developed by three reviewers (SR, NL, AMD) to assign each reported term to a unique index comorbidity or, where reporting was imprecise or non-specific, to a broader body-system category. Each extracted term was mapped to a single category to avoid overlap. Where comorbidities were recorded as individual conditions, we mapped them to a total of 26 conditions adapted from a recent Delphi consensus study [28] (Additional file 4). Where comorbidities were not individually recorded but instead reported as a broad term, we mapped them to the body system (based on ICD-10 chapters) used in the same publication [24]. The prevalence of comorbidities was calculated as sample-size–weighted estimates, ensuring that trials contributed proportionally according to the number of enrolled participants. We assessed whether comorbidities were considered in any analyses of trial outcomes (subgroup analyses, comorbidity-adjusted analyses).

We reviewed inclusion and exclusion criteria and assessed the exclusion of individuals with chronic conditions. Chronic conditions stated in the exclusion criteria were summarised using word tokenisation, a natural language processing (NLP) technique [29]. Free-text exclusion criteria were tokenised and mapped using a predefined dictionary of comorbidity-related terms. During dictionary development, all extracted exclusion phrases were manually reviewed to ensure correct classification. Severity qualifiers (e.g. “severe”, “advanced”, “end-stage”) were retained in the extracted text but did not alter category assignment, which was based on the underlying comorbidity concept. Negated statements were not present in the analysed exclusion criteria, as only positive exclusion statements were included. Exclusion criteria that did not match any dictionary term were manually reviewed to confirm they did not represent comorbidity-related exclusions. Given the strong association of age and morbidity, we also evaluated whether trials used any age-related exclusion criteria (other than adults > = 18 years).

Descriptive statistics including mean, median, proportions and frequencies were used to summarize the study characteristics and measures as described above. Comparisons between trials reporting and not reporting comorbidity data were performed using Pearson’s χ² test for categorical variables and the Wilcoxon rank-sum test for sample size.

To assess temporal trends in the proportion of trials included based on comorbidity reporting, we used Spearman’s rank correlation coefficient (ρ) to evaluate the association between publication year and the percentage of included trials. To assess whether the proportion of trials reporting comorbidity data changed over time, we used a binomial generalised linear model (GLM).

Restricting the analysis to trials that reported any comorbidity data, the Kruskal-Wallis test was used to assess the difference between the number of comorbidities reported in different time periods. These analyses represent a post-hoc refinement of the original PROSPERO protocol, undertaken to better characterise temporal trends in reporting practices.

All analyses were performed using R (R statistical software Version 4.2.1, R Foundation for Statistical Computing, Vienna, Austria).

## Results

### Study selection

**Fig. 1:**
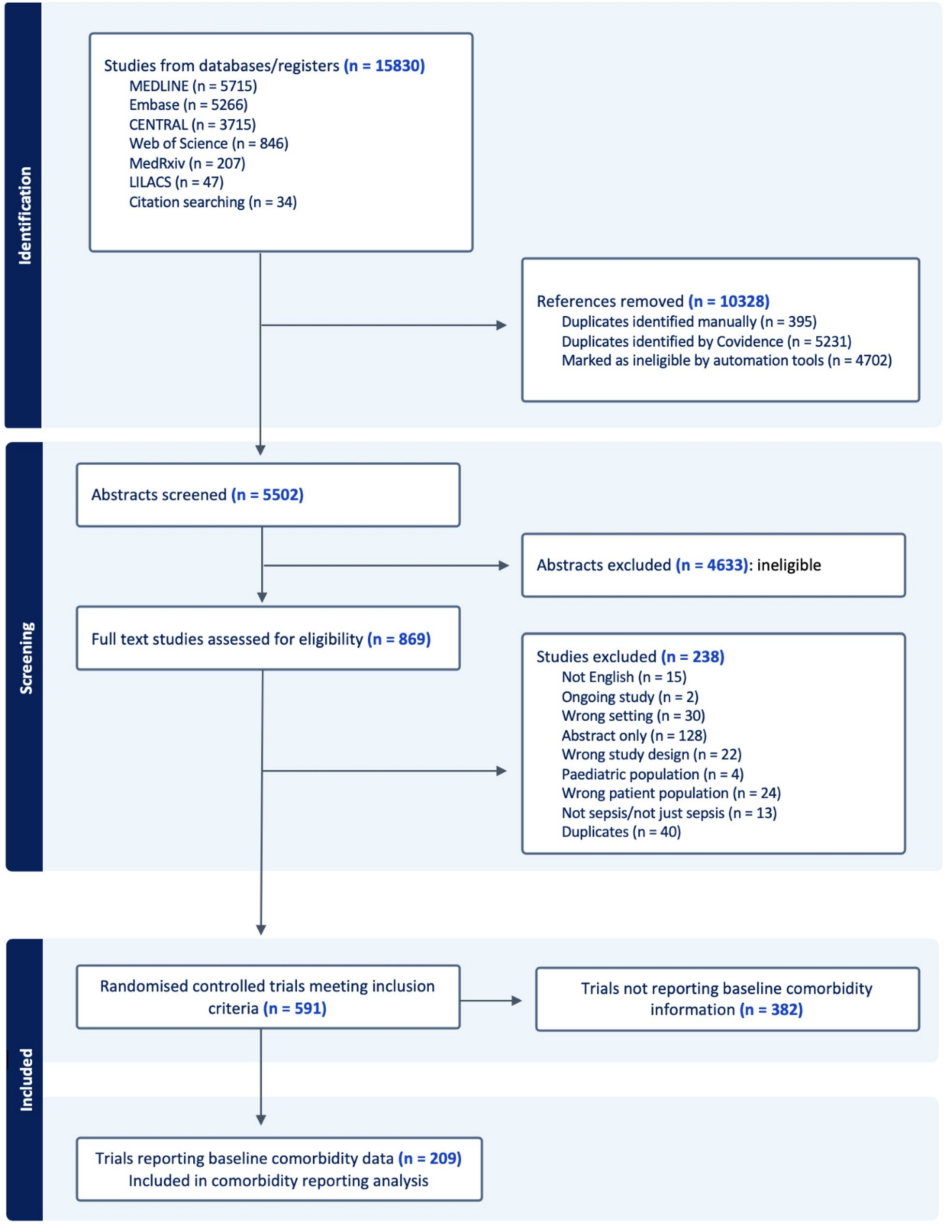
Flow diagram of trial selection showing 591 eligible trials, of which 209 reported comorbidity data

A total of 15,830 records were identified through the search (Fig. 1). After removing duplicate records and automatic non-RCT removal using the Cochrane RCT classifier [26], 5502 records remained. We first screened abstracts, then 869 full-text articles were assessed for eligibility. Out of 591 RCTs that met the inclusion criteria (a) to (e), only 209 (35.4%) reported information (number and/or percentage) on at least one comorbidity (Fig. 1). 382 (64.6%) trials were excluded from the comorbidity analysis as they did not report any comorbidity characteristic. A full list of the trials included, their characteristics and risk of bias is provided in the Additional file 5.

Trial characteristics differed between studies that reported baseline comorbidity data and those that did not (Table 1, Additional file 5). Among trials that reported comorbidity data, 46% were conducted in the Asia–Pacific region compared with 35% of trials that did not report comorbidities. In contrast, European trials accounted for 25% of trials reporting comorbidity data versus 39% of those that did not (*p* = 0.002). Protocol availability was substantially higher among trials reporting comorbidity data (76% vs. 43%, *p* < 0.001). Trials reporting comorbidities were also more likely to use mortality as the primary outcome compared with trials that did not report comorbidities (34% vs. 20%, *p* < 0.001).

**Table 1:** Characteristics of included trials according to reporting of comorbidity data Characteristics of 591 randomized controlled trials included in the systematic review, stratified by whether comorbidity data were reported.

Trial characteristic category	Trial characteristic variable	No comorbidity data (*N* = 382)	Reported comorbidity data (*N* = 209)	Total (*N* = 591)	*p*-value
Multicenter	Yes	148 (39%)	88 (42%)	236 (39.9%)	0.5
Region	Africa	24 (6.3%)	9 (4.3%)	33 (5.6%)	0.002
	Asia/Pacific	133 (35%)	96 (46%)	229 (39%)	
	Europe	150 (39%)	52 (25%)	202 (34%)	
	Latin America	12 (3.1%)	15 (7.2%)	27 (4.6%)	
	More than one	30 (7.9%)	15 (7.2%)	45 (7.6%)	
	North America	33 (8.6%)	22 (11%)	55 (9.3%)	
International	Yes	65 (17%)	24 (11%)	89 (15%)	0.093
Protocol available	Yes	163 (43%)	159 (76%)	322 (54%)	< 0.001
Trial registration	Yes - clinicaltrials.gov	100 (26%)	112 (54%)	212 (36%)	< 0.001
	Yes - other registry	68 (18%)	49 (23%)	117 (20%)	
	No	214 (56%)	48 (23%)	262 (44%)	
Funding	Industry	97 (25%)	42 (20%)	139 (24%)	0.037
	Non-industry	190 (50%)	127 (61%)	317 (54%)	
	Not reported	95 (25%)	40 (19%)	135 (23%)	
Primary outcome	Mortality	75 (20%)	72 (34%)	147 (25%)	< 0.001
	Other	307 (80%)	137 (66%)	444 (75%)	
Positive outcome	Yes	209 (55%)	97 (46%)	306 (52%)	0.065
Intervention category	Care bundles	10 (2.6%)	9 (4.3%)	19 (3.2%)	< 0.001
	Monitoring systems	15 (3.9%)	24 (11%)	39 (6.6%)	
	Therapeutic interventions	269 (70%)	122 (58%)	391 (66%)	
	Other strategies	88 (23%)	54 (26%)	142 (24%)	
Top-10 journal	Yes	173 (45%)	96 (46%)	269 (44%)	> 0.9
Number of participants	Median (IQR)	42 (20–100)	91 (56–211)	60 (29–134.5)	< 0.001
**Risk of bias**					
Sequence generation	High	4 (1.0%)	1 (0.5%)	5 (0.8%)	< 0.001
	Low	281 (74%)	182 (87%)	473 (78%)	
	Unsure	97 (25%)	26 (12%)	123 (21%)	
Allocation concealment	High	13 (3.4%)	3 (1.4%)	16 (2.7%)	< 0.001
	Low	180 (49%)	136 (65%)	316 (53%)	
	Unsure	189 (49%)	70 (33%)	259 (44%)	
Blinding of participants and personnel	High	204 (53%)	117 (56%)	321 (54%)	0.6
	Low	168 (44%)	89 (43%)	257 (43%)	
	Unsure	10 (2.6%)	3 (1.4%)	13 (2.2%)	
Blinding of outcome assessment	High	92 (24%)	27 (13%)	119 (20%)	< 0.001
	Low	237 (62%)	160 (77%)	397 (67%)	
	Unsure	54 (14%)	22 (11%)	73 (13%)	
Incomplete outcome data	High	47 (12%)	14 (6.7%)	61 (10%)	0.050
	Low	311 (81%)	186 (89%)	497 (84%)	
	Unsure	24 (6.2%)	9 (4.3%)	33 (5.6%)	
Selective reporting	High	23 (6%)	2 (1.0%)	25 (4.2%)	< 0.001
	Low	177 (46%)	158 (76%)	335 (57%)	
	Unsure	182 (48%)	49 (23%)	231 (39%)	

Values are presented as number of trials (percentage within each group). Comparisons between trials reporting and not reporting comorbidity data were performed using Pearson’s χ² test for categorical data and the Wilcoxon rank-sum test for sample size. Risk-of-bias domains correspond to the Cochrane risk-of-bias tool and include sequence generation, allocation concealment, blinding of participants and personnel, blinding of outcome assessment, incomplete outcome data, and selective reporting

Funding source differed modestly between groups (*p* = 0.037), with non-industry funding more frequent among trials reporting comorbidity data (61% vs. 50%). Intervention categories also varied between groups (*p* < 0.001), with therapeutic interventions more common among trials that did not report comorbidity data (70% vs. 58%).

There was no significant difference in the proportion of multicentre trials (39% vs. 42%, *p* = 0.6), international trials (11% vs. 17%, *p* = 0.093), or trials published in top-10 journals (46% vs. 45%, *p* > 0.9). Similarly, the proportion of trials reporting positive outcomes did not differ significantly between groups (46% vs. 55%, *p* = 0.065). Trials reporting comorbidity data had larger sample sizes compared with those that did not (median 91 vs. 42 participants, *p* < 0.001) (Table 1).

Risk-of-bias assessments showed that trials reporting comorbidity data more frequently demonstrated low risk of bias for sequence generation (87% vs. 74%), allocation concealment (65% vs. 49%), blinding of outcome assessment (77% vs. 62%), and selective reporting (76% vs. 46%) (all *p* < 0.001). No significant difference was observed for blinding of participants and personnel (*p* = 0.6) or incomplete outcome data (*p* = 0.05).

**Fig. 2:**
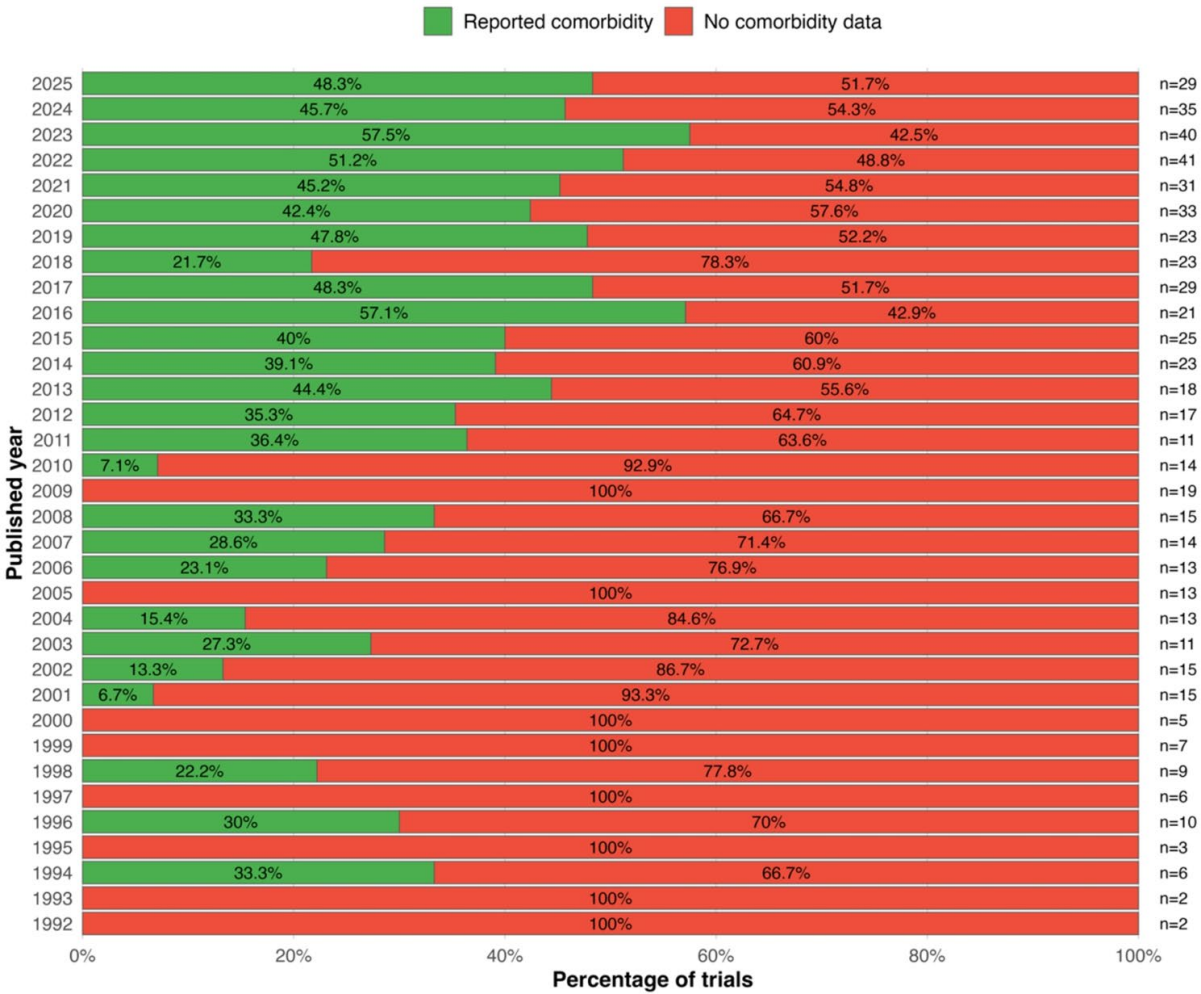
The percentage of included trials (*n* = 209, green) for reporting any comorbidity information out of all the trials (*n* = 591) that met all the other inclusion criteria (a) - (e). Red: trials excluded for lack of comorbidity reporting. A Spearman’s rank correlation showed a strong positive relationship between publication year and the percentage of trials included (ρ = 0.80, *p* < 0.001). The odds of reporting baseline comorbidity data increased significantly over time (OR 1.08 per year, 95% CI 1.06–1.11; *p* < 0.001)

The proportion of trials reporting comorbidities increased over time (Fig. 2). A Spearman’s rank correlation showed a strong positive relationship between publication year and the percentage of trials included (ρ = 0.80, *p* < 0.001). A binomial regression model showed a significant increase in the proportion of trials reporting comorbidities over time, with the odds of inclusion (by comorbidity reporting alone) rising by approximately 8% per year (OR 1.08, 95% CI 1.06–1.11; *p* < 0.001) ( Fig. 2).

### Study characteristics of trials reporting comorbidities

Out of the 209 trials, 157 (75.1%) were registered on a trial registry (e.g., clinicaltrials.gov). The majority of trials were from Asia or Pacific (48.8%), followed by Europe (24.4%) and North America (12%) (Additional file 6).

The median number of participants included across trials was 91 (IQR: 56–211). The total number of participants included in 209 trials was 48,429. The age of the participants was reported in all of the included studies, out of which 135 studies reported mean age (+/- standard deviation), 72 studies reported median age (+/- IQR), one study reported both mean and median, and one study reported only the range (Additional file 6). Out of the 209 trials, 37 (17.7%) applied additional age-related restrictions beyond the standard adult threshold ( > = 18 years): 30 trials imposed a maximum age limit between 60 and 90 years, while seven trials specified a minimum age requirement ranging from 20 to 65 years. Out of the studies that reported means and SD, the pooled mean age of participants was 61.2 (SD 15.9).

The sex of the participants was reported in 208/209 studies included, either as a number, percentage or both demonstrating a male preponderance in most studies (pooled 55% male patients (range 28.9–88.7%)). Ethnicity was reported in only 31 (14.8%) of the trials.

We identified a total of 153 unique comorbidities reported across the 209 included trials. Out of the 153 conditions reported, 46 did not map to any comorbidity. Inspection of these unmapped terms showed that most represented acute complications, treatments, laboratory abnormalities or manifestations of the index illness rather than chronic comorbid diseases. (Additional file 4). The remaining were mapped to 29 specific index conditions (e.g. heart failure) and, where comorbidities were reported as a broad term, we mapped them to 10 body-system categories (e.g. cardiovascular disease), resulting in a total of 39 mapped comorbidities (Table 2).

**Table 2:** Summary of comorbidity reporting across included trials (*N* = 209).

Comorbidity	Trials reporting: Number (%)	Number of patients (Prevalence - %)	Denominator
Diabetes	180 (86%)	8638 (24.3%)	35,492
Hypertension	136 (65.1%)	10,838 (39.1%)	27,752
Chronic kidney disease	117 (56%)	2534 (9%)	28,050
Cancer	111 (53.1%)	5797 (17.3%)	33,583
COPD	107 (51.2%)	3617 (13.5%)	26,782
Chronic liver disease	94 (45%)	1721 (5.9%)	29,132
Heart failure	87 (41.6%)	3138 (11.5%)	27,231
Coronary artery disease	91 (43.5%)	2961 (13.5%)	21,966
Cardiovascular disease	45 (21.5%)	2209 (17.9%)	12,321
Respiratory disease	42 (20.1%)	1977 (16.1%)	12,293
Stroke	29 (13.7%)	776 (7.4%)	10,493
Drug or alcohol misuse	21 (10%)	1032 (18.5%)	5584
Neurological disease	12 (5.7%)	476 (17.1%)	2789
Arrhythmia	11 (5.3%)	355 (14.5%)	2445
Peripheral arterial disease	8 (3.8%)	51 (4.8%)	1071
Thyroid disorders	7 (3.3%)	71 (6.1%)	1155
Chronic pancreatitis	7 (3.3%)	244 (4.4%)	5548
Asthma	5 (2.4%)	40 (14.2%)	282
Venous thromboembolic disease	5 (2.4%)	137 (3.2%)	4270
Dementia	5 (2.4%)	75 (6.4%)	1175
Digestive disease	5 (2.4%)	128 (10.5%)	1218
Haematological disorder	4 (1.9%)	46 (4.6%)	1006
HIV	4 (1.9%)	16 (3.2%)	499
Epilepsy	3 (1.4%)	16 (5.9%)	269
Osteoporosis	2 (1%)	5 (1.2%)	420
Connective tissue disease	2 (1%)	7 (5%)	140
Metabolic and endocrine disease	2 (1%)	50 (48.1%)	104
Urogenital disorder^†^	1 (0.5%)	20 (8.8%)	228
Inflammatory bowel disease^†^	1 (0.5%)	4 (2.7%)	150
Tuberculosis^†^	1 (0.5%)	8 (6.6%)	122
Anaemia^†^	1 (0.5%)	318 (45.4%)	701
Congenital disease and chromosomal abnormalities^†^	1 (0.5%)	1 (0.7%)	150
Osteoarthritis^†^	1 (0.5%)	5 (4.9%)	103
Infectious diseases^†^	1 (0.5%)	6 (9%)	67
Mental and behavioural disorder^†^	1 (0.5%)	1 (1.7%)	60
Schizophrenia^†^	1 (0.5%)	1 (6.2%)	16
Heart valve disorders^†^	1 (0.5%)	18 (36%)	50
Gout^†^	1 (0.5%)	1 (4.2%)	24

† Prevalence estimates based on a single contributing trial

For each row, we report the number of trials reporting each condition; prevalence estimates are calculated using only participants from trials that reported the given comorbidity. The denominator column reports this condition-specific participant subset

The number of comorbidities reported in the included trials ranged from 2 to 21 with a median number of 6 comorbidities (IQR: 3–9) (Figure S7, Additional file 7). The median (IQR) number of comorbidities reported was 8 (5.5–8.5) in 1992–2000, 7 (4–8) in 2001–2015, and 6 (4–7) in 2016–2025. The number of comorbidities reported per trial differed significantly across time periods (Kruskal–Wallis χ²(2) = 6.25, *p* = 0.040) (Table S7, Additional file 7).

**Fig. 3:**
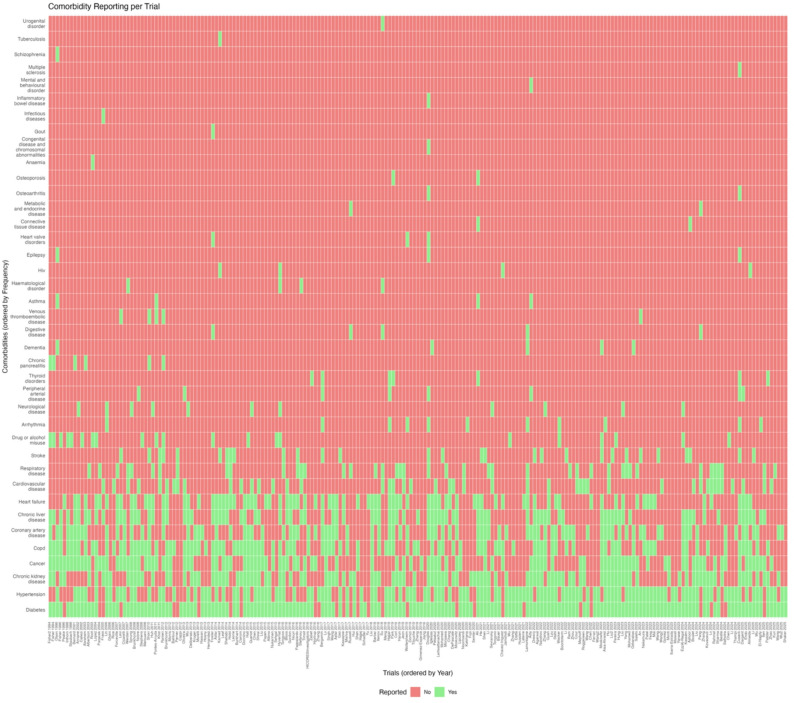
Heatmap displaying the reporting of specific and general comorbidities across all included trials. Comorbidities are ordered by decreasing frequency of reporting across trials and trials are ordered by publication year

The variation in reporting comorbidities across all included trials is displayed in Fig. 3. The most frequently reported comorbidities across trials were diabetes, reported in 180/209 (86.1%) of trials, hypertension (65.1% of trials), chronic kidney disease (CKD) (56%) and cancer (53.1%) (Table 2). The frequency of comorbidities reported in included trials stratified by time period is included in Additional file 7.

The prevalence of reported comorbidities varied across trials. Hypertension was reported in 39.1% of participants across 136 trials, diabetes in 24.3% (180 trials), cardiovascular disease in 17.9% (45 trials) and cancer in 17.3% (111 trials). In contrast, conditions such as metabolic and endocrine disease (48.1%, two trials), anaemia (45.4%, one trial), and heart valve disorders (36%, one trial) were reported in a smaller number of trials (Table 2).

Only four studies (1.9%) [30–33] reported any information related specifically to multimorbidity. Of these, three trials [31–33] enrolled relatively small cohorts, ranging between 16 and 29 participants, and reported comorbidity data (and therefore implied multimorbidity) at the individual patient level (Additional file 8).

A specific comorbidity framework or count-based definition was used in 12/209 (5.7%) trials [34–45]. Out of these, the Charlson Comorbidity Index (CCI) [46] score was used in 10 trials [34–38, 41–45] whilst the chronic health APACHE II score points [47] were reported in two trials [38, 39] (Additional file 9). A full breakdown of the list of the conditions included in these scores was not provided. Only five studies performed subgroup analysis accounting for comorbidities [30, 48–51]. In one of these five trials, only one comorbidity – CKD – was considered in the analysis [49] (Additional file 10).

The source of data used to determine comorbidities was reported in only 3 out of 209 trials (1.4%) [52–54]. In all three cases, the trials referenced previous hospital admissions for the reported comorbidities, suggesting that hospital records were used to derive them.

Upon evaluating the exclusion criteria for each trial included in the analysis, 163/209 (78%) of the trials excluded participants based on the presence of at least one comorbidity category. To better understand exclusion criteria related to chronic conditions, we applied a tokenisation approach to the free-text exclusion criteria across all trials. This process identified 1,137 unique tokens. From these, we isolated 82 terms that referred to pre-existing comorbidities. These comorbidity-related terms were often too specific to map to our existing index comorbidities (e.g. severe preexisting parenchymal liver disease with clinically significant portal hypertension). We therefore categorised them into the same body system categories used when mapping reporting comorbidities (e.g. digestive disease) with the addition of a new category for disorders of the immune system. Among the 209 trials, the most frequently cited comorbidities used as exclusion criteria were cardiovascular disease (reported in 70 trials, 33.5%), urogenital disorders (66 trials, 31.6%), digestive diseases (58 trials, 27.8%), and disorders of the immune system (56 trials, 26.8%) (Additional file 11).

## Discussion

In this study, we found that just over one-third of sepsis ICU RCTs reported comorbidity data and multimorbidity can rarely be inferred. Although reporting increased over time, more recent trials that do report comorbidities tend to enumerate fewer distinct conditions. Our comparative analysis of all 591 eligible trials identified several differences in broader characteristics of trial design and reporting quality. Trials reporting baseline comorbidity data tended to be larger, were more likely to have publicly available protocols, more frequently used mortality as a primary outcome, and demonstrated lower risk of bias across several methodological domains. We also observed regional variation, with trials conducted in the Asia–Pacific region more likely to report comorbidity information than those conducted in Europe. In contrast, the likelihood of comorbidity reporting did not differ substantially between multicentre and single-centre studies or between international and single-country trials, suggesting that broader reporting practices rather than trial scale alone may drive these differences. A modest difference in funding source was also detected, with non-industry funding more common among trials reporting comorbidity data, which may reflect differences in reporting practices or transparency requirements across funding structures.

Despite these findings, the mechanisms underlying under-reporting of comorbidities in sepsis RCTs remain uncertain. It is possible, however, that greater transparency in trial design may be associated with more complete reporting of baseline patient characteristics. When protocols are unavailable, secondary users cannot determine whether comorbidity data were collected but not reported, or whether they were never captured at all, further reinforcing the indistinguishability of these scenarios and limiting the ability to assess trial representativeness or explore heterogeneity of treatment effects. From the perspective of secondary users of the literature, trials that did not collect comorbidity data, trials that collected but did not report such data, and trials with unstructured or poor-quality comorbidity ascertainment are functionally indistinguishable, as none permit identification or analysis of multimorbidity. This is likely to affect clinical decision-making for patients with multiple conditions and can limit generalisability through evidence of HTE [5]. A plausible contributor is also the absence of explicit, standardised reporting requirements for comorbidities. Although CONSORT recommends reporting baseline demographic and clinical characteristics [55] and identifies comorbid conditions as key determinants of external validity [56], it does not explicitly mandate structured comorbidity or multimorbidity reporting. Moreover, operational definitions of reported comorbidities are rarely provided, limiting the ability to interpret how these conditions were ascertained or defined within individual trials. In most studies it is unclear whether comorbidities were identified using standard diagnostic criteria, administrative coding, medical history, or other sources, and whether distinctions were made between active disease, historical diagnoses, or disease severity.

In our review, although the age of participants was reported in all trials that reported comorbidities, we found that 30 trials (14.4%) applied upper age exclusion criteria. Despite advanced age and multimorbidity being closely intertwined in the ICU population [3] and increasing in prevalence in the ICU population [57], many ICU trials apply age-related restrictions [22]. Ethnicity was reported in just 14.8% of trials included in comorbidity analysis and remained consistently underreported over time. Sex was universally reported, with male participants comprising 55% of the study population, consistent with prior evidence showing a male predominance of ICU populations [58].

The prevalence of reported comorbidities varied widely and indicate that the reported prevalence of comorbidities in sepsis trials may not reflect the true burden observed in broader ICU populations. For instance, the trials prevalences of commonly reported comorbidities such as hypertension (39.1%) and diabetes (24.3%) were significantly lower, compared to 52.5% and 36.7% in a recent large-scale population study of critically ill patients [13]. These discrepancies may reflect selective enrolment strategies, choice of comorbidities to report, genuine differences between trial and real-world populations, or under-reporting within trial publications, which are not mutually exclusive. Moreover, almost 80% of RCTs included in our review explicitly excluded individuals with at least one chronic condition, which will affect the reported prevalence.

In our review, only 4/209 trials [30–33] reported any information related specifically to multimorbidity and 5/209 studies [30, 48–51] performed subgroup analysis accounting for comorbidities. Only in the study by Tongyoo et al. [30] multimorbidity was addressed through a subgroup analysis based on patients having three or more comorbidities. This limited consideration of multimorbidity aligns with findings from a recent systematic review examining effect modification by age, frailty and multimorbidity in ICU trials, which identified only two studies reporting multimorbidity subgroup data and none in sepsis populations [23].

Numerous tools have been developed and utilized to assess multimorbidity, including simple (unweighted) disease counts, weighted disease indices, and medication-based measures [59]. However, only 5.7% of sepsis RCTs employed a defined comorbidity reporting tool, most commonly the Charlson Comorbidity Index (CCI) [46], reported in 10 (4.7%) trials. None of these studies reported the full 19 conditions included in the Charlson comorbidity framework [60]. Complete reporting of the component conditions, their operational definitions, and the source of ascertainment would enhance the utility of the Charlson framework and improve reproducibility and harmonisation across trials. Although the APACHE II score [47]—commonly used in ICU trials—was frequently reported to assess disease severity, the chronic health component of the score was not separately described or analysed in any of the trials, limiting the potential use of APACHE II to capture comorbidity burden in a systematic way specific to the ICU population.

Despite the availability of various tools, implementing consistent comorbidity and multimorbidity reporting in research remains challenging. A recent Delphi consensus study proposed a core list of 24 conditions that should always be included, and 35 that should usually be included, when measuring multimorbidity [28]. In our study, however, among the 153 unique comorbidities reported across trials, only 106 could be mapped to those identified in the Delphi consensus. This discrepancy may reflect the fact that most do not reflect chronic pre-existing diseases but instead comprise acute complications (e.g. acute kidney injury), medications, laboratory abnormalities, risk factors, and conditions intrinsically related to the index septic episode. This highlights heterogeneous and conceptually inconsistent use of “comorbidity” within trial reporting, which limits the ability of existing multimorbidity frameworks to be applied. Alternatively, it may indicate that the conditions prioritised in the Delphi consensus are not fully aligned with those most relevant to the ICU trial population.

The source of data used to ascertain comorbidities was reported in only three of 209 trials (1.4%), indicating limited transparency regarding how comorbidity information was collected and defined. In all three trials, comorbidities were derived from previous hospital admissions. While hospital records can provide structured and clinically validated information [61], the prevalence of multimorbidity and individual comorbidities varies substantially according to data source [62], and reliance on hospital data may omit less severe conditions that have not required or been recorded during an admission. Consequently, in the vast majority of sepsis RCTs it is not possible to determine whether comorbidity data were derived from hospital records, patient self-report, administrative databases, or other sources, nor whether ascertainment was systematic or selective. From the perspective of clinicians and meta-analysts, this absence has enormous implications for the interpretability, reproducibility, assessment of external validity, and the ability to explore treatment effect heterogeneity across clinically relevant comorbidity and multimorbidity strata.

To our knowledge, this is the first systematic review to focus specifically on how comorbidities are reported in sepsis trials. The strengths of our review mainly derive from solid methodological rigour: we searched seven bibliographic databases along with trial registries and conference abstracts covering 1992–2025 under a prospectively registered PROSPERO protocol. We screened more than 15,830 records in duplicate and extracted detailed comorbidity from 209 trials enrolling over 48,000 participants with sepsis and/or septic shock. By mapping the reported conditions to a condition list derived from a consensus framework [28], we enabled consistent cross-trial comparison. The time-trend analyses showed that reporting and eligibility practices have evolved, but overall, we were able to show that comorbidity reporting remains poor and inconsistent in sepsis trials in ICU.

This review has several limitations. Reliance on published summaries—without contacting trialists—means any under-reporting in primary articles propagates directly into the synthesis. The review is limited to English-language, adult studies. Moreover, fewer than 2% of studies described how comorbidity status was ascertained and only four trials explicitly defined or analysed multimorbidity, so quantitative links between comorbidity burden and treatment effects remain unexplored. The analytical outputs of this review primarily describe how comorbidities and multimorbidity are captured, reported and operationalised across sepsis randomised controlled trials, rather than synthesising intervention effects. This reflects the limited availability and marked heterogeneity of structured comorbidity data within the trial literature, which constrained quantitative synthesis. Lastly, although we mapped reported comorbidities to a Delphi-derived consensus framework [28], this framework was not originally developed specifically for the ICU context. While the majority of unmapped terms reflected heterogeneous or conceptually inappropriate use of the term “comorbidity”, this finding may also indicate limitations in the applicability of existing frameworks to critically ill populations.

In conclusion, this systematic review shows that comorbidities in ICU RCTs are still poorly reported, multimorbidity remains almost invisible, and eligibility criteria commonly exclude patients with chronic illness—together limiting the external validity of current evidence. Although reporting has improved over the past three decades, a paradigm shift is needed: future trials should adopt a comorbidity framework relevant to the ICU context and document their data sources. It is essential to be able to test for modification of treatment effects in sepsis interventions to ensure that trial findings are applicable to the increasingly multimorbid ICU population.

## Supplementary Information

Below is the link to the electronic supplementary material.

Supplementary Material 1.

Supplementary Material 2.

## Data Availability

The datasets used and/or analysed during the current study are available from the corresponding author upon reasonable request.
