# Supplementary material for: Prevalence and representation of comorbidities and multimorbidity in randomised controlled trials in sepsis or septic shock: a systematic review
Source: Crit Care. 2026 May 6;30:339. doi: 10.1186/s13054-026-06049-y (PMC13312557; doi:10.1186/s13054-026-06049-y)
Supplement: Supplementary file 1 — Supplementary Material 1. [file 13054_2026_6049_MOESM1_ESM.docx]

## **Additional file 1: PRISMA Checklist**

| **Section and Topic** | **Item #** | **Checklist item** | **Location where item is reported** |
| --- | --- | --- | --- |
| **TITLE** | | |  |
| Title | 1 | Identify the report as a systematic review. | Page 1 |
| **ABSTRACT** | | |  |
| Abstract | 2 | See the PRISMA 2020 for Abstracts checklist. | Page 2-4 |
| **INTRODUCTION** | | |  |
| Rationale | 3 | Describe the rationale for the review in the context of existing knowledge. | Page 4-5 |
| Objectives | 4 | Provide an explicit statement of the objective(s) or question(s) the review addresses. | Page 5-6 |
| **METHODS** | | |  |
| Eligibility criteria | 5 | Specify the inclusion and exclusion criteria for the review and how studies were grouped for the syntheses. | Page 7 |
| Information sources | 6 | Specify all databases, registers, websites, organisations, reference lists and other sources searched or consulted to identify studies. Specify the date when each source was last searched or consulted. | Page 6 |
| Search strategy | 7 | Present the full search strategies for all databases, registers and websites, including any filters and limits used. | Additional file 2 |
| Selection process | 8 | Specify the methods used to decide whether a study met the inclusion criteria of the review, including how many reviewers screened each record and each report retrieved, whether they worked independently, and if applicable, details of automation tools used in the process. | Page 7 |
| Data collection process | 9 | Specify the methods used to collect data from reports, including how many reviewers collected data from each report, whether they worked independently, any processes for obtaining or confirming data from study investigators, and if applicable, details of automation tools used in the process. | Page 8  Additional file 3 |
| Data items | 10a | List and define all outcomes for which data were sought. Specify whether all results that were compatible with each outcome domain in each study were sought (e.g. for all measures, time points, analyses), and if not, the methods used to decide which results to collect. | Page 7 |
|  | 10b | List and define all other variables for which data were sought (e.g. participant and intervention characteristics, funding sources). Describe any assumptions made about any missing or unclear information. | Page 7-8  Additional file 3 |
| Study risk of bias assessment | 11 | Specify the methods used to assess risk of bias in the included studies, including details of the tool(s) used, how many reviewers assessed each study and whether they worked independently, and if applicable, details of automation tools used in the process. | Additional file 3 |
| Effect measures | 12 | Specify for each outcome the effect measure(s) (e.g. risk ratio, mean difference) used in the synthesis or presentation of results. | NA |
| Synthesis methods | 13a | Describe the processes used to decide which studies were eligible for each synthesis (e.g. tabulating the study intervention characteristics and comparing against the planned groups for each synthesis (item #5)). | NA |
|  | 13b | Describe any methods required to prepare the data for presentation or synthesis, such as handling of missing summary statistics, or data conversions. | Page 7-9 |
|  | 13c | Describe any methods used to tabulate or visually display results of individual studies and syntheses. | Page 7 |
|  | 13d | Describe any methods used to synthesize results and provide a rationale for the choice(s). If meta-analysis was performed, describe the model(s), method(s) to identify the presence and extent of statistical heterogeneity, and software package(s) used. | Page 7 |
|  | 13e | Describe any methods used to explore possible causes of heterogeneity among study results (e.g. subgroup analysis, meta-regression). | NA |
|  | 13f | Describe any sensitivity analyses conducted to assess robustness of the synthesized results. | NA |
| Reporting bias assessment | 14 | Describe any methods used to assess risk of bias due to missing results in a synthesis (arising from reporting biases). | Table 1 |
| Certainty assessment | 15 | Describe any methods used to assess certainty (or confidence) in the body of evidence for an outcome. | NA |
| **RESULTS** | | |  |
| Study selection | 16a | Describe the results of the search and selection process, from the number of records identified in the search to the number of studies included in the review, ideally using a flow diagram. | Page 8  Figure 1 |
|  | 16b | Cite studies that might appear to meet the inclusion criteria, but which were excluded, and explain why they were excluded. | NA |
| Study characteristics | 17 | Cite each included study and present its characteristics. | Table 1  Figure 3 |
| Risk of bias in studies | 18 | Present assessments of risk of bias for each included study. | NA |
| Results of individual studies | 19 | For all outcomes, present, for each study: (a) summary statistics for each group (where appropriate) and (b) an effect estimate and its precision (e.g. confidence/credible interval), ideally using structured tables or plots. | Page 8, 9, 10 |
| Results of syntheses | 20a | For each synthesis, briefly summarise the characteristics and risk of bias among contributing studies. | NA |
|  | 20b | Present results of all statistical syntheses conducted. If meta-analysis was done, present for each the summary estimate and its precision (e.g. confidence/credible interval) and measures of statistical heterogeneity. If comparing groups, describe the direction of the effect. | Page 8, 9 |
|  | 20c | Present results of all investigations of possible causes of heterogeneity among study results. | NA |
|  | 20d | Present results of all sensitivity analyses conducted to assess the robustness of the synthesized results. | NA |
| Reporting biases | 21 | Present assessments of risk of bias due to missing results (arising from reporting biases) for each synthesis assessed. | Table 1 |
| Certainty of evidence | 22 | Present assessments of certainty (or confidence) in the body of evidence for each outcome assessed. | NA |
| **DISCUSSION** | | |  |
| Discussion | 23a | Provide a general interpretation of the results in the context of other evidence. | Page 11-14 |
|  | 23b | Discuss any limitations of the evidence included in the review. | Page 14 |
|  | 23c | Discuss any limitations of the review processes used. | Page 14 |
|  | 23d | Discuss implications of the results for practice, policy, and future research. | Page 11-14 |
| **OTHER INFORMATION** | | |  |
| Registration and protocol | 24a | Provide registration information for the review, including register name and registration number, or state that the review was not registered. | Page 2, Page 5 |
|  | 24b | Indicate where the review protocol can be accessed, or state that a protocol was not prepared. | Page 2, Page 5 |
|  | 24c | Describe and explain any amendments to information provided at registration or in the protocol. | NA |
| Support | 25 | Describe sources of financial or non-financial support for the review, and the role of the funders or sponsors in the review. | COI forms |
| Competing interests | 26 | Declare any competing interests of review authors. | COI forms |
| Availability of data, code and other materials | 27 | Report which of the following are publicly available and where they can be found: template data collection forms; data extracted from included studies; data used for all analyses; analytic code; any other materials used in the review. | Table S2 |

*From:*  Page MJ, McKenzie JE, Bossuyt PM, Boutron I, Hoffmann TC, Mulrow CD, et al. The PRISMA 2020 statement: an updated guideline for reporting systematic reviews. BMJ 2021;372:n71. doi: 10.1136/bmj.n71

## **Additional file 2: Search strategy**

| **Step** | **Search Terms** |
| --- | --- |
| 1 | *sepsis/ or *shock, septic/ |
| 2 | (sepsis or septic or sepsis-induced or sepsis-associated).tw. |
| 3 | septic?emia.tw. |
| 4 | or/1-3 |
| 5 | Intensive Care Units/ |
| 6 | Critical Care/ |
| 7 | Critical Illness/ |
| 8 | (critical care or intensive care or ICU).mp. |
| 9 | 5or6or7or8 |
| 10 | 9 and 4 |
| 11 | exp animals/ not humans.sh. |
| 12 | 10 not 11 |
| 13 | Randomized controlled trials as Topic/ |
| 14 | Randomized controlled trial/ |
| 15 | Random allocation/ |
| 16 | Double blind method/ |
| 17 | Single blind method/ |
| 18 | Clinical trial/ |
| 19 | exp Clinical Trials as Topic/ |
| 20 | or/13-19 |
| 21 | (clinic$ adj trial$1).tw. |
| 22 | ((singl$ or doubl$ or treb$ or tripl$) adj (blind$3 or mask$3)).tw. |
| 23 | Placebos/ |
| 24 | Placebo$.tw. |
| 25 | Randomly allocated.tw. |
| 26 | (allocated adj2 random).tw. |
| 27 | or/21-26 |
| 28 | 20 or 27 |
| 29 | Case report.tw. |
| 30 | Letter/ |
| 31 | Historical article/ |
| 32 | Review of reported cases.pt. |
| 33 | Review, multicase.pt. |
| 34 | or/29-33 |
| 35 | 28 not 34 |
| 36 | 12 and 35 |
| 37 | 36 not ((exp infant/ or exp child/ or adolescent/) not exp adult/) |
| 38 | limit 37 to English language |
| 39 | limit 38 to yr=”1992-Current” |

## **Additional file** **3:** Data extraction tool

**Form 1: Basic information extraction tool (all trials)**

| **Record Number**  Number of assigned article |
| --- |
| **Journal**  Full name |
| **Publication Year**  YYYY format |
| **Country**  Country of first author affiliation |
| **Is this study an international trial?**  Yes  No |
| **Is this study a multicentre trial?**  Yes  No |
| **Is this study reported to be a registered clinical trial?**  Check all that apply  No Yes- clinicaltrials.gov  Yes- other registry |
| **Is the protocol available (previously published or trial registry)?**  Yes  No |
| **Sample Size**  Number of participants met inclusion criteria |
| **Is the primary outcome mortality or other?**  Mortality  Other |
| **Does the trial report a positive outcome (based on the primary outcome)?**  Yes  No |
| **What is the funding source of the trial?**  Industry  Non-industry  Not reported |

**Form 2: Risk of bias extraction tool (all trials)**

| **Sequence generation**  Describe the method used to generate the allocation sequence in sufficient detail to allow an assessment of whether it should produce comparable groups  High  Low  Unsure |
| --- |
| **Allocation concealment**  Describe the method used to conceal the allocation sequence in sufficient detail to determine whether intervention allocations could have been foreseen in advance of, or during, enrolment  High  Low  Unsure |
| **Blinding of participants and personnel**  Describe all measures used, if any, to blind study participants and personnel from knowledge of which intervention a participant received. Provide any information relating to whether the intended blinding was effective  High  Low  Unsure |
| **Blinding of outcome assessment**  Describe all measures used, if any, to blind outcome assessors from knowledge of which intervention a participant received. Provide any information relating to whether the intended blinding was effective  High  Low  Unsure |
| **Incomplete outcome data**  Describe the completeness of outcome data for each main outcome, including attrition and exclusions from the analysis. State whether attrition and exclusions were reported, the numbers in each intervention group (compared with total randomized participants), reasons for attrition/exclusions where reported, and any re-inclusions in analyses performed by the review authors.  High  Low  Unsure |
| **Selective reporting**  State how the possibility of selective outcome reporting was examined by the review authors, and what was found  High  Low  Unsure |

**Form 3: Comorbidity focused extraction tool (only trials that included comorbidity data)**

| **Number of experimental study arms**  Number of groups aside from the control group that receive the experimental intervention (usually 1-2) |
| --- |
| **Is the number of participants randomized to this group reported?** *Yes,* if authors report the number randomized |
| **Sample Size**  Number of participants randomised  Number of participants intervention arm  Number of participants control arm |
| **How is the sex of the participants in this sample reported?**  Check all that apply *Number* – number of participants randomized to this study arm *Percentage*- percentage of participants (out of study total) that were randomized to this study arm  *Not Reported*- no number or percentage of participants were reported for this study arm |
| **Number Male** Number of male participants in the intervention arm (reported or calculated). |
| **Number Male** Number of male participants in the control arm (reported or calculated). |
| **Number Female** Number of male participants in the intervention arm (reported or calculated). |
| **Number Female** Number of male participants in the control arm (reported or calculated). |
| **Percentage Male**  Percentage of male participants (out of study total) that were randomized to the intervention arm (reported or calculated). Number format from 0 to 100 |
| **Percentage Male**  Percentage of male participants (out of study total) that were randomized to the control arm (reported or calculated). Number format from 0 to 100 |
| **Percentage Female**  Percentage of male participants (out of study total) that were randomized to the intervention arm (reported or calculated). Number format from 0 to 100 |
| **Percentage Female**  Percentage of male participants (out of study total) that were randomized to the control arm (reported or calculated). Number format from 0 to 100 |
| **Sample Age Measures Reported**  Select all that apply  *Mean Range Standard Deviation*  *Median*  *IQR Not reported* |
| **Mean (Intervention & Control arms)**  Average age of study sample (reported or calculated).  **Range_Lower (Intervention & Control arms)** The age of the youngest participant enrolled in this study sample (reported or calculated).  **Range_Upper (Intervention & Control arms)**  The age of the oldest participant enrolled in this study sample (reported or calculated).  **Standard Deviation (Intervention & Control arms)**  **Median (Intervention & Control arms)** Median age of study sample reported |
| **Is the trial selection targeting individuals with one/more comorbidity/comorbidities?**  *No*, all participants have sepsis/septic shock with no chronic condition targeted  *Yes*, participants must have at least one chronic condition Ex: Each participant must have one of the following conditions: sepsis and diabetes, sepsis and cardiovascular disease etc. |
| **The study sample consists of individuals with which comorbidity/comorbidities?**  Copy and paste here each of the chronic condition(s) used for selecting patients in the trial |
| **Is eligibility criteria reported?**  *Yes* if any exclusion or inclusion criteria for participant eligibility is reported |
| **Did trial explicitly exclude individuals with any/multiple chronic conditions, regardless of conditions?**  *Yes* if individuals with one/more than one chronic condition (regardless of condition) were excluded from the trial |
| **Is the number of individuals excluded for having comorbid chronic conditions reported?**  *Yes* if the study reported the number of individuals excluded explicitly for having other chronic conditions. Do not list numbers reported for specific chronic conditions here. Ex: 5 patients were excluded for comorbid chronic conditions. |
| **Is a justification for comorbidities exclusion provided?**  *No*- did not provide rationale for excluding individuals with comorbidities *Yes*- did provide rationale for excluding individuals with comorbidities  NA (Not Applicable) – comorbidities were not excluded |
| **Did trial exclude individuals with specific chronic conditions?**  This does not include the chronic condition(s) shared by all participants (e.g. trial including septic patients with CKD)  *No*  Did not report exclusion of individuals specifically based on any chronic conditions, even if individuals were excluded on the basis of having more than one chronic condition, but no specific condition is named  *Yes*  Individuals were excluded from the trial on the basis of having one or more of the 20 chronic conditions, which is specifically named or determined by diagnostic criteria for the condition Ex: Individuals with depression were excluded from the trial or individuals with systolic blood pressure >140 were excluded from the trial |
| **Is this exclusion based on ability to participate in the study?**  *Yes* if exclusion is based on the patient’s physical or mental capacity to participate in the study Ex: Excludes individuals with dementia due to inability to complete intervention components |
| **Which chronic conditions were subject to exclusions?**  Copy & paste all conditions that limit an individual’s eligibility for the study, aside from the chronic condition(s) shared by all participants in the study |
| **Is the number of individuals excluded for having chronic conditions reported?**  *Yes* if the number excluded for this specific conditions is reported |
| **Were there any age restrictions for trial participants (aside from 18 years or older)?**  *No* - the only age restriction for the trial is that participants had to be at least 18 years old *Yes*- additional age restrictions were used, which further limited eligibility |
| **What type of age exclusion?**  *Minimum Age* Adult participants had to be at least *X* years of age *Maximum age*  *Maximum Age*  Adult participants could not be over *X* years of age |
| **Excluded those above age:**  Upper age restriction |
| **Is a participant flow diagram presented?**  *Yes* if the article includes a diagram that details the process of patient selection This usually includes the number of individuals screened, ineligible, enrolled, randomized and followed up |
| **Are chronic conditions included in the participant characteristics?**  This may be reported in the Subjects section or in Table 1  *Yes* if article reported one of the following:   - Number of study participants who also had another chronic condition not necessary for inclusion - Mean number of chronic conditions per participant - Charlson Comorbidity index mean and standard deviation - Other comorbidity measures/classification frameworks |
| **Can the inclusion of individuals with multiple chronic conditions be inferred?**  *Yes* if participant characteristics inferring the presence of multiple chronic conditions were reported Ex: A trial lists the number of participants taking anti- hypertensives. It can be inferred that these individuals have multiple chronic conditions because they have both cancer and hypertension. |
| **Is this description general or condition specific?**  Check all that apply *General*- study broadly described a group of participants with multiple chronic conditions (ex: # of participants with comorbid conditions) *Condition Specific*- study specifically described group(s) of participants with certain chronic condition(s) (ex: # of participants with hypertension) |
| **Which specific conditions were reported or inferred in the participant characteristics?**  Introduce all conditions other than the condition(s) necessary for inclusion in the study (table 1-10) + number and percentage (intervention and control arms) |
| **How many additional specific chronic conditions were reported or inferred?**  The total number of specific chronic conditions that were included or inferred in the participant characteristics. |
| **Are any of the following statistics regarding participants with comorbidities/multimorbidity reported?**  Ex: *Number* – number of enrolled participants with one or more comorbid chronic conditions *Percentage* - percentage of enrolled participants (out of study total) with one or more comorbid chronic conditions *Mean -* the mean number of chronic conditions per participant *Charlson comorbidity index-* a specific index for comorbidities, would be reported as a mean and standard deviation in participant characteristics  Other comorbidity index tool (specify which) *Not Reported*- number or percentage of participants with one or more comorbid chronic conditions is not reported |
| **What is the data source of the comorbidities/multimorbidity reported?**  *Primary care data*  *Hospital data*  *Prescribing data*  *Self-reporting*  *Not reported*  *N/A* |
| **Is comorbidity information considered in analysis?**  *Yes* if Primary outcomes were compared between individuals with and without comorbidities |

Additional file 4

## **Additional file 4**: Lists of conditions reported by trials that were mapped to a list of conditions adapted from “Measuring multimorbidity in research: a Delphi consensus study” (Ho et al, 2022).

| **Comorbidity name (as reported in trials)** | **Mapped to: list of conditions to always or usually include** | **Body system (based on ICD-10 chapters)** |
| --- | --- | --- |
| ACE Inhibitor | - | - |
| Acute kidney injury | - | - |
| Acute myocardial infarction | Coronary artery disease | Cardiovascular disease |
| Alcoholism | Drug or alcohol misuse | Mental and behavioural disorder |
| Allergies | - | - |
| Aortic insufficiency | Heart valve disorders | Cardiovascular disease |
| Anaemia | Anaemia | Haematological disorder |
| Angina pectoris | Coronary artery disease | Cardiovascular disease |
| Angioimmunoblastic lymphadenopathy | - | - |
| Angiotensin receptor blocker | - | - |
| Arrhythmia | Arrhythmia | Cardiovascular disease |
| Asthma | Asthma | Respiratory disease |
| Asthma or COPD | - | Respiratory disease |
| Asthma/chronic lung diseases | - | Respiratory disease |
| Atrial fibrillation | Arrhythmia | Cardiovascular disease |
| Autoimmune disease | - | - |
| Autoimmune vasculitis | - | - |
| Beta blocker | - | - |
| Calcium channel blocker | - | - |
| Cancer | - | Cancer |
| Cancer and/or immunosuppression | - | Cancer |
| Cancer or autoimmune disease | - | Cancer |
| Cardiac disease | - | Cardiovascular disease |
| Cardiac failure | Heart failure | Cardiovascular disease |
| Cardiomyopathy | Heart failure | Cardiovascular disease |
| Cardiovascular disease | - | Cardiovascular disease |
| Cellulitis | - | - |
| Central nervous system diseases | - | Neurological disease |
| Cerebral haemorrhage | Stroke | Cardiovascular disease |
| Cerebrovascular disease | - | Cardiovascular disease |
| Chronic cardiac disease | - | Cardiovascular disease |
| Chronic heart failure | Heart failure | Cardiovascular disease |
| Chronic haemodialysis | Chronic kidney disease | Urogenital disorder |
| Chronic liver disease | Chronic liver disease | Digestive disease |
| Chronic lung disease | - | Respiratory disease |
| Chronic lymphocytic leukemia | - | Cancer |
| Cirrhosis | Chronic liver disease | Digestive disease |
| CKD | Chronic kidney disease | Urogenital disorder |
| Cognitive impairment/dementia | Dementia | Mental and behavioural disorder |
| Colon Cancer | - | Cancer |
| Congestive heart failure | Heart failure | Cardiovascular disease |
| Congestive or Ischaemic heart disease | - | Cardiovascular disease |
| Convulsions, types of tumors, paraplegia | - | - |
| COPD | COPD | Respiratory disease |
| COPD/Asthma | - | Respiratory disease |
| Coronary artery disease | Coronary artery disease | Cardiovascular disease |
| Corticosteroid use | - | - |
| Deep venous thrombosis | Venous thromboembolic disease | Cardiovascular disease |
| Dementia | Dementia | Mental and behavioural disorder |
| Diabetes | Diabetes | Metabolic and endocrine disease |
| Dyslipidemia | - | - |
| End stage renal disease | Chronic kidney disease | Urogenital disorder |
| Endocrine diseases | - | Metabolic and endocrine disease |
| Endometrial cancer | - | Cancer |
| Epilepsy | Epilepsy | Neurological disease |
| ESRD on haemodialysis | Chronic kidney disease | Urogenital disorder |
| Found obtunded | - | - |
| Furosemide | - | - |
| Gastric bypass | - | - |
| Gastrointestinal | - | Digestive disease |
| Gastrointestinal hemorrhage | - | - |
| Gastrointestinal malignancy | - | Cancer |
| Glycuresis | - | - |
| Gout | Gout | Musculoskeletal disease |
| Hansen’s disease | - | - |
| Haematological malignancy | - | Cancer |
| Head and neck cancer | - | Cancer |
| Heart disease | - | Cardiovascular disease |
| Heart failure | Heart failure | Cardiovascular disease |
| Heart failure or myocardial infarction | - | Cardiovascular disease |
| Heart valve disease | Heart valve disorders | Cardiovascular disease |
| Hematologic | - | Haematological disorder |
| Hematologic cancer | - | Cancer |
| Hematologic or metastatic cancer | - | Cancer |
| Hematological disease | - | Haematological disorder |
| Hematological/anticoagulated | - | Haematological disorder |
| Hemopathy | - | Haematological disorder |
| Hemodialysis | - | - |
| Hepatorenal syndrome | - | - |
| Hepatitis C virus positive | - | - |
| History of illicit drug use | Drug or alcohol misuse | Mental and behavioural disorder |
| HIV | HIV | Infectious disease |
| HIV with AIDS | HIV | Infectious disease |
| Hypercholesterolemia | - | - |
| Hyperlipidaemia | - | - |
| Hypertension | Hypertension | Cardiovascular disease |
| Hyponatremia | - | - |
| Hypothyroidism | Thyroid disorders | Metabolic and endocrine disease |
| Immunosuppression | - | - |
| Immune disease | - | - |
| Infectious disease | - | Infectious disease |
| Influenza | - | - |
| Intoxication | - | - |
| Intravenous drug abuse | Drug or alcohol misuse | Mental and behavioural disorder |
| Intraventricular heart defect | Congenital disease and chromosomal abnormalities | Congenital disease |
| Irritable Bowel Disease | Inflammatory bowel disease | Digestive disease |
| Ischaemic heart disease | Coronary artery disease | Cardiovascular disease |
| Ischaemic heart disease or heart failure | - | Cardiovascular disease |
| Long-term dialysis | Chronic kidney disease | Urogenital disorder |
| Lung cancer | - | Cancer |
| Lymphoma, leukemia, multiple myeloma | - | Cancer |
| Malnourished | - | - |
| MDR infection | - | - |
| Metabolic | - | Metabolic and endocrine disease |
| Multiple sclerosis | Multiple sclerosis | Neurological disease |
| Myocardial infarction | Coronary artery disease | Cardiovascular disease |
| Neoplasia hematologic/lymphoreticular | - | Cancer |
| Neurological disease | - | Neurological disease |
| Neurological/muscular disease | - | Neurological disease |
| Non-Hodgkins lymphoma | - | Cancer |
| Obesity | - | - |
| Obstructive nephrolithiasis pyelonephritis | - | - |
| Organ transplant | - | - |
| Osteoarthritis | Osteoarthritis | Musculoskeletal disease |
| Osteoporosis | Osteoporosis | Musculoskeletal disease |
| Other | - | - |
| Other pulmonary disorder | - | Respiratory disease |
| Pancreatitis | Chronic pancreatitis |  |
| Peripheral vascular disease | Peripheral arterial disease | Cardiovascular disease |
| Pneumonia | - | - |
| Previous coronary intervention | Coronary artery disease | Cardiovascular disease |
| Previous vascular intervention (non-coronary) | - | - |
| Prostate cancer | - | Cancer |
| Psychiatry disease | - | Mental and behavioural disorder |
| Pulmonary embolism | - | - |
| Recent trauma | - | - |
| Rheumaimmune systemic diseases | - | - |
| Rheumatic disease | Connective tissue disease | Musculoskeletal disease |
| Rheumatoid arthritis | Connective tissue disease | Musculoskeletal disease |
| Schizophrenia | Schizophrenia | Mental and behavioural disorder |
| Secondary pulmonary hypertension | - | - |
| Seizures | Epilepsy | Neurological disease |
| Severe chronic heart failure   (NYHA group 3-4) | Heart failure | Cardiovascular disease |
| Severe aortic stenosis | Heart valve disorders | Cardiovascular disease |
| Severe ankylosing spondylitis | Connective tissue disease | Musculoskeletal disease |
| Smoking | - | - |
| Stroke | Stroke | Cardiovascular disease |
| Substance misuse | Drug or alcohol misuse | Mental and behavioural disorder |
| Supraventricular arrhythmia | Arrythmia | Cardiovascular disease |
| Systemic mastocytosis | - | - |
| Thromboembolic disease | Venous thromboembolic disease | Cardiovascular disease |
| Thromboembolism | Venous thromboembolic disease | Cardiovascular disease |
| Thrombophilia | - | - |
| Thrombosis | Venous thromboembolic disease | Cardiovascular disease |
| Thyroid disorders | Thyroid disorders | Metabolic and endocrine disease |
| Toxic epidermal necrolysis | - | - |
| Transient ischemic attack | Stroke | Cardiovascular disease |
| Trauma | - | - |
| Tuberculosis | Tuberculosis | Infectious disease |
| Uremia | - | - |
| Urinary disease | - | Urogenital disorder |
| Vasculitis | - | - |
| Ventricular arrhythmia | Arrythmia | Cardiovascular disease |

## **Additional file 6:** Study characteristics of all trials (N=209) reporting comorbidity information, stratified by time period. The inclusion criteria were: (a) primary report of a RCT testing the efficacy or effectiveness of any intervention in patients with sepsis and/or septic shock (b) the study reports original data (protocols, posttrial follow-up studies, secondary or separate subgroup analyses were excluded); (c) the trial was published in English; (d) the RCT was conducted in ICU; (e) the RCT enrolled only adult subjects (>18 years) and (f) the study reported at least one comorbidity information (number and/or percentage) on trial participants.

| **Category** | **Variable** | **1992–2000**  (N = 7) | **2001–2015**  (N = 58) | **2016–2025**  (N = 144) | **Total**  (N = 209) |
| --- | --- | --- | --- | --- | --- |
| Trial Registration | No | 7 (100%) | 23 (39.7%) | 22 (15.3%) | 52 (24.9%) |
|  | Yes - clinicaltrials.gov | 0 | 30 (51.7%) | 85 (59%) | 115 (54.8%) |
|  | Yes - other registry | 0 | 5 (8.6%) | 37 (25.7%) | **42 (20.1%)** |
| Region | Asia/Pacific | 1 (14.3%) | 16 (27.6%) | 85 (59%) | 102 (48.8%) |
|  | Europe | 2 (28.6%) | 20 (34.5%) | 29 (20.1%) | 51 (24.4%) |
|  | North America | 4 (57.1%) | 10 (17.2%) | 11 (7.6%) | 25 (12%) |
|  | Africa | 0 | 1 (1.7%) | 8 (5.6%) | 9 (4.3%) |
|  | Latin America | 0 | 7 (12.1%) | 7 (4.9%) | 14 (6.7%) |
|  | More than one | 0 | 4 (6.0%) | 4 (2.8%) | 8 (3.8%) |
| Number of participants | Median (IQR) | 51 (24.5-120) | 97 (53.5-518) | 90.5 (59.5-159.8) | 90.5 (56.3 – 209.8) |
| Sex Reported | Yes | 7 (100%) | 58 (100%) | 143 (99.3%) | 208 (99.52%) |
| Age reported | Mean | 7 (100%) | 40 (69%) | 88 (61.1%) | 135 (64.6%) |
|  | Median | 0 | 17 (29.3%) | 55 (38.2 %) | 72 (34.4%) |
|  | Mean and Median | 0 | 0 | 1 (0.7%) | 1 (0.5%) |
|  | Range only | 0 | 0 | 1 (0.7%) | 1 (0.5%) |
| Ethnicity Reported |  | 3 (42.9%) | 13 (22.4%) | 15 (10.4%) | 31 (14.8%) |
| Number of comorbidities reported | Median (IQR) | 8 (5.5 – 8.5) | 7 (4 - 8) | 6 (4 - 7) | 6 (3 - 9) |
| Number of general comorbidities listed as exclusion criteria in each trial | 0 comorbidities | 1 (14.3%) | 12 (20.3%) | 33 (22.9%) | 46 (21.9%) |
|  | 1-2 comorbidities | 3 (42.85%) | 24 (41%) | 66 (45.8%) | 93 (44.3%) |
|  | 3-5 comorbidities | 2 (28.6%) | 19 (32.8%) | 38 (26.4%) | 59 (28.1%) |
|  | 6-9 comorbidities | 1 (14.3%) | 4 (6.9%) | 7 (4.9%) | 12 (5.7%) |

## Additional file 7:


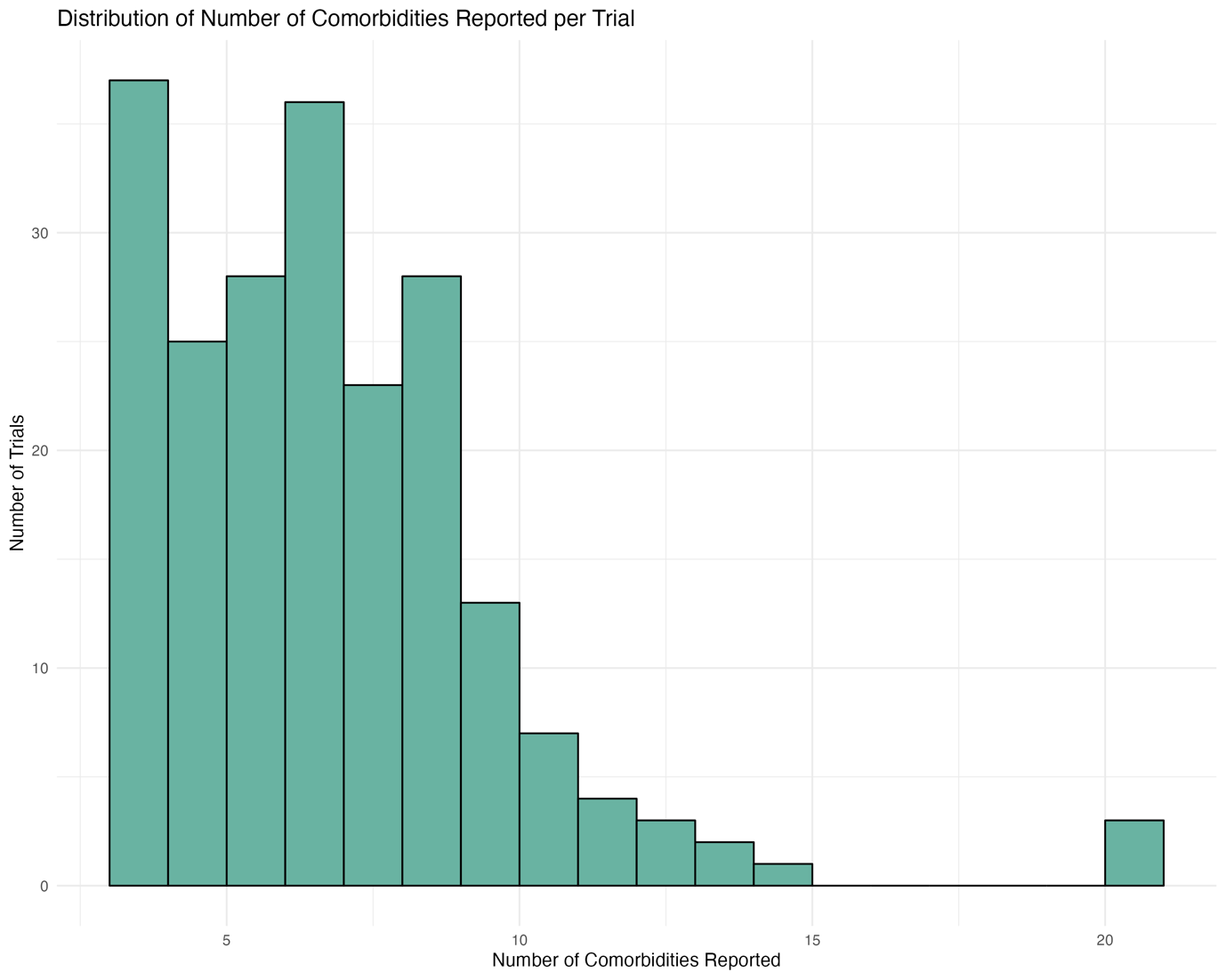


Figure S7: Histogram showing the number of distinct comorbidities reported per trial among the 209 included trials. The median of reported comorbidities was 6 (IQR: 3-9).

Table S7: The frequency of comorbidity reporting in trials stratified by time period. The median (IQR) number of comorbidities reported was 8 (6–9) in 1992–2000, 7 (5.7–8.2) in 2001–2015, and 6 (4–7) in 2016–2025. The number of comorbidities reported per trial differed significantly across time periods (Kruskal–Wallis χ²(2)=6.25, *p*=0.040)

| **Comorbidity** | **1992–2000**  (N = 7) | **2001–2015**  (N = 58) | **2016–2025**  (N = 144) | **Total**  (N = 209) |
| --- | --- | --- | --- | --- |
| Diabetes | 6 (85.7%) | 50 (86.2%) | 124 (86.1%) | 180 (86.1%) |
| Hypertension | 1 (14.3%) | 33 (56.9%) | 102 (70.8%) | 136 (65.1%) |
| Chronic kidney disease | 4 (57.1%) | 32 (55.2%) | 81 (56.2%) | 117 (56%) |
| Cancer | 7 (100%) | 39 (67.2%) | 65 (45.1%) | 111 (53.1%) |
| Copd | 6 (85.7%) | 39 (67.2%) | 62 (43.1%) | 107 (51.2%) |
| Chronic liver disease | 4 (57.1%) | 31 (53.4%) | 59 (41%) | 94 (45%) |
| Coronary artery disease | 5 (71.4%) | 21 (36.2%) | 65 (45.1%) | 91 (43.5%) |
| Heart failure | 1 (14.3%) | 31 (53.4%) | 55 (38.2%) | 87 (41.6%) |
| Cardiovascular disease | 0 (0%) | 14 (24.1%) | 31 (21.5%) | 45 (21.5%) |
| Respiratory disease | 0 (0%) | 10 (17.2%) | 32 (22.2%) | 42 (20.1%) |
| Stroke | 0 (0%) | 9 (15.5%) | 20 (13.9%) | 29 (13.9%) |
| Drug or alcohol misuse | 5 (71.4%) | 9 (15.5%) | 7 (4.9%) | 21 (10%) |
| Neurological disease | 0 (0%) | 6 (10.3%) | 6 (4.2%) | 12 (5.7%) |
| Arrhythmia | 0 (0%) | 1 (1.7%) | 10 (6.9%) | 11 (5.3%) |
| Peripheral arterial disease | 0 (0%) | 2 (3.4%) | 6 (4.2%) | 8 (3.8%) |
| Chronic pancreatitis | 2 (28.6%) | 4 (6.9%) | 1 (0.7%) | 7 (3.3%) |
| Thyroid disorders | 0 (0%) | 0 (0%) | 7 (4.9%) | 7 (3.3%) |
| Asthma | 1 (14.3%) | 1 (1.7%) | 3 (2.1%) | 5 (2.4%) |
| Dementia | 1 (14.3%) | 0 (0%) | 4 (2.8%) | 5 (2.4%) |
| Digestive disease | 0 (0%) | 1 (1.7%) | 4 (2.8%) | 5 (2.4%) |
| Venous thromboembolic disease | 0 (0%) | 4 (6.9%) | 1 (0.7%) | 5 (2.4%) |
| Haematological disorder | 0 (0%) | 1 (1.7%) | 3 (2.1%) | 4 (1.9%) |
| Hiv | 0 (0%) | 1 (1.7%) | 3 (2.1%) | 4 (1.9%) |
| Epilepsy | 1 (14.3%) | 0 (0%) | 2 (1.4%) | 3 (1.4%) |
| Heart valve disorders | 0 (0%) | 1 (1.7%) | 2 (1.4%) | 3 (1.4%) |
| Connective tissue disease | 0 (0%) | 0 (0%) | 2 (1.4%) | 2 (1%) |
| Metabolic and endocrine disease | 0 (0%) | 0 (0%) | 2 (1.4%) | 2 (1%) |
| Osteoarthritis | 0 (0%) | 0 (0%) | 2 (1.4%) | 2 (1%) |
| Osteoporosis | 0 (0%) | 0 (0%) | 2 (1.4%) | 2 (1%) |
| Anaemia | 0 (0%) | 1 (1.7%) | 0 (0%) | 1 (0.5%) |
| Congenital disease and chromosomal abnormalities | 0 (0%) | 0 (0%) | 1 (0.7%) | 1 (0.5%) |
| Gout | 0 (0%) | 1 (1.7%) | 0 (0%) | 1 (0.5%) |
| Infectious diseases | 0 (0%) | 1 (1.7%) | 0 (0%) | 1 (0.5%) |
| Inflammatory bowel disease | 0 (0%) | 0 (0%) | 1 (0.7%) | 1 (0.5%) |
| Mental and behavioural disorder | 0 (0%) | 0 (0%) | 1 (0.7%) | 1 (0.5%) |
| Multiple sclerosis | 0 (0%) | 0 (0%) | 1 (0.7%) | 1 (0.5%) |
| Schizophrenia | 1 (14.3%) | 0 (0%) | 0 (0%) | 1 (0.5%) |
| Tuberculosis | 0 (0%) | 1 (1.7%) | 0 (0%) | 1 (0.5%) |
| Urogenital disorder | 0 (0%) | 0 (0%) | 1 (0.7%) | 1 (0.5%) |

## **Additional file 8**: Trials that report participant characteristics inferring the presence of multiple chronic conditions

| **Study ID** | **Trial registration** | **Region** | **Number of participants** | **Multimorbidity reporting** |
| --- | --- | --- | --- | --- |
| Tongyoo et al, 2016 | Yes - clinicaltrials.gov | Asia/Pacific | 206 | Patients are classified in two groups: less than or more than 2 comorbidities |
| Inthorn et al, 1998 | No | Europe | 29 | All comorbidities are reported for each individual patient |
| Olson et al, 1996 | No | North America | 16 | All comorbidities are reported for each individual patient |
| Fowler et al, 2014 | Yes - clinicaltrials.gov | North America | 24 | All comorbidities are reported for each individual patient |

## **Additional file 9**: Trials that report comorbidities using a specific framework or comorbidity reporting tool

| **Study ID** | **Trial registration** | **Region** | **Number of participants** | **Comorbidity framework used** |
| --- | --- | --- | --- | --- |
| Deliberato et al, 2013 | Yes - clinicaltrials.gov | Latin America | 81 | Charlson Comorbidity Index score |
| De Pascale et al, 2020 | Yes - clinicaltrials.gov | Europe | 120 | Charlson Comorbidity Index score |
| Timsit et al, 2016 | Yes - clinicaltrials.gov | Europe | 260 | Chronic health APACHE II score points |
| Hajjar et al, 2019 | Yes - clinicaltrials.gov | Europe | 250 | Charlson Comorbidity Index score |
| Lamontagne et al, 2022 | Yes - clinicaltrials.gov | More than one | 862 | Charlson Comorbidity Index score |
| Salehi et al, 2023 | No | Asia/Pacific | 30 | Charlson Comorbidity Index score |
| Bergamin et al, 2017 | Yes - clinicaltrials.gov | Latin America | 300 | Charlson Comorbidity Index score |
| Bloos et al, 2016 | Yes - clinicaltrials.gov | Europe | 1180 | Charlson Comorbidity Index score |
| Hung et al, 2023 | No | Asia/Pacific | 132 | Charlson Comorbidity Index score |
| Werdan et al, 2007 | No | Europe | 624 | Chronic health APACHE II score points |
| Almeida et al, 2025 | Yes - clinicaltrials.gov | Latin America | 167 | Charlson Comorbidity Index score |
| Dargent et al, 2025 | Yes - clinicaltrials.gov | Europe | 32 | Charlson Comorbidity Index score |

## **Additional file 10**: Trials that performed subgroup analysis based on the presence of comorbidities/multimorbidity

| **Study ID** | **Trial registration** | **Region** | **Number of participants** | **Comorbidity subgroup analysis** |
| --- | --- | --- | --- | --- |
| Corl et al, 2019 | Yes - clinicaltrials.gov | North America | 109 | Subgroup analysis included two groups: presence or absence of CKD |
| Payen et al, 2015 | Yes - clinicaltrials.gov | Europe | 243 | Subgroup analysis included two groups: presence or absence of any comorbidities |
| Stephens et al, 2008 | Yes - clinicaltrials.gov | Asia/Pacific | 164 | Subgroup analysis included two groups: presence or absence of comorbidities associated with neutrophil dysfunction |
| Tongyoo et al, 2016 | Yes - clinicaltrials.gov | Asia/Pacific | 206 | Subgroup analysis included two groups, based on the number of comorbidities (≤ 2 or  ≥ 3) |
| Wu et al, 2025 | Yes - clinicaltrials.gov | Asia/Pacific | 1106 | Subgroup analysis was conducted based on the presence/absence of six chronic conditions |

## **Additional file 11:** Mapped general comorbidity categories used as exclusion criteria. The number of trials represent trials (out of total = 209) that list a comorbidity category as an exclusion criteria.

| **Comorbidity category** | **Number of trials (%)** |
| --- | --- |
| Cardiovascular disease | 70 (33.5%) |
| Urogenital disorder | 66 (31.6%) |
| Digestive disease | 58 (27.8%) |
| Disorders of the Immune System | 56 (26.8%) |
| Cancer | 56 (26.8%) |
| Haematological disorder | 38 (18.2%) |
| Infectious disease | 32 (15.3%) |
| Mental and behavioural disorder | 24 (11.5%) |
| Respiratory disease | 24 (11.5%) |
| Metabolic and endocrine disease | 15 (7.2%) |
