# Supplementary material for: Prevalence and representation of comorbidities and multimorbidity in randomised controlled trials in sepsis or septic shock: a systematic review
Source: Crit Care. 2026 May 6;30:339. doi: 10.1186/s13054-026-06049-y (PMC13312557; doi:10.1186/s13054-026-06049-y)
Supplement: Supplementary file 2 — Supplementary Material 2. [file 13054_2026_6049_MOESM2_ESM.docx]

# **Additional file 5**

## **Table S5A: Characteristics of the randomized controlled trials (N = 591) included in the systematic review**

| **Study title** | **Journal** | **Study ID** | **Sample size** | **Comorbidities reported (Yes/No)** | **Intervention category** | **Region** | **International (Yes/No)** | **Multicenter (Yes/No)** | **Registration (Clinicaltrials.gov/Other/No)** | **Protocol available (Yes/No)** | **Primary outcome** | **Positive outcome (Yes/No)** | **Funding** |
| --- | --- | --- | --- | --- | --- | --- | --- | --- | --- | --- | --- | --- | --- |
| (1,3)-beta-D-Glucan-guided antifungal therapy in adults with sepsis: the CandiSep randomized clinical trial. | Intensive Care Med | Bloos 2022 | 339 | No | Therapeutic interventions | Europe | No | Yes | Yes - clinicaltrials.gov | Yes | Mortality | No | Non-industry |
| (1,3)-beta-D-Glucan-based empirical antifungal interruption in suspected invasive candidiasis: a randomized trial. | Crit Care | DePascale 2020 | 120 | Yes | Monitoring systems | Europe | No | No | Yes - clinicaltrials.gov | Yes | Other | Yes | Not reported |
| A dose-finding study of methylene blue to inhibit nitric oxide actions in the hemodynamics of human septic shock | Nitric Oxide | Juffermans 2010 | 15 | No | Therapeutic interventions | Europe | No | Yes | No | No | Other | Yes | Not reported |
| A modified goal-directed protocol improves clinical outcomes in intensive care unit patients with septic shock: a randomized controlled trial | Shock | Lin 2006 | 224 | Yes | Care bundles | Asia/Pacific | No | No | No | No | Mortality | Yes | Non-industry |
| A multicenter randomized trial of atorvastatin therapy in intensive care patients with severe sepsis | Am J Respir Crit Care Med | Kruger 2013 | 250 | Yes | Therapeutic interventions | Asia/Pacific | Yes | Yes | Yes - Other | Yes | Other | No | Non-industry |
| A multicenter randomized trial of continuous versus intermittent beta-lactam infusion in severe sepsis | Am J Respir Crit Care Med | Dulhunty 2015 | 432 | No | Therapeutic interventions | Asia/Pacific | Yes | Yes | Yes - Other | Yes | Other | No | Non-industry |
| A multicenter, open-label, prospective, randomized, dose-ranging pharmacokinetic study of the anti-TNF-alpha antibody afelimomab in patients with sepsis syndrome | Intensive Care Med | Gallagher 2001 | 48 | No | Therapeutic interventions | North America | No | Yes | No | No | Other | Yes | Industry |
| A new immunomodulatory therapy for severe sepsis: ulinastatin Plus Thymosin {alpha} 1 | J Intensive Care Med | Li 2009 | 56 | No | Therapeutic interventions | Asia/Pacific | No | No | No | No | Mortality | Yes | Not reported |
| A phase 2 randomized, placebo-controlled trial of inulin for the prevention of gut pathogen colonization and infection among patients admitted to the intensive care unit for sepsis. | Crit Care | Park 2025 | 90 | No | Monitoring systems | North America | No | No | Yes - clinicaltrials.gov | Yes | Other | No | Non-industry |
| A phase I trial evaluating the safety, tolerability, pharmacokinetics and pharmacodynamics of intravenously administered low-anticoagulant heparin (M6229) in critically ill sepsis patients | Intensive Care Medicine Experimental | vanMourik 2025 | 10 | No | Therapeutic interventions | Europe | No | No | Yes - clinicaltrials.gov | Yes | Other | Yes | Industry |
| A phase I trial of low-dose inhaled carbon monoxide in sepsis-induced ARDS | JCI Insight | Fredenburgh 2018 | 12 | No | Therapeutic interventions | North America | No | Yes | Yes - clinicaltrials.gov | Yes | Other | Yes | Non-industry |
| A phase II randomized, controlled trial of continuous hemofiltration in sepsis | Crit Care Med | Cole 2002 | 24 | No | Other strategies | Asia/Pacific | No | No | No | No | Other | No | Industry |
| A phase II, single-center, double-blind, randomized placebo-controlled trial to explore the efficacy and safety of intravenous melatonin in surgical patients with severe sepsis admitted to the intensive care unit | J Pineal Res | Mansilla-Rosello 2023 | 14 | No | Therapeutic interventions | Europe | No | No | Yes - Other | Yes | Other | Yes | Non-industry |
| A Pilot Double-Blind Placebo-Controlled Randomized Clinical Trial to Investigate the Effects of Early Enteral Nutrients in Sepsis | Crit Care Explor | Shah 2021 | 58 | No | Other strategies | North America | No | No | Yes - clinicaltrials.gov | Yes | Other | No | Non-industry |
| A Pilot Feasibility Randomized Controlled Trial of Intravenous Vitamin C in Adults with Sepsis in the Intensive Care Unit: the Lessening Organ Dysfunction with Vitamin C-India (LOVIT-India) Trial | Indian J Crit Care Med | Vijayaraghavan 2023 | 60 | No | Therapeutic interventions | Asia/Pacific | No | Yes | Yes - Other | Yes | Other | Yes | Industry |
| A pilot randomised controlled trial in intensive care patients comparing 7 days' treatment with empirical antibiotics with 2 days' treatment for hospital-acquired infection of unknown origin. | Health technology assessment | Scawn 2012 | 46 | No | Therapeutic interventions | Asia/Pacific | No | Yes | Yes - Other | Yes | Mortality | No | Non-industry |
| A pilot randomized controlled trial of comparison between extended daily hemodialysis and continuous veno-venous hemodialysis in patients of acute kidney injury with septic shock | Indian J Crit Care Med | Mishra 2017 | 60 | Yes | Other strategies | Asia/Pacific | No | No | Yes - Other | Yes | Other | No | Not reported |
| A pilot randomized study comparing high and low volume hemofiltration on vasopressor use in septic shock | Intensive Care Med | Boussekey 2008 | 20 | No | Other strategies | Europe | No | No | No | No | Other | Yes | Non-industry |
| A pilot study of coupled plasma filtration with adsorption in septic shock | Crit Care Med | Ronco 2002 | 10 | No | Other strategies | Europe | No | No | No | No | Other | Yes | Industry |
| A pilot study of high-adsorption hemofiltration in human septic shock | Int J Artif Organs | Haase 2007 | 12 | No | Other strategies | Asia/Pacific | No | No | No | No | Other | Yes | Non-industry |
| A pilot-controlled study of a polymyxim B-immobilized hemoperfusion cartridge in patients with severe sepsis secondary to intra-abdominal infection | Shock | Vincent 2005 | 19 | No | Other strategies | Europe | Yes | Yes | No | No | Other | No | Industry |
| A placebo-controlled, double-blind, dose-escalation study to assess the safety, tolerability and pharmacokinetics/pharmacodynamics of single and multiple intravenous infusions of AZD9773 in patients with severe sepsis and septic shock | Crit Care | Morris 2012 | 71 | Yes | Monitoring systems | North America | No | Yes | Yes - clinicaltrials.gov | Yes | Other | Yes | Industry |
| A prospective open label randomized noninferiority trial to compare the efficacy and safety of monotherapy with noradrenaline and terlipressin in patients of cirrhosis with septic shock admitted to the intensive care unit (nct01836224) | Hepatology | Choudhury 2014 | 40 | No | Therapeutic interventions | Asia/Pacific | No | No | Yes - clinicaltrials.gov | Yes | Other | Yes | Not reported |
| A prospective, randomised clinical study comparing triple therapy regimen to hydrocortisone monotherapy in reducing mortality in septic shock patients | Int J Clin Pract | Hussein 2021 | 94 | No | Therapeutic interventions | Africa | No | No | Yes - clinicaltrials.gov | Yes | Mortality | No | Non-industry |
| A randomised-controlled trial (TARGET-C) of high vs. low target mean arterial pressure in patients with cirrhosis and septic shock | J Hepatol | Maiwall 2023 | 150 | Yes | Therapeutic interventions | Asia/Pacific | No | No | Yes - clinicaltrials.gov | Yes | Mortality | No | Not reported |
| A randomized and controlled trial of the effect of treatment aimed at maximizing oxygen delivery in patients with severe sepsis or septic shock | Chest | Alia 1999 | 32 | No | Care bundles | Europe | No | No | No | No | Mortality | No | Non-industry |
| A randomized controlled trial investigating the effects of parenteral fish oil on survival outcomes in critically ill patients with sepsis: a pilot study | Journal of Parenteral and Enteral Nutrition | Hall 2015 | 60 | Yes | Other strategies | Europe | No | No | No | No | Other | Yes | Industry |
| A randomized phase II trial of granulocyte-macrophage colony-stimulating factor therapy in severe sepsis with respiratory dysfunction | Am J Respir Crit Care Med | Presneill 2002 | 10 | No | Therapeutic interventions | Asia/Pacific | No | No | No | No | Mortality | No | Industry |
| A randomized pilot study of parenteral glutamine supplementation in severe sepsis | Crit Care Shock | Poulose 2017 | 20 | No | Other strategies | Asia/Pacific | No | No | Yes - clinicaltrials.gov | Yes | Mortality | No | Non-industry |
| A Randomized Trial of Mycobacterium w in Severe Presumed Gram-Negative Sepsis | Chest | Sehgal 2021 | 202 | No | Therapeutic interventions | Asia/Pacific | No | Yes | Yes - clinicaltrials.gov | Yes | Mortality | Yes | Non-industry |
| A randomized trial of Mycobacterium w in severe sepsis | J Crit Care | Sehgal 2015 | 50 | Yes | Therapeutic interventions | Asia/Pacific | No | No | No | No | Mortality | No | Non-industry |
| A randomized trial to compare procalcitonin and C-reactive protein in assessing severity of sepsis and in guiding antibacterial therapy in Egyptian critically ill patients | Irish Journal of Medical Science | Ali 2021 | 60 | Yes | Monitoring systems | Africa | No | No | Yes - clinicaltrials.gov | Yes | Other | Yes | Non-industry |
| A randomized-controlled trial of arginine infusion in severe sepsis on microcirculation and metabolism | Clin Nutr | Luiking 2020 | 18 | No | Therapeutic interventions | Europe | No | No | Yes - Other | Yes | Other | No | Industry |
| A randomized, double-blind, placebo-controlled trial of TAK-242 for the treatment of severe sepsis | Crit Care Med | Rice 2010 | 274 | No | Therapeutic interventions | More than one | Yes | Yes | No | No | Other | No | Industry |
| A randomized, double-blind, placebo-controlled, Phase 2b study to evaluate the safety and efficacy of recombinant human soluble thrombomodulin, ART-123, in patients with sepsis and suspected disseminated intravascular coagulation | Crit Care Med | Vincent 2013 | 750 | No | Therapeutic interventions | More than one | Yes | Yes | No | No | Mortality | No | Industry |
| A second large controlled clinical study of E5, a monoclonal antibody to endotoxin: results of a prospective, multicenter, randomized, controlled trial. The E5 Sepsis Study Group | Crit Care Med | Bone 1995 | 847 | No | Therapeutic interventions | Asia/Pacific | No | Yes | No | No | Mortality | No | Industry |
| ACETATE RINGER'S SOLUTION VERSUS NORMAL SALINE SOLUTION IN SEPSIS: A RANDOMIZED, CONTROLLED TRIAL. | Shock | Zhang 2024 | 225 | Yes | Other strategies | Asia/Pacific | No | No | Yes - clinicaltrials.gov | Yes | Other | No | Non-industry |
| Acetylsalicylic Acid Treatment in Patients With Sepsis and Septic Shock: A Phase 2, Placebo-Controlled, Randomized Clinical Trial. | Crit Care Med | Almeida 2025 | 167 | Yes | Therapeutic interventions | Latin America | No | Yes | Yes - clinicaltrials.gov | Yes | Other | No | Non-industry |
| Acute bag-valve breathing maneuvers plus manual chest compression is safe during stable septic shock: a randomized clinical trial | Rev Bras Ter Intensiva | Blattner 2017 | 32 | No | Other strategies | Latin America | No | No | Yes - Other | No | Other | No | Non-industry |
| Acute haemodynamic effects of a hypertonic saline/dextran solution in stable patients with severe sepsis | Intensive Care Med | Oliveira 2002 | 16 | No | Other strategies | Latin America | No | No | No | No | Other | Yes | Not reported |
| Addition of terlipressin to norepinephrine in septic shock and effect of renal perfusion: a pilot study | Renal failure | Wang 2022 | 22 | Yes | Therapeutic interventions | Asia/Pacific | No | No | Yes - clinicaltrials.gov | Yes | Other | Yes | Non-industry |
| Adjunctive Glucocorticoid Therapy in Patients with Septic Shock | N Engl J Med | Venkatesh 2018 | 3800 | No | Therapeutic interventions | More than one | Yes | Yes | Yes - clinicaltrials.gov | Yes | Mortality | No | Non-industry |
| Adjunctive granisetron therapy in patients with sepsis or septic shock (GRANTISS): a single-center, single-blinded, randomized, controlled clinical trial | Frontiers in Pharmacology | Guan 2022 | 150 | Yes | Therapeutic interventions | Asia/Pacific | No | No | Yes - clinicaltrials.gov | Yes | Mortality | No | Non-industry |
| Adjunctive homeopathic treatment in patients with severe sepsis: a randomized, double-blind, placebo-controlled trial in an intensive care unit | Homeopathy | Frass 2005 | 67 | Yes | Therapeutic interventions | Europe | No | No | No | No | Mortality | Yes | Not reported |
| Adjunctive Sedation with Dexmedetomidine for the Prevention of Severe Inflammation and Septic Encephalopathy: A Pilot Randomized Controlled Study. | Crit Care Med | Iten 2025 | 70 | Yes | Other strategies | Europe | No | No | Yes - clinicaltrials.gov | Yes | Other | No | Non-industry |
| Adjunctive sepsis therapy with aminophylline (STAP): a randomized controlled trial | Chinese Medical Journal | Zhang 2022 | 100 | Yes | Therapeutic interventions | Asia/Pacific | No | Yes | Yes - Other | Yes | Mortality | Yes | Non-industry |
| Administration of amphotericin B in lipid emulsion decreases nephrotoxicity: results of a prospective, randomized, controlled study in critically ill patients. | Crit Care Med | Sorkine 1996 | 60 | No | Therapeutic interventions | Europe | No | No | No | No | Other | Yes | Not reported |
| Administration of higher doses of amikacin in early stages of sepsis in critically ill patients | Acta Med Iran | Najmeddin 2014 | 40 | No | Therapeutic interventions | Asia/Pacific | No | No | No | No | Other | No | Non-industry |
| Administration of low-dose dopamine to nonoliguric patients with sepsis syndrome does not raise intramucosal gastric pH nor improve creatinine clearance | Am J Respir Crit Care Med | Olson 1996 | 16 | Yes | Therapeutic interventions | North America | No | No | No | No | Other | No | Not reported |
| Administration of the nitric oxide synthase inhibitor NG-methyl-L-arginine hydrochloride (546C88) by intravenous infusion for up to 72 hours can promote the resolution of shock in patients with severe sepsis: results of a randomized, double-blind, placebo | Crit Care Med | Bakker 2004 | 312 | No | Therapeutic interventions | More than one | Yes | Yes | No | Yes | Other | Yes | Industry |
| Aerosolized prostacyclin and inhaled nitric oxide in septic shock--different effects on splanchnic oxygenation? | Intensive Care Med | Eichelbronner 1996 | 16 | No | Therapeutic interventions | Europe | Yes | Yes | No | No | Other | Yes | Non-industry |
| Albumin replacement in patients with severe sepsis or septic shock | N Engl J Med | Caironi 2014 | 1810 | Yes | Other strategies | Europe | No | Yes | Yes - clinicaltrials.gov | Yes | Mortality | No | Non-industry |
| Alkaline phosphatase for treatment of sepsis-induced acute kidney injury: a prospective randomized double-blind placebo-controlled trial | Crit Care | Pickkers 2012 | 36 | No | Therapeutic interventions | Europe | Yes | Yes | Yes - clinicaltrials.gov | Yes | Other | Yes | Industry |
| Alkaline phosphatase treatment improves renal function in severe sepsis or septic shock patients | Crit Care Med | Heemskerk 2009 | 15 | No | Therapeutic interventions | Europe | Yes | Yes | No | No | Other | Yes | Industry |
| Alteco endotoxin hemoadsorption in Gram-negative septic shock patients | Indian J Crit Care Med | Shum 2014 | 15 | No | Other strategies | Asia/Pacific | No | No | Yes - Other | Yes | Other | No | Non-industry |
| An Electronic Tool for the Evaluation and Treatment of Sepsis in the ICU: a Randomized Controlled Trial | Crit Care Med | Semler 2015 | 218 | No | Care bundles | North America | No | No | No | No | Other | No | Non-industry |
| An open-label, randomized controlled trial to assess a ketogenic diet in critically ill patients with sepsis. | Science translational medicine | Rahmel 2024 | 40 | Yes | Other strategies | Europe | No | No | Yes - Other | Yes | Other | Yes | Non-industry |
| An open-label, randomized, phase 3 study of the efficacy and safety of antithrombin gamma in patients with sepsis-induced disseminated intravascular coagulation syndrome | J Intensive Care | AntithrombinGammaStudyGrp 2018 | 222 | No | Therapeutic interventions | Asia/Pacific | No | Yes | Yes - clinicaltrials.gov | Yes | Other | Yes | Industry |
| Analysis on the effects of dexmedetomidine on ventilator free days and mortality in sepsis patients receiving mechanical ventilaiton | International Journal of Medicine and Public Health | Kushare 2024 | 100 | No | Therapeutic interventions | Asia/Pacific | No | No | No | No | Other | No | Non-industry |
| Antibiotic stewardship program in Intensive Care Unit: First report from Iran. | International Journal of Critical Illness and Injury Science | Vahidi 2018 | 100 | No | Other strategies | Asia/Pacific | No | No | Yes - Other | No | Other | No | Non-industry |
| Antithrombin III in patients with severe sepsis: a pharmacokinetic study | Intensive Care Med | Ilias 2000 | 33 | No | Therapeutic interventions | Europe | Yes | Yes | No | No | Other | No | Industry |
| Antithrombin III in patients with severe sepsis. A randomized, placeho-controlled, double-blind multicenter trial plus a meta-analysis on all randomized, placebo-controlled, double-blind trials with antithrombin III in severe sepsis | Intensive Care Med | Eisele 1998 | 122 | No | Therapeutic interventions | Europe | Yes | Yes | No | No | Mortality | No | Industry |
| Application of a combination of lactated Ringer's solution and ulinastatin for early resuscitation in sepsis | Tropical Journal of Pharmaceutical Research | Min 2023 | 82 | No | Care bundles | Asia/Pacific | No | No | No | No | Other | No | Non-industry |
| Application of ultrasound-guided external jugular vein puncture in intensive care unit (ICU) patients with severe sepsis: a randomised trial | Ann Palliat Med | Luo 2021 | 61 | No | Other strategies | Asia/Pacific | No | No | No | No | Other | Yes | Non-industry |
| Assessment of hemodynamic efficacy and safety of 6% hydroxyethylstarch 130/0.4 vs. 0.9% NaCl fluid replacement in patients with severe sepsis: the CRYSTMAS study | Crit Care | Guidet 2012 | 196 | No | Other strategies | Europe | Yes | Yes | Yes - clinicaltrials.gov | Yes | Other | Yes | Industry |
| Assessment of the effect of unfractionated heparin administered either by intravenous infusion vs. subcutaneous injection on heparin-binding protein, and plasminogen activator inhibitor-1 in critically ill septic patients: a randomized controlled trial. | European Review for Medical and Pharmacological Sciences | Kassem 2024 | 40 | Yes | Therapeutic interventions | Africa | No | No | Yes - clinicaltrials.gov | Yes | Other | No | Non-industry |
| Assessment of the safety and efficacy of the monoclonal anti-tumor necrosis factor antibody-fragment, MAK 195F, in patients with sepsis and septic shock: a multicenter, randomized, placebo-controlled, dose-ranging study | Crit Care Med | Reinhart 1996 | 122 | No | Therapeutic interventions | Europe | No | Yes | Yes - Other | No | Other | No | Industry |
| Assessment of the safety of recombinant tissue factor pathway inhibitor in patients with severe sepsis: a multicenter, randomized, placebo-controlled, single-blind, dose escalation study | Crit Care Med | Abraham 2001 | 210 | No | Therapeutic interventions | More than one | Yes | Yes | No | No | Other | No | Industry |
| Beneficial effects of short-term vasopressin infusion during severe septic shock | Anesthesiology | Patel 2002 | 13 | No | Therapeutic interventions | North America | No | No | No | No | Other | Yes | Non-industry |
| Beta-Lactam Infusion in Severe Sepsis (BLISS): a prospective, two-centre, open-labelled randomised controlled trial of continuous versus intermittent beta-lactam infusion in critically ill patients with severe sepsis | Intensive Care Med | Abdul-Aziz 2016 | 140 | No | Therapeutic interventions | Asia/Pacific | No | Yes | Yes - Other | No | Other | Yes | Non-industry |
| Biomarker-Guided Antibiotic Duration for Hospitalized Patients With Suspected Sepsis: The ADAPT-Sepsis Randomized Clinical Trial. | JAMA | Dark 2025 | 918 | No | Therapeutic interventions | Europe | No | Yes | Yes - Other | Yes | Other | Yes | Non-industry |
| C1-esterase inhibitor infusion increases survival rates for patients with sepsis* | Crit Care Med | Igonin 2012 | 41 | No | Therapeutic interventions | Europe | No | Yes | No | No | Mortality | Yes | Industry |
| C1-inhibitor in patients with severe sepsis and septic shock: beneficial effect on renal dysfunction | Crit Care Med | Caliezi 2002 | 40 | Yes | Therapeutic interventions | Europe | No | No | No | Yes | Other | Yes | Industry |
| Calcitriol in Sepsis-A Single-Centre Randomised Control Trial. | J Clin Med | Thampi 2024 | 152 | Yes | Therapeutic interventions | Asia/Pacific | No | No | Yes - Other | Yes | Other | No | Non-industry |
| Can the global end-diastolic volume index guide fluid management in septic patients? A multicenter randomized controlled trial | Acute Medicine & Surgery | Morisawa 2020 | 159 | Yes | Care bundles | Asia/Pacific | No | Yes | Yes - Other | Yes | Other | No | Not reported |
| Cardiovascular effects of the nitric oxide synthase inhibitor NG-methyl-L-arginine hydrochloride (546C88) in patients with septic shock: results of a randomized, double-blind, placebo-controlled multicenter study (study no. 144-002) | Crit Care Med | Watson 2004 | 312 | No | Therapeutic interventions | More than one | Yes | Yes | No | No | Other | Yes | Industry |
| CD14 receptor occupancy in severe sepsis: results of a phase I clinical trial with a recombinant chimeric CD14 monoclonal antibody (IC14) | Crit Care Med | Reinhart 2004 | 40 | No | Therapeutic interventions | Europe | Yes | Yes | No | No | Other | Yes | Industry |
| CDP571, a humanized antibody to human tumor necrosis factor-alpha: Safety, pharmacokinetics, immune response, and influence of the antibody on cytokine concentrations in patients with septic shock | Crit Care Med | Dhainaut 1995 | 42 | No | Therapeutic interventions | Europe | Yes | Yes | No | No | Mortality | No | Industry |
| Cerebral autoregulation-directed optimal blood pressure management reduced the risk of delirium in patients with septic shock | J Intensive Care Med | Peng 2024 | 51 | Yes | Monitoring systems | Asia/Pacific | No | No | Yes - clinicaltrials.gov | No | Other | Yes | Non-industry |
| Ciprofol versus propofol for long-term sedation in mechanically ventilated patients with sepsis: a randomized controlled trial. | BMC Anesthesiol | Zhao 2025 | 60 | Yes | Other strategies | Asia/Pacific | No | No | Yes - Other | Yes | Other | No | Non-industry |
| Circulating protein carbonyls are specifically elevated in critically ill patients with pneumonia relative to other sources of sepsis | Free Radic Biol Med | Spencer 2022 | 40 | No | Therapeutic interventions | Asia/Pacific | No | No | Yes - Other | Yes | Other | No | Non-industry |
| Clearance of vancomycin during high-volume haemofiltration: Impact of pre-dilution | Intensive Care Med | Uchino 2002 | 7 | No | Therapeutic interventions | Asia/Pacific | No | No | No | No | Other | Yes | Not reported |
| Clinical and biochemical endpoints and predictors of response to plasma exchange in septic shock: results from a randomized controlled trial | Crit Care | Stahl 2022 | 40 | Yes | Monitoring systems | Europe | No | Yes | Yes - clinicaltrials.gov | Yes | Other | Yes | Non-industry |
| Clinical and economic impact of procalcitonin to shorten antimicrobial therapy in septic patients with proven bacterial infection in an intensive care setting | Diagnostic Microbiology and Infectious Disease | Deliberato 2013 | 81 | Yes | Monitoring systems | Latin America | No | No | Yes - clinicaltrials.gov | Yes | Other | Yes | Not reported |
| Clinical and metabolic effects of two lipid emulsions on the parenteral nutrition of septic patients | Nutrition | Garnacho-Montero 2002 | 26 | No | Other strategies | Europe | No | No | No | No | Other | Yes | Not reported |
| Clinical Assessment of Continuous Hemodialysis with the Medium Cutoff EMiC√Ç¬Æ2 Membrane in Patients with Septic Shock | Blood Purif | Ferrari 2022 | 20 | Yes | Other strategies | Europe | No | No | Yes - clinicaltrials.gov | Yes | Other | Yes | Industry |
| Clinical effect of combined ulinastatin and continuous renal replacement therapy on management of severe sepsis with acute kidney injury | Tropical Journal of Pharmaceutical Research | Fang 2017 | 106 | No | Care bundles | Asia/Pacific | No | No | No | No | Other | Yes | Not reported |
| Clinical efficacy of continuous infusion of piperacillin compared with intermittent dosing in septic critically ill patients | Int J Antimicrob Agents | Rafati 2006 | 20 | No | Therapeutic interventions | Asia/Pacific | No | No | No | No | Other | Yes | Non-industry |
| Clinical efficacy of ulinastatin in the treatment of unliquefied pyogenic liver abscess complicated by septic shock: a randomized controlled trial | Immun Inflamm Dis | Guo 2023 | 99 | No | Therapeutic interventions | Asia/Pacific | No | No | Yes - Other | Yes | Other | Yes | Not reported |
| Clinical outcome study of critically-ill septic patients given taurine supplemented enteral nutrition | N Engl J Med | Elmokadem 2015 | 45 | No | Other strategies | Africa | No | No | No | No | Other | Yes | Not reported |
| Clinical outcomes of empirical high-dose meropenem in critically ill patients with sepsis and septic shock: a randomized controlled trial | J Intensive Care | Lertwattanachai 2020 | 76 | Yes | Therapeutic interventions | Asia/Pacific | No | No | Yes - clinicaltrials.gov | Yes | Other | No | Non-industry |
| Clinical Trial Assessment of Intermittent and Continuous Infusion Dose of N-Acetylcysteine on Redox Status of the Body in Patients with Sepsis Admitted to the ICU | J Intensive Care Med | PeivandiYazdi 2020 | 20 | No | Therapeutic interventions | Asia/Pacific | No | No | Yes - Other | Yes | Other | Yes | Non-industry |
| Co-administration of iloprost and eptifibatide in septic shock (CO-ILEPSS)-a randomised, controlled, double-blind investigator-initiated trial investigating safety and efficacy | Crit Care | Berthelsen 2019 | 24 | No | Therapeutic interventions | Europe | No | No | Yes - clinicaltrials.gov | Yes | Other | No | Non-industry |
| Combination therapy of vitamin c and thiamine on matrix metalloproteinas-es-9 (Mmp-9) for septic in icu | Journal of Drug and Alcohol Research | Lubis 2021 |  | No | Therapeutic interventions | Asia/Pacific | No | Yes | No | No | Other | Yes | Not reported |
| Combination therapy with milrinone and esmolol for heart protection in patients with severe sepsis: a prospective, randomized trial | Clin Drug Investig | Wang 2015 | 90 | No | Therapeutic interventions | Asia/Pacific | No | No | No | No | Other | Yes | Not reported |
| Combined Treatment With Hydrocortisone, Vitamin C, and Thiamine for Sepsis and Septic Shock: a Randomized Controlled Trial | Chest | Chang 2020 | 80 | Yes | Therapeutic interventions | Asia/Pacific | No | No | Yes - clinicaltrials.gov | Yes | Mortality | No | Non-industry |
| Comparable Effect of Two-Step Versus Extended Infusions on the Pharmacokinetics of Imipenem in Patients with Sepsis and Septic Shock | Adv Ther | Huang 2020 | 35 | No | Therapeutic interventions | Asia/Pacific | No | No | Yes - clinicaltrials.gov | Yes | Other | No | Non-industry |
| Comparative efficacy and prognostic impact of continuous versus intermittent hydrocortisone administration in septic shock patients. | Scientific reports | Jin 2025 | 60 | No | Therapeutic interventions | Asia/Pacific | No | Yes | No | No | Mortality | Yes | Non-industry |
| Comparative evaluation of central venous pressure and sonographic inferior vena cava variability in assessing fluid responsiveness in septic shock | Indian J Crit Care Med | Garg 2016 | 31 | No | Monitoring systems | Asia/Pacific | No | No | No | No | Other | Yes | Non-industry |
| Comparative study between high and low dose methylene blue infusion in septic cancer patients: a randomized, blinded, controlled study. | BMC Anesthesiol | Shaker 2025 | 90 | Yes | Therapeutic interventions | Africa | No | No | Yes - clinicaltrials.gov | Yes | Other | Yes | Non-industry |
| Comparison Between Continuous and Intermittent Administration of Hydrocortisone During Septic Shock: a Randomized Controlled Clinical Trial | Shock | Tilouche 2019 | 70 | Yes | Therapeutic interventions | Asia/Pacific | No | No | No | No | Other | Yes | Not reported |
| Comparison Between Norepinephrine Alone Versus Norepinephrine/Vasopressin Combination for Resuscitation in Septic Shock A Randomized Clinical Trial | The Egyptian Journal of Critical Care Medicine | Hussien 2021 | 90 | Yes | Therapeutic interventions | Africa | No | No | Yes - clinicaltrials.gov | Yes | Other | Yes | Not reported |
| Comparison of 6% hydroxyethyl starch 130/0.4 and saline solution for resuscitation of the microcirculation during the early goal-directed therapy of septic patients | J Crit Care | Dubin 2010 | 11 | No | Other strategies | Latin America | No | Yes | Yes - clinicaltrials.gov | Yes | Other | Yes | Non-industry |
| Comparison of dexmedetomidine and propofol in mechanically ventilated patients with sepsis: A pilot study | The Southwest Respiratory and Critical Care Chronicles | MarkSigler 2018 | 28 | No | Therapeutic interventions | North America | No | No | No | No | Other | No | Not reported |
| Comparison of intermittent and bolus enteral feeding methods on enteral feeding intolerance of patients with sepsis: a triple-blind controlled trial in intensive care units | Middle East J Dig Dis | Nasiri 2017 | 60 | No | Other strategies | Asia/Pacific | No | No | Yes - Other | Yes | Other | No | Non-industry |
| Comparison of norepinephrine and dobutamine to epinephrine for hemodynamics, lactate metabolism, and gastric tonometric variables in septic shock: a prospective, randomized study | Intensive Care Med | Levy 1997 | 30 | No | Therapeutic interventions | Europe | No | No | No | No | Other | Yes | Non-industry |
| Comparison of norepinephrine and dopamine in the management of septic shock using impedance cardiography | Indian J Crit Care Med | Mathur 2007 | 50 | No | Therapeutic interventions | Asia/Pacific | No | No | No | No | Other | Yes | Not reported |
| Comparison of Norepinephrine and Terlipressin vs Norepinephrine Alone for Management of Septic Shock: a Randomized Control Study | Indian J Crit Care Med | Sahoo 2022 | 50 | No | Therapeutic interventions | Asia/Pacific | No | No | Yes - Other | Yes | Other | Yes | Non-industry |
| Comparison of phenylephrine and norepinephrine in the management of dopamine-resistant septic shock | Indian J Crit Care Med | Jain 2010 | 27 | No | Therapeutic interventions | Asia/Pacific | No | No | No | No | Other | No | Not reported |
| Comparison of systemic and regional effects of dobutamine and dopexamine in norepinephrine-treated septic shock | Intensive Care Med | Levy 1999 | 24 | No | Therapeutic interventions | Europe | No | No | No | No | Other | No | Industry |
| Comparison of systemic and renal effects of dopexamine and dopamine in norepinephrine-treated septic shock | J Cardiothorac Vasc Anesth | Schmoelz 2006 | 28 | No | Therapeutic interventions | Europe | No | No | No | No | Other | No | Industry |
| Comparison of the cytokine adsorption ability in continuous renal replacement therapy using polyethyleneimine-coated polyacrylonitrile (AN69ST) or polymethylmethacrylate (PMMA) hemofilters: a pilot single-center open-label randomized control trial. | European Journal of Medical Research | Nakamura 2023 | 53 | Yes | Other strategies | Asia/Pacific | No | No | Yes - Other | Yes | Other | Yes | Non-industry |
| Comparison of the eff ects of intravenous, enteral and enteral + intravenous supply of glutamine on malnutrition in sepsis | Asia Pac J Clin Nutr | Koksal 2011 | 10 | No | Other strategies | Europe | No | No | No | No | Other | Yes | Not reported |
| Comparison of the effects of subcutaneous versus continuous infusion of heparin on key inflammatory parameters following sepsis | Anesth Pain Med | Nouri 2016 | 30 | Yes | Therapeutic interventions | Asia/Pacific | No | No | Yes - Other | Yes | Other | Yes | Non-industry |
| Comparison of the effects of vitamin C and thiamine on refractory hypotension in patients with sepsis: A randomized controlled trial | International Journal of Critical Illness and Injury Science | Nandhini 2022 | 84 | Yes | Therapeutic interventions | Asia/Pacific | No | No | Yes - Other | Yes | Mortality | Yes | Not reported |
| Comparison of vasopressin and phenylephrine in treatment of dopamine resistant septic shock - A randomised control trial | Asian Journal of Pharmaceutical and Clinical Research | Patro 2021 | 50 | No | Therapeutic interventions | Asia/Pacific | No | No | No | No | Other | Yes | Non-industry |
| Confirmatory interleukin-1 receptor antagonist trial in severe sepsis: a phase III, randomized, double-blind, placebo-controlled, multicenter trial. The Interleukin-1 Receptor Antagonist Sepsis Investigator Group | Crit Care Med | Opal 1997 | 696 | No | Therapeutic interventions | More than one | Yes | Yes | No | No | Mortality | No | Industry |
| Confirmatory platelet-activating factor receptor antagonist trial in patients with severe Gram-negative bacterial sepsis: A phase III, randomized, double-blind, placebo-controlled, multicenter trial | Crit Care Med | Dhainaut 1998 | 609 | No | Therapeutic interventions | Europe | Yes | Yes | No | No | Other | No | Industry |
| Conservative Fluid Management After Sepsis Resuscitation: a Pilot Randomized Trial | J Intensive Care Med | Semler 2020 | 30 | Yes | Other strategies | North America | No | No | Yes - clinicaltrials.gov | Yes | Other | No | Non-industry |
| Continuous infusion of beta-lactam antibiotics in severe sepsis: a multicenter double-blind, randomized controlled trial | Clin Infect Dis | Dulhunty 2013 | 30 | No | Therapeutic interventions | Asia/Pacific | Yes | Yes | Yes - Other | Yes | Other | Yes | Non-industry |
| Continuous renal replacement therapy in sepsis-associated acute kidney injury: effects on inflammatory mediators and coagulation function | Asian J Surg | Wu 2021 | 90 | Yes | Other strategies | Asia/Pacific | No | No | No | No | Other | Yes | Not reported |
| Continuous terlipressin versus vasopressin infusion in septic shock (TERLIVAP): a randomized, controlled pilot study | Crit Care | Morelli 2009 | 45 | No | Therapeutic interventions | Europe | Yes | Yes | Yes - clinicaltrials.gov | Yes | Other | Yes | Non-industry |
| Continuous veno-venous hemofiltration for septic shock | Int J Clin Exp Med | Hui 2017 | 70 | Yes | Other strategies | Asia/Pacific | No | No | No | No | Other | Yes | Not reported |
| Continuous versus intermittent bolus dosing of beta-lactam antibiotics in a South African multi-disciplinary intensive care unit: A randomized controlled trial. | The Journal of infection | Khan 2025 | 122 | No | Therapeutic interventions | Africa | No | No | Yes - Other | Yes | Other | No | Not reported |
| Continuous vs Intermittent beta-Lactam Antibiotic Infusions in Critically Ill Patients With Sepsis: The BLING III Randomized Clinical Trial. | JAMA | Dulhunty 2024 | 3533 | No | Therapeutic interventions | More than one | Yes | Yes | Yes - clinicaltrials.gov | Yes | Mortality | No | Non-industry |
| Continuous vs Intermittent Meropenem Administration in Critically Ill Patients With Sepsis: the MERCY Randomized Clinical Trial | JAMA | Monti 2023 | 607 | Yes | Therapeutic interventions | More than one | Yes | Yes | Yes - clinicaltrials.gov | Yes | Other | No | Non-industry |
| Corticosteroid treatment and intensive insulin therapy for septic shock in adults: a randomized controlled trial | JAMA | Annane 2010 | 255 | No | Therapeutic interventions | Europe | No | Yes | Yes - clinicaltrials.gov | Yes | Mortality | No | Non-industry |
| Cost-Effectiveness of de novo Simvastatin as Adjunctive Therapy in Patients Critically Ill with Sepsis | American Health & Drug Benefits | MostafaEladawy 2022 | 80 | No | Therapeutic interventions | Africa | No | Yes | Yes - clinicaltrials.gov | Yes | Other | Yes | Not reported |
| Coupled plasma filtration adsorption (CPFA) plus Continuous Veno-Venous Haemofiltration (CVVH) versus CVVH alone as an adjunctive therapy in the treatment of sepsis | EXCLI Journal | Hassan 2013 | 23 | Yes | Other strategies | Asia/Pacific | No | No | No | No | Other | Yes | Non-industry |
| Critical Care Ultrasound Goal-directed Versus Early Goal-directed Therapy in Septic Shock: a Randomized Controlled Study | Intensive Care Med | Zhang 2021 | 86 | Yes | Care bundles | Asia/Pacific | No | No | Yes - Other | Yes | Other | Yes | Non-industry |
| Critically ill septic patients have elevated oxidative stress biomarkers: lack of attenuation by parenteral vitamin C | Nutr Res | Vlasiuk 2022 | 50 | No | Therapeutic interventions | Asia/Pacific | No | No | Yes - Other | Yes | Other | No | Non-industry |
| De-escalation versus continuation of empirical antimicrobial treatment in severe sepsis: a multicenter non-blinded randomized noninferiority trial | Intensive Care Med | Leone 2014 | 116 | Yes | Other strategies | Europe | No | Yes | Yes - clinicaltrials.gov | Yes | Other | No | Non-industry |
| Determination of end point of fluid resuscitation using simplified lung ultrasound protocol in patients with septic shock | The Egyptian Journal of Chest Diseases and Tuberculosis | Ismail 2019 | 80 | No | Care bundles | Africa | No | No | No | No | Other | Yes | Not reported |
| Dexmedetomidine for Reducing Mortality in Patients With Septic Shock: A Randomized Controlled Trial (DecatSepsis). | Chest | EzzAl-Regal 2024 | 90 | Yes | Therapeutic interventions | Africa | No | No | Yes - clinicaltrials.gov | Yes | Mortality | No | Non-industry |
| Dexmedetomidine or Propofol for Sedation in Mechanically Ventilated Adults with Sepsis | N Engl J Med | Hughes 2021 | 432 | No | Therapeutic interventions | North America | No | Yes | Yes - clinicaltrials.gov | Yes | Other | No | Non-industry |
| Dexmedetomidine protects against acute kidney injury in patients with septic shock | Ann Palliat Med | Liu 2020 |  | No | Therapeutic interventions | Asia/Pacific | No | No | No | No | Other | Yes | Non-industry |
| Dexmedetomidine to Reduce Vasopressor Resistance in Refractory Septic Shock: alpha2 Agonist Dexmedetomidine for REfractory Septic Shock (ADRESS): A Double-Blind Randomized Controlled Pilot Trial. | Crit Care Med | Dargent 2025 | 32 | Yes | Therapeutic interventions | Europe | No | Yes | Yes - clinicaltrials.gov | Yes | Other | No | Non-industry |
| Dexmedetomidine versus propofol or midazolam in patients with abdominal sepsis regarding inflammatory response and capillary leak | Egyptian Journal of Anaesthesia | Moeen 2022 | 60 | No | Therapeutic interventions | Africa | No | No | Yes - clinicaltrials.gov | Yes | Other | Yes | Non-industry |
| Dextrose 5% versus normal saline as maintenance fluid therapy in patients with septic shock (DEMANDS): a randomized controlled trial | Alexandria Journal of Medicine | Fayed 2024 | 50 | No | Other strategies | Africa | No | No | Yes - Other | Yes | Other | Yes | Not reported |
| Dialysis complications in acute kidney injury patients treated with prolonged intermittent renal replacement therapy sessions lasting 10 versus 6 hours: results of a randomized clinical trial | Artificial Organs | Albino 2015 | 75 | Yes | Other strategies | Latin America | No | No | Yes - Other | No | Other | No | Not reported |
| Distribution of normal saline and 5% albumin infusions in septic patients | Crit Care Med | Ernest 1999 | 18 | No | Other strategies | Europe | No | No | Yes - clinicaltrials.gov | Yes | Other | Yes | Not reported |
| Dobutamine and gastric-to-arterial carbon dioxide gap in severe sepsis without shock | Intensive Care Med | Lebuffe 2002 | 25 | No | Therapeutic interventions | Europe | Yes | Yes | No | No | Other | No | Industry |
| Does Maintaining a Targeted Abdominal Perfusion Pressure Reduce Renal Damage in Patients with Septic Shock?: a Randomized, Controlled, and Open-label Study | Balkan Med J | Ozkarakas 2023 | 72 | Yes | Monitoring systems | Europe | No | No | Yes - clinicaltrials.gov | Yes | Other | Yes | Not reported |
| Does N-acetyl-L-cysteine influence cytokine response during early human septic shock? | Chest | Spapen 1998 | 12 | No | Therapeutic interventions | Europe | No | No | No | No | Other | No | Not reported |
| Does nano-curcumin supplementation improve hematological indices in critically ill patients with sepsis? A randomized controlled clinical trial | J Food Biochem | Naeini 2022 | 14 | No | Therapeutic interventions | Asia/Pacific | No | No | Yes - Other | Yes | Other | Yes | Non-industry |
| Dopexamine and norepinephrine versus epinephrine on gastric perfusion in patients with septic shock: a randomized study [NCT00134212]. | Crit Care | Seguin 2006 | 22 | No | Therapeutic interventions | Europe | No | No | Yes - clinicaltrials.gov | Yes | Other | Yes | Non-industry |
| Drotrecogin alfa (activated) for adults with severe sepsis and a low risk of death | N Engl J Med | Abraham 2005 | 2640 | No | Therapeutic interventions | More than one | Yes | Yes | No | Yes | Mortality | No | Industry |
| Drotrecogin Alfa (Activated) in Adults with Septic Shock | N Engl J Med | Ranieri 2012 | 1697 | Yes | Therapeutic interventions | More than one | Yes | Yes | Yes - clinicaltrials.gov | Yes | Mortality | No | Industry |
| Dyslipidemia: a prospective controlled randomized trial of intensive glycemic control in sepsis | Intensive Care Med | Cappi 2012 | 63 | Yes | Other strategies | Latin America | No | No | Yes - clinicaltrials.gov | Yes | Other | Yes | Non-industry |
| E5 murine monoclonal antiendotoxin antibody in gram-negative sepsis: A randomized controlled trial | JAMA | Angus 2000 | 1090 | No | Therapeutic interventions | North America | No | Yes | No | Yes | Mortality | No | Industry |
| Early adjunctive methylene blue in patients with septic shock: a randomized controlled trial | Crit Care | Ibarra-Estrada 2023 | 91 | Yes | Therapeutic interventions | Latin America | No | No | Yes - clinicaltrials.gov | Yes | Other | Yes | Not reported |
| Early administration of hydrocortisone, vitamin C, and thiamine in adult patients with septic shock: a randomized controlled clinical trial | Crit Care | Lyu 2022 | 426 | Yes | Therapeutic interventions | Asia/Pacific | No | No | Yes - clinicaltrials.gov | Yes | Mortality | No | Non-industry |
| Early dexamethasone treatment for septic shock patients: a prospective randomized clinical trial | Sao Paulo Med J | Cicarelli 2007 | 29 | Yes | Therapeutic interventions | Latin America | No | No | No | No | Mortality | Yes | Not reported |
| Early enteral supplementation with key pharmaconutrients improves Sequential Organ Failure Assessment score in critically ill patients with sepsis: outcome of a randomized, controlled, double-blind trial | Crit Care Med | Beale 2008 | 55 | No | Other strategies | Europe | No | No | No | No | Other | Yes | Industry |
| Early high-dose continuous veno-venous hemofiltration alleviates the alterations of CD4+ T lymphocyte subsets in septic patients combined with acute kidney injury | Artif Organs | Jin 2022 | 53 | No | Other strategies | Asia/Pacific | No | Yes | Yes - Other | Yes | Other | Yes | Non-industry |
| Early Initiation of Low-Dose Hydrocortisone Therapy for Septic Shock in Geriatric Patients: a Randomized Control Trial | Journal of The Association of Physicians of India | Agarwal 2022 | 120 | Yes | Therapeutic interventions | Asia/Pacific | No | No | No | No | Mortality | No | Not reported |
| Early initiation of low-dose hydrocortisone treatment for septic shock in adults: a randomized clinical trial | Am J Emerg Med | Lv 2017 | 120 | Yes | Therapeutic interventions | Asia/Pacific | No | No | Yes - clinicaltrials.gov | Yes | Mortality | No | Not reported |
| Early Lactate-Guided Resuscitation of Elderly Septic Patients | J Intensive Care Med | Chen 2022 | 82 | Yes | Care bundles | Asia/Pacific | No | No | Yes - clinicaltrials.gov | No | Mortality | No | Not reported |
| Early low-dose glucocorticoid therapy effectively suppresses serum pro-inflammatory factors such as IL-6 and inhibits apoptosis of CD4+ cells in septic shock patients | Int J Clin Exp Med | Jiang 2019 | 60 | Yes | Therapeutic interventions | Asia/Pacific | No | No | No | No | Other | Yes | Non-industry |
| Early peripheral perfusion targeted fluid therapy leads to less fluid administration in patients with septic shock: a prospective randomized controlled trial | Am J Respir Crit Care Med | VanGenderen 2014 | 15 | No | Care bundles | Europe | No | No | Yes - clinicaltrials.gov | Yes | Other | No | Not reported |
| Early physical rehabilitation in intensive care patients with sepsis syndromes: a pilot randomised controlled trial | Intensive Care Med | Kayambu 2015 | 50 | No | Other strategies | Asia/Pacific | No | No | Yes - Other | Yes | Other | Yes | Non-industry |
| Early use of polymyxin B hemoperfusion in abdominal septic shock: the EUPHAS randomized controlled trial | JAMA | Cruz 2009 | 34 | No | Other strategies | Europe | No | Yes | Yes - clinicaltrials.gov | Yes | Other | Yes | Non-industry |
| Early use of polymyxin B hemoperfusion in patients with septic shock due to peritonitis: a multicenter randomized control trial | Intensive Care Med | Payen 2015 | 243 | Yes | Other strategies | Europe | No | Yes | Yes - clinicaltrials.gov | Yes | Mortality | No | Industry |
| Echocardiogram-guided resuscitation versus early goal-directed therapy in the treatment of septic shock: a randomized, controlled, feasibility trial | J Intensive Care | Lanspa 2018 | 30 | No | Care bundles | North America | No | No | Yes - clinicaltrials.gov | Yes | Other | No | Non-industry |
| Echocardiography-guided hemodynamic management of severe sepsis and septic shock in adults: a randomized controlled trial | Anaesth pain intensive care | Alhabashy 2021 | 87 | No | Monitoring systems | Africa | No | No | Yes - Other | No | Mortality | Yes | Non-industry |
| Effect of a chimeric antibody to tumor necrosis factor-alpha on cytokine and physiologic responses in patients with severe sepsis - A randomized, clinical trial | Crit Care Med | Clark 1998 | 56 | No | Therapeutic interventions | More than one | Yes | Yes | No | No | Mortality | No | Industry |
| Effect of a multifaceted educational intervention for anti-infectious measures on sepsis mortality: a cluster randomized trial | Intensive Care Med | Bloos 2017 | 4183 | No | Other strategies | Europe | No | Yes | Yes - clinicaltrials.gov | Yes | Mortality | No | Non-industry |
| Effect of A Probiotic Preparation on Gut Microbiota in Critically Ill Septic Patients Admitted to Intensive Care Unit: a Pilot Randomized Controlled Trial | Pharmaceutical Sciences | Mahmoodpoor 2023 | 40 | No | Therapeutic interventions | Asia/Pacific | No | Yes | Yes - Other | Yes | Other | Yes | Not reported |
| Effect of a Recombinant Human Soluble Thrombomodulin on Mortality in Patients With Sepsis-Associated Coagulopathy: the SCARLET Randomized Clinical Trial | JAMA | Vincent 2019 | 405 | No | Therapeutic interventions | More than one | Yes | Yes | Yes - clinicaltrials.gov | Yes | Mortality | No | Industry |
| Effect of a Resuscitation Strategy Targeting Peripheral Perfusion Status vs Serum Lactate Levels on 28-Day Mortality Among Patients With Septic Shock: the ANDROMEDA-SHOCK Randomized Clinical Trial | Am J Respir Crit Care Med | Hernandez 2019 | 424 | No | Other strategies | Latin America | Yes | Yes | Yes - clinicaltrials.gov | Yes | Mortality | No | Non-industry |
| Effect of an enteral diet enriched with eicosapentaenoic acid, gamma-linolenic acid and anti-oxidants on the outcome of mechanically ventilated, critically ill, septic patients | Clin Nutr | Grau-Carmona 2011 | 198 | No | Other strategies | Europe | No | Yes | Yes - clinicaltrials.gov | Yes | Other | No | Industry |
| Effect of an Herbal-Based Injection on 28-Day Mortality in Patients With Sepsis: The EXIT-SEP Randomized Clinical Trial. | JAMA | Liu 2023 | 1817 | Yes | Therapeutic interventions | Asia/Pacific | No | Yes | Yes - clinicaltrials.gov | Yes | Mortality | Yes | Non-industry |
| Effect of antithrombin III supplementation on inflammatory response in patients with severe sepsis | Shock | Inthorn 1998 | 29 | Yes | Therapeutic interventions | Europe | No | No | No | No | Other | Yes | Not reported |
| Effect of Ascorbic Acid, Corticosteroids, and Thiamine on Organ Injury in Septic Shock: The ACTS Randomized Clinical Trial | JAMA | Moskowitz 2020 | 205 | Yes | Therapeutic interventions | North America | No | Yes | Yes - clinicaltrials.gov | Yes | Other | No | Non-industry |
| Effect of Cholecalciferol Supplementation on Vitamin D Status and Cathelicidin Levels in Sepsis: a Randomized, Placebo-Controlled Trial | Crit Care Med | Quraishi 2015 | 10 | No | Therapeutic interventions | North America | No | No | Yes - clinicaltrials.gov | Yes | Other | Yes | Non-industry |
| Effect of continuous blood purification combined with reduced glutathione on endotoxin, inflammatory mediators and severity of liver injury in patients with septic shock | Tropical Journal of Pharmaceutical Research | Liu 2024 | 50 | No | Other strategies | Asia/Pacific | No | No | No | No | Other | Yes | Not reported |
| Effect of Continuous Infusion vs Bolus Dose of Hydrocortisone in Septic Shock: A Prospective Randomized Study. | Indian J Crit Care Med | Salhotra 2024 | 40 | Yes | Therapeutic interventions | Asia/Pacific | No | No | Yes - Other | Yes | Other | No | Non-industry |
| Effect of continuous renal replacement therapy on kidney injury molecule-1 and neutrophil gelatinase-associated lipocalin in patients with septic acute kidney injury | Exp Ther Med | Shao 2017 | 38 | No | Other strategies | Asia/Pacific | No | No | No | No | Other | Yes | Non-industry |
| Effect of CRRT with oXiris filter on hemodynamic instability in surgical septic shock with AKI: a pilot randomized controlled trial | Int J Artif Organs | Feng 2022 | 16 | No | Other strategies | Asia/Pacific | No | No | Yes - Other | No | Other | Yes | Non-industry |
| Effect of Dexmedetomidine on Mortality and Ventilator-Free Days in Patients Requiring Mechanical Ventilation With Sepsis: a Randomized Clinical Trial | JAMA | Kawazoe 2017 | 201 | Yes | Other strategies | Asia/Pacific | No | Yes | Yes - clinicaltrials.gov | Yes | Mortality | No | Industry |
| Effect of dexmedetomidine vs midazolam on the microcirculation of septic patients who are mechanically ventilated | Egyptian Journal of Anaesthesia | MohamedAtefRefaat 2022 | 128 | No | Therapeutic interventions | Africa | No | No | Yes - Other | Yes | Other | No | Not reported |
| Effect of Early Administration of Vitamin D on Clinical Outcome in Critically Ill Sepsis Patients: a Randomized Placebo-controlled Trial | Indian J Crit Care Med | Bhattacharyya 2021 | 126 | No | Therapeutic interventions | Asia/Pacific | No | No | Yes - Other | No | Other | No | Non-industry |
| Effect of empirical treatment with moxifloxacin and meropenem vs meropenem on sepsis-related organ dysfunction in patients with severe sepsis: a randomized trial | JAMA | Brunkhorst 2012 | 551 | Yes | Therapeutic interventions | Europe | No | Yes | Yes - clinicaltrials.gov | Yes | Other | No | Industry |
| Effect of enteral diet enriched with eicosapentaenoic acid, gamma-linolenic acid, and antioxidants in patients with sepsis-induced acute respiratory distress syndrome | J Intensive Care | Shirai 2015 | 46 | Yes | Other strategies | Asia/Pacific | No | No | No | No | Other | No | Not reported |
| Effect of eritoran, an antagonist of MD2-TLR4, on mortality in patients with severe sepsis: the ACCESS randomized trial | JAMA | Opal 2013 | 1961 | No | Therapeutic interventions | More than one | Yes | Yes | Yes - clinicaltrials.gov | Yes | Mortality | No | Industry |
| Effect of focused cardiopulmonary ultrasonography on clinical outcome of septic shock: a randomized study | Journal of International Medical Research | Li 2021 | 94 | Yes | Other strategies | Asia/Pacific | No | No | Yes - clinicaltrials.gov | Yes | Mortality | No | Non-industry |
| Effect of Ganciclovir on IL-6 Levels Among Cytomegalovirus-Seropositive Adults With Critical Illness: a Randomized Clinical Trial | JAMA | Limaye 2017 | 160 | No | Therapeutic interventions | North America | No | Yes | Yes - clinicaltrials.gov | Yes | Other | No | Non-industry |
| Effect of glucose-insulin-potassium infusion on hemodynamics in patients with septic shock | Open Access Macedonian Journal of Medical Sciences | Effat 2021 | 75 | No | Therapeutic interventions | Africa | No | No | No | No | Other | No | Non-industry |
| Effect of granulocyte-monocyte colony-stimulating factor therapy on leukocyte function and clearance of serious infection in nonneutropenic patients | Chest | Rosenbloom 2005 | 40 | No | Therapeutic interventions | North America | No | No | No | No | Other | Yes | Industry |
| Effect of heart rate control with esmolol on hemodynamic and clinical outcomes in patients with septic shock: a randomized clinical trial | JAMA | Morelli 2013 | 154 | Yes | Therapeutic interventions | Europe | No | No | Yes - clinicaltrials.gov | Yes | Other | Yes | Non-industry |
| Effect of high-dose Ascorbic acid on vasopressor's requirement in septic shock. | Journal of Research in Pharmacy Practice | Zabet 2016 | 28 | Yes | Therapeutic interventions | Asia/Pacific | No | No | No | No | Other | Yes | Non-industry |
| Effect of high-dose intravenous ascorbic acid on microcirculation and endothelial glycocalyx during sepsis and septic shock: a double-blind, randomized, placebo-controlled study | BMC Anesthesiology | Belousoviene 2023 | 23 | No | Therapeutic interventions | Europe | No | No | Yes - clinicaltrials.gov | No | Other | Yes | Industry |
| Effect of Human Recombinant Alkaline Phosphatase on 7-Day Creatinine Clearance in Patients With Sepsis-Associated Acute Kidney Injury: a Randomized Clinical Trial | JAMA | Pickkers 2018 | 301 | No | Therapeutic interventions | More than one | Yes | Yes | Yes - clinicaltrials.gov | Yes | Other | No | Industry |
| Effect of intravenous clarithromycin in patients with sepsis, respiratory and multiple organ dysfunction syndrome: a randomized clinical trial | Crit Care | Karakike 2022 | 110 | No | Therapeutic interventions | Europe | Yes | Yes | Yes - clinicaltrials.gov | Yes | Mortality | No | Non-industry |
| Effect of long-term and high-dose antithrombin supplementation on coagulation and fibrinolysis in patients with severe sepsis | Crit Care Med | Hoffmann 2004 | 20 | No | Therapeutic interventions | Europe | No | No | No | No | Other | Yes | Not reported |
| Effect of magnesium supplementation on lactate clearance in critically ill patients with severe sepsis: a randomized clinical trial | European Journal of Clinical Pharmacology | Noormandi 2020 | 58 | Yes | Therapeutic interventions | Asia/Pacific | No | No | Yes - Other | Yes | Other | Yes | Non-industry |
| Effect of mode of hydrocortisone administration on glycemic control in patients with septic shock: a prospective randomized trial | Crit Care | Loisa 2007 | 48 | No | Therapeutic interventions | Europe | No | Yes | Yes - Other | Yes | Other | Yes | Non-industry |
| Effect of n-3 fatty acids on markers of brain injury and incidence of sepsis-associated delirium in septic patients | Acta Anaesthesiol Scand | Burkhart 2014 | 50 | Yes | Other strategies | Europe | No | No | Yes - clinicaltrials.gov | Yes | Other | No | Industry |
| Effect of Neutrophil Elastase Inhibitor (Sivelestat Sodium) on Oxygenation in Patients with Sepsis-Induced Acute Respiratory Distress Syndrome. | Journal of Inflammation Research | Wu 2025 | 70 | Yes | Therapeutic interventions | Asia/Pacific | No | Yes | Yes - Other | Yes | Other | Yes | Industry |
| Effect of pentoxifylline in severe sepsis: results of a randomized, double-blind, placebo-controlled study | Arch Surg | Staubach 1998 | 51 | Yes | Therapeutic interventions | Europe | No | Yes | No | No | Other | Yes | Industry |
| Effect of pentoxifylline on organ dysfunction and mortality in severe sepsis | Open Anesthesia J | Elgendy 2020 | 52 | No | Therapeutic interventions | Africa | No | Yes | No | No | Other | No | Not reported |
| Effect of physostigmine on recovery from septic shock following intra-abdominal infection - Results from a randomized, double-blind, placebo-controlled, monocentric pilot trial (Anticholium R per Se). | J Crit Care | Pinder 2019 | 20 | No | Therapeutic interventions | Europe | No | No | Yes - clinicaltrials.gov | Yes | Other | No | Industry |
| Effect of Prolonged-Release Pirfenidone on Renal Function in Septic Acute Kidney Injury Patients: A Double-Blind Placebo-Controlled Clinical Trial | International Journal of Nephrology | Chavez-Iniguez 2021 | 88 | Yes | Therapeutic interventions | Latin America | No | No | Yes - clinicaltrials.gov | Yes | Other | No | Non-industry |
| Effect of selenium supplementation on biochemical markers and outcome in critically ill patients | Clin Nutr | Mishra 2007 | 22 | No | Therapeutic interventions | Europe | No | No | No | No | Other | No | Non-industry |
| Effect of Sitting Baduanjin exercise on early rehabilitation of sepsis patients with non-invasive ventilation : a randomized controlled trial. | BMC Complementary Medicine and Therapies | Chen 2024 | 96 | Yes | Other strategies | Asia/Pacific | No | No | Yes - Other | Yes | Other | Yes | Non-industry |
| Effect of small-dose levosimendan on mortality rates and organ functions in Chinese elderly patients with sepsis | Clinical Interventions in Aging | Wang 2017 | 240 | Yes | Therapeutic interventions | Asia/Pacific | No | No | No | No | Mortality | No | Not reported |
| Effect of sodium selenite administration and procalcitonin-guided therapy on mortality in patients with severe sepsis or septic shock: a randomized clinical trial | JAMA | Bloos 2016 | 1180 | Yes | Therapeutic interventions | Europe | No | Yes | Yes - clinicaltrials.gov | Yes | Mortality | No | Non-industry |
| Effect of stress doses of hydrocortisone on S-100B vs. interleukin-8 and polymorphonuclear elastase levels in human septic shock | Clin Chem Lab Med | Mussack 2005 | 12 | No | Therapeutic interventions | Europe | No | No | No | No | Other | Yes | Not reported |
| Effect of tanshinone iia on platelet parameters, coagulation function and blood lactate in patients with sepsis | Acta Medica Mediterranea | Xia 2020 | 42 | No | Therapeutic interventions | Asia/Pacific | No | No | No | No | Other | Yes | Non-industry |
| Effect of Targeted Polymyxin B Hemoperfusion on 28-Day Mortality in Patients With Septic Shock and Elevated Endotoxin Level: The EUPHRATES Randomized Clinical Trial | JAMA | Dellinger 2018 | 226 | No | Therapeutic interventions | North America | Yes | Yes | Yes - clinicaltrials.gov | Yes | Mortality | No | Industry |
| Effect of the antiendotoxic agent, taurolidine, in the treatment of sepsis syndrome: A placebo-controlled, double-blind trial (vol 23, pg 1033, 1995) | Crit Care Med | Willatts 1996 | 100 | No | Therapeutic interventions | Europe | No | Yes | No | No | Mortality | No | Industry |
| Effect of the Shenfu Injection Combined with Early Goal-Directed Therapy on Organ Functions and Outcomes of Septic Shock Patients | Cell Biochem Biophys | Li 2015 | 24 | No | Therapeutic interventions | Asia/Pacific | No | No | No | No | Other | Yes | Non-industry |
| Effect of therapeutic drug monitoring-based dose optimization of piperacillin/tazobactam on sepsis-related organ dysfunction in patients with sepsis: a randomized controlled trial | Intensive Care Med | Hagel 2022 | 249 | No | Other strategies | Europe | No | Yes | Yes - Other | Yes | Other | No | Non-industry |
| Effect of Thiamine on Clinical Outcomes in Septic Shock Patients: a Randomized, Double-Blinded Pilot Study | Am J Respir Crit Care Med | Pereira 2023 | 122 | Yes | Therapeutic interventions | Latin America | No | No | Yes - Other | Yes | Mortality | No | Non-industry |
| Effect of transcutaneous electrical muscle stimulation on muscle volume in patients with septic shock | Crit Care Med | Poulsen 2011 | 80 | No | Other strategies | Europe | No | No | Yes - clinicaltrials.gov | Yes | Other | No | Non-industry |
| Effect of treatment with low doses of hydrocortisone and fludrocortisone on mortality in patients with septic shock | JAMA | Annane 2002 | 300 | Yes | Therapeutic interventions | Europe | No | Yes | No | Yes | Mortality | Yes | Non-industry |
| Effect of two volume responsiveness evaluation methods on fluid resuscitation and prognosis in septic shock patients | Chin Med J | Xu 2014 | 58 | No | Monitoring systems | Asia/Pacific | No | No | No | No | Other | No | Non-industry |
| Effect of Vitamin C Infusion on Organ Failure and Biomarkers of Inflammation and Vascular Injury in Patients With Sepsis and Severe Acute Respiratory Failure: the CITRIS-ALI Randomized Clinical Trial | JAMA | Fowler 2019 | 167 | No | Therapeutic interventions | North America | No | Yes | Yes - clinicaltrials.gov | Yes | Other | No | Non-industry |
| Effect of Vitamin C, Hydrocortisone, and Thiamine vs Hydrocortisone Alone on Time Alive and Free of Vasopressor Support Among Patients With Septic Shock: the VITAMINS Randomized Clinical Trial | JAMA | Fujii 2020 | 211 | Yes | Therapeutic interventions | More than one | Yes | Yes | Yes - clinicaltrials.gov | Yes | Other | No | Non-industry |
| Effect of Vitamin C, Thiamine, and Hydrocortisone on Ventilator- And Vasopressor-Free Days in Patients with Sepsis- And VICTAS Randomized Clinical Trial | JAMA | Sevransky 2021 | 501 | Yes | Therapeutic interventions | North America | No | Yes | Yes - clinicaltrials.gov | Yes | Other | No | Non-industry |
| Effect on extrapulmonary sepsis-induced acute lung injury by hemoperfusion with neutral microporous resin column | Ther Apher Dial | Huang 2013 | 25 | No | Other strategies | Asia/Pacific | No | No | No | No | Other | Yes | Not reported |
| Effectiveness and safety of Shenfu injection in septic patients with hypoperfusion: A multi-center, open-label, randomized, controlled trial. | J Intensive Care Med | Liu 2024 | 192 | Yes | Monitoring systems | Asia/Pacific | No | Yes | Yes - Other | Yes | Mortality | No | Non-industry |
| Effectiveness of enteral ivabradine for heart rate control in septic shock: A randomised controlled trial | Anaesth Intensive Care | Datta 2021 | 60 | Yes | Therapeutic interventions | Asia/Pacific | No | No | Yes - Other | Yes | Other | Yes | Non-industry |
| Effects of blood transfusion on oxygen transport variables in severe sepsis | Crit Care Med | Lorente 1993 | 16 | No | Other strategies | Europe | No | No | No | No | Other | No | Non-industry |
| Effects of capillary refill time-vs. lactate-targeted fluid resuscitation on regional, microcirculatory and hypoxia-related perfusion parameters in septic shock: a randomized controlled trial | Ann Intensive Care | Castro 2020 | 42 | No | Monitoring systems | Latin America | No | Yes | Yes - clinicaltrials.gov | Yes | Other | No | Non-industry |
| Effects of carrimycin on biomarkers of inflammation and immune function in tumor patients with sepsis: a multicenter double-blind randomized controlled trial | Pharmacol Res | Nan 2023 | 120 | No | Therapeutic interventions | Asia/Pacific | No | Yes | Yes - Other | Yes | Other | Yes | Industry |
| Effects of comprehensive nursing on negative emotion and prognosis of patients with sepsis | Am J Transl Re | Shen 2021 | 104 | Yes | Other strategies | Asia/Pacific | No | No | No | No | Other | Yes | Non-industry |
| Effects of continuous haemofiltration <i>vs</i> intermittent haemodialysis on systemic haemodynamics and splanchnic regional perfusion in septic shock patients:: a prospective, randomized clinical trial | Nephrology Dialysis Transplantation | John 2001 | 30 | No | Other strategies | Europe | No | No | Yes - Other | No | Other | No | Not reported |
| Effects of continuous renal replacement therapy on inflammation-related anemia, iron metabolism and prognosis in sepsis patients with acute kidney injury | World J Emerg Med | An 2023 | 99 | Yes | Other strategies | Asia/Pacific | No | No | Yes - Other | Yes | Other | Yes | Non-industry |
| Effects of corticotropin-releasing hormone on proopiomelanocortin derivatives and monocytic HLA-DR expression in patients with septic shock | Peptides | Matejec 2013 | 16 | No | Therapeutic interventions | Europe | No | No | No | No | Other | Yes | Not reported |
| Effects of crude rhubarb on intestinal permeability in septic patients | Am J Chin Med | Fang 2007 | 22 | No | Therapeutic interventions | Asia/Pacific | No | No | No | No | Other | Yes | Non-industry |
| Effects of dexmedetomidine on renal function in patients with septic shock | European Journal of Inflammation | Chen 2018 | 80 | No | Therapeutic interventions | Asia/Pacific | No | No | No | No | Other | Yes | Non-industry |
| Effects of dobutamine on systemic, regional and microcirculatory perfusion parameters in septic shock. A randomized, placebo-controlled, double-blind, crossover study | Intensive Care Med | Hernandez 2013 | 20 | Yes | Therapeutic interventions | Latin America | No | No | Yes - clinicaltrials.gov | Yes | Other | No | Non-industry |
| Effects of dopamine, norepinephrine, and epinephrine on the splanchnic circulation in septic shock: which is best? | Crit Care Med | DeBacker 2003 | 20 | No | Therapeutic interventions | Europe | No | No | No | No | Other | Yes | Non-industry |
| Effects of Early Continuous Venovenous Hemofiltration on E-Selectin, Hemodynamic Stability, and Ventilatory Function in Patients with Septic-Shock-Induced Acute Respiratory Distress Syndrome | Biomed Res Int | Meng 2016 | 51 | No | Other strategies | Asia/Pacific | No | No | No | No | Other | Yes | Non-industry |
| Effects of early enteral nutrition on Th17/Treg cells and IL-23/IL-17 in septic patients | Ann Intensive Care | Sun 2019 | 53 | No | Other strategies | Asia/Pacific | No | No | Yes - clinicaltrials.gov | Yes | Other | Yes | Non-industry |
| Effects of Early Use of Methylene Blue and Vasopressin on Noradrenaline Dose in Septic Shock: A Randomized Controlled Trial | Indian J Crit Care Med | Kuri 2025 | 74 | Yes | Therapeutic interventions | Asia/Pacific | No | No | Yes - Other | Yes | Other | Yes | Non-industry |
| Effects of endotoxin adsorber hemoperfusion on sublingual microcirculation in patients with septic shock: a randomized controlled trial | Ann Intensive Care | Chen 2020 | 28 | No | Other strategies | Asia/Pacific | No | No | Yes - clinicaltrials.gov | Yes | Other | Yes | Non-industry |
| Effects of enteral feeding with eicosapentaenoic acid, gamma-linolenic acid, and antioxidants in mechanically ventilated patients with severe sepsis and septic shock | Crit Care Med | Pontes-Arruda 2006 | 165 | No | Other strategies | Latin America | No | No | No | No | Mortality | Yes | Industry |
| Effects of epinephrine, norepinephrine, or the combination of norepinephrine and dobutamine on gastric mucosa in septic shock | Crit Care Med | Duranteau 1999 | 12 | No | Therapeutic interventions | Europe | No | No | No | No | Other | Yes | Non-industry |
| Effects of Esketamine Versus Remifentanil on Hemodynamics and Prognosis in Patients with Septic Shock Receiving Invasive Mechanical Ventilation: A Randomized Controlled Trial. | Drug Design, Development and Therapy | Li 2025 | 120 | Yes | Therapeutic interventions | Asia/Pacific | No | No | Yes - clinicaltrials.gov | Yes | Other | Yes | Non-industry |
| Effects of fluid resuscitation under the guidance of PICCO on the immune function and inflammatory mediator in patients with septic shock | Biomed Res Int | Yao 2017 | 180 | No | Other strategies | Asia/Pacific | No | No | No | No | Other | Yes | Not reported |
| Effects of high doses of selenium, as sodium selenite, in septic shock: a placebo-controlled, randomized, double-blind, phase II study | Crit Care | Forceville 2007 | 60 | Yes | Therapeutic interventions | Europe | No | Yes | Yes - clinicaltrials.gov | Yes | Other | No | Non-industry |
| Effects of high-dose of intravenous immunoglobulin and antibiotics on survival for severe sepsis undergoing surgery | Shock | Rodriguez 2005 | 56 | No | Therapeutic interventions | More than one | Yes | Yes | No | No | Mortality | No | Industry |
| Effects of hydrocortisone combined with vitamin C and vitamin B1 versus hydrocortisone alone on microcirculation in septic shock patients: a pilot study | Clinical Hemorheology and Microcirculation | Wang 2023 | 27 | Yes | Therapeutic interventions | Asia/Pacific | No | No | Yes - clinicaltrials.gov | Yes | Other | Yes | Not reported |
| Effects of hydroxyethylstarch and gelatin on renal function in severe sepsis: a multicentre randomised study | Lancet | Schortgen 2001 | 129 | No | Other strategies | Europe | No | Yes | No | No | Other | Yes | Non-industry |
| Effects of ibuprofen on the physiology and survival of hypothermic sepsis | Crit Care Med | Arons 1999 | 455 | No | Therapeutic interventions | North America | No | Yes | No | Yes | Other | Yes | Non-industry |
| Effects of IgM-enriched immunoglobulin therapy in septic-shock-induced multiple organ failure: pilot study | J Anesth | Toth 2013 | 33 | No | Therapeutic interventions | Europe | No | No | No | No | Other | No | Not reported |
| Effects of Increasing Hydrocortisone to 300 mg Per Day in the Treatment of Septic Shock: a Pilot Study | Shock | Hyvernat 2016 | 122 | Yes | Therapeutic interventions | Europe | No | No | Yes - clinicaltrials.gov | Yes | Mortality | No | Non-industry |
| Effects of Levosimendan on Cellular Metabolic Alterations in Patients With Septic Shock: a Randomized Controlled Pilot Study | Shock | Hajjej 2017 | 20 | Yes | Therapeutic interventions | Asia/Pacific | No | No | Yes - clinicaltrials.gov | Yes | Other | Yes | Not reported |
| Effects of levosimendan on mitochondrial function in patients with septic shock: A randomized trial | BIOCHIMIE | Torraco 2014 | 26 | No | Therapeutic interventions | Europe | No | No | No | No | Other | Yes | Non-industry |
| Effects of levosimendan on right ventricular afterload in patients with acute respiratory distress syndrome: a pilot study | Crit Care Med | Morelli 2006 | 18 | No | Therapeutic interventions | Europe | Yes | Yes | No | No | Other | Yes | Non-industry |
| Effects of levosimendan on systemic and regional hemodynamics in septic myocardial depression | Intensive Care Med | Morelli 2005 | 15 | No | Therapeutic interventions | Europe | No | Yes | No | No | Other | Yes | Non-industry |
| Effects of lornoxicam on the physiology of severe sepsis | Crit Care | memis 2004 | 40 | No | Therapeutic interventions | Europe | No | No | No | No | Other | No | Not reported |
| Effects of low-dose furosemide combined with aminophylline on the renal function in septic shock patients | Renal failure | Mai 2023 | 109 | Yes | Therapeutic interventions | Asia/Pacific | No | No | Yes - Other | Yes | Other | Yes | Non-industry |
| Effects of Melatonin and Propolis Supplementation on Inflammation, Oxidative Stress, and Clinical Outcomes in Patients with Primary Pneumosepsis: A Randomized Controlled Clinical Trial | Complementary Medicine Research | Pahlavani 2022 | 55 | No | Therapeutic interventions | Asia/Pacific | No | No | Yes - Other | Yes | Other | Yes | Non-industry |
| Effects of N-acetylcysteine on microalbuminuria and organ failure in acute severe sepsis: results of a pilot study | Chest | Spapen 2005 | 18 | No | Therapeutic interventions | Europe | No | No | No | No | Other | No | Non-industry |
| Effects of nanocurcumin on inflammatory factors and clinical outcomes in critically ill patients with sepsis: a pilot randomized clinical trial | European Journal of Integrative Medicine | Karimi 2020 | 40 | No | Therapeutic interventions | Asia/Pacific | No | Yes | Yes - Other | Yes | Other | Yes | Non-industry |
| Effects of nitroglycerin on sublingual microcirculatory blood flow in patients with severe sepsis/septic shock after a strict resuscitation protocol: a double-blind randomized placebo controlled trial | Crit Care Med | Boerma 2010 | 35 | No | Therapeutic interventions | Europe | No | No | Yes - clinicaltrials.gov | No | Other | No | Non-industry |
| Effects of norepinephrine, epinephrine, and norepinephrine-dobutamine on systemic and gastric mucosal oxygenation in septic shock | Acta Pharmacol Sin | Zhou 2002 | 16 | No | Therapeutic interventions | Asia/Pacific | No | No | No | No | Other | Yes | Not reported |
| Effects of parenteral glutamine supplementation on endocan levels in septic patients | East J Med | Kilic 2021 | 60 | No | Other strategies | Europe | No | No | No | No | Other | No | Not reported |
| Effects of pentoxifylline on circulating cytokine concentrations and hemodynamics in patients with septic shock: results from a double-blind, randomized, placebo-controlled study | Crit Care Med | Zeni 1996 | 16 | No | Therapeutic interventions | Africa | No | No | No | No | Other | Yes | Not reported |
| Effects of propofol and dexmedetomidine on indocyanine green elimination assessed with L√Ñ¬∞MON to patients with early septic shock: a pilot study | J Crit Care | Memis 2009 | 20 | No | Therapeutic interventions | Europe | No | No | No | No | Other | No | Non-industry |
| Effects of recombinant human growth hormone in patients with severe sepsis | Ann Surg | Voerman 1992 | 20 | No | Therapeutic interventions | Europe | No | No | No | No | Other | Yes | Industry |
| Effects of Shenfu Injection in the Treatment of Septic Shock Patients: a Multicenter, Controlled, Randomized, Open-Label Trial | Evid Based Complement Alternat Med | Li 2016 | 210 | No | Therapeutic interventions | Asia/Pacific | No | Yes | Yes - Other | Yes | Other | No | Industry |
| Effects of Shenfu injection on sublingual microcirculation in septic shock patients: a randomized controlled trial | Shock | Wang 2022 | 40 | Yes | Therapeutic interventions | Asia/Pacific | No | No | No | No | Other | Yes | Non-industry |
| Effects of short-term fenoldopam infusion on gastric mucosal blood flow in septic shock | Anesthesiology | Morelli 2004 | 20 | No | Therapeutic interventions | Europe | No | Yes | No | No | Other | Yes | Non-industry |
| Effects of short-term simultaneous infusion of dobutamine and terlipressin in patients with septic shock: the DOBUPRESS study | Br J Anaesth | Morelli 2008 | 20 | No | Therapeutic interventions | Europe | Yes | Yes | No | No | Other | Yes | Non-industry |
| Effects of target temperature management on the outcome of septic patients with fever | BioMed Research International | Gao 2017 | 63 | Yes | Other strategies | Asia/Pacific | No | Yes | No | No | Other | Yes | Non-industry |
| Effects of thiamine on balance between matrix metalloproteinases-9 (Mmp9) and tissue inhibitors of metalloproteinases-1 (timp-1) | Journal of Drug and Alcohol Research | Lubis 2021 | 72 | No | Therapeutic interventions | Asia/Pacific | No | Yes | No | No | Other | Yes | Not reported |
| Effects of thiamine on vasopressor requirements in patients with septic shock: a prospective randomized controlled trial | BMC Anesthesiol | Petsakul 2020 | 50 | Yes | Therapeutic interventions | Asia/Pacific | No | No | Yes - Other | Yes | Other | No | Non-industry |
| Effects of triple combination of hydrocortisone, thiamine, and Vitamin C on clinical outcome in patients with septic shock: a single-center randomized controlled trial | Journal of Research in Medical Sciences | Jamshidi 2021 | 58 | Yes | Therapeutic interventions | Asia/Pacific | No | No | Yes - Other | Yes | Other | Yes | Non-industry |
| Effects of vasopressinergic receptor agonists on sublingual microcirculation in norepinephrine-dependent septic shock | Crit Care | Morelli 2011 | 20 | No | Therapeutic interventions | Europe | Yes | Yes | Yes - clinicaltrials.gov | Yes | Other | No | Not reported |
| Efficacy and Prognosis of HA380 Perfusion Combined with Continuous Venovenous Hemofiltration in the Treatment of Sepsis | J Biol Regul Homeost Agents | Wang 2024 | 60 | No | Therapeutic interventions | Asia/Pacific | No | No | Yes - Other | Yes | Other | Yes | Non-industry |
| Efficacy and Safety of a Balanced Gelatine Solution for Fluid Resuscitation in Sepsis: A Prospective, Randomised, Controlled, Double-Blind Trial-GENIUS Trial. | Journal of clinical medicine | Marx 2025 | 167 | No | Other strategies | More than one | Yes | Yes | Yes - clinicaltrials.gov | Yes | Other | No | Industry |
| Efficacy and safety of a phospholipid emulsion (GR270773) in Gram-negative severe sepsis: results of a phase II multicenter, randomized, placebo-controlled, dose-finding clinical trial | Crit Care Med | Dellinger 2009 | 1379 | No | Therapeutic interventions | More than one | Yes | Yes | No | Yes | Mortality | No | Industry |
| Efficacy and Safety of Continuous vs Intermittent Linezolid Infusion in Critically Ill Patients with Septic Shock | Indian J Crit Care Med | Albadry 2024 | 140 | No | Therapeutic interventions | Africa | No | Yes | Yes - clinicaltrials.gov | No | Other | Yes | Not reported |
| Efficacy and safety of dopamine versus norepinephrine in the management of septic shock | Shock | Patel 2010 | 252 | No | Therapeutic interventions | North America | No | No | Yes - clinicaltrials.gov | Yes | Mortality | No | Non-industry |
| Efficacy and safety of landiolol, an ultra-short-acting beta1-selective antagonist, for treatment of sepsis-related tachyarrhythmia (J-Land 3S): a multicentre, open-label, randomised controlled trial. | Lancet | Kakihana 2020 | 151 | Yes | Therapeutic interventions | Asia/Pacific | No | Yes | Yes - Other | Yes | Other | Yes | Industry |
| Efficacy and safety of LY315920Na/S-5920, a selective inhibitor of 14-kDa group IIA secretory phospholipase A2, in patients with suspected sepsis and organ failure | Crit Care Med | Abraham 2003 | 586 | Yes | Therapeutic interventions | North America | No | Yes | No | No | Mortality | No | Industry |
| Efficacy and safety of procalcitonin guidance in reducing the duration of antibiotic treatment in critically ill patients: a randomised, controlled, open-label trial | Lancet Infect Dis | deJong 2016 | 4507 | No | Therapeutic interventions | Europe | No | Yes | Yes - clinicaltrials.gov | Yes | Other | Yes | Industry |
| Efficacy and safety of recombinant human activated protein C for severe sepsis | N Engl J Med | Bernard 2001 | 1690 | Yes | Therapeutic interventions | More than one | Yes | Yes | No | Yes | Mortality | Yes | Industry |
| Efficacy and Safety of Recombinant Human Thrombopoietin (rhTPO) on Coagulation Function and Inflammatory Factors in the Treatment of Patients with Sepsis-Related Thrombocytopenia. | Clinical and Applied Thrombosis/Hemostasis | Wang 2025 | 144 | Yes | Therapeutic interventions | Asia/Pacific | No | No | No | No | Other | No | Non-industry |
| Efficacy and safety of the monoclonal anti-tumor necrosis factor antibody F(ab')2 fragment afelimomab in patients with severe sepsis and elevated interleukin-6 levels | Crit Care Med | Panacek 2004 | 2634 | Yes | Therapeutic interventions | North America | Yes | Yes | No | No | Mortality | Yes | Industry |
| Efficacy and safety of the platelet-activating factor receptor antagonist BN 52021 (Ginkgolide B) in patients with severe sepsis: a randomised, double-blind, placebo-controlled, multicentre trial | Clin Drug Investig | Albrecht 2004 | 88 | No | Therapeutic interventions | Europe | No | Yes | No | No | Mortality | No | Industry |
| Efficacy and safety of tifacogin (recombinant tissue factor pathway inhibitor) in severe sepsis: a randomized controlled trial | JAMA | Abraham 2003 | 1754 | No | Therapeutic interventions | More than one | Yes | Yes | No | No | Mortality | No | Industry |
| Efficacy and Safety of Vilobelimab (IFX-1), a Novel Monoclonal Anti-C5a Antibody, in Patients With Early Severe Sepsis or Septic Shock-A Randomized, Placebo-Controlled, Double-Blind, Multicenter, Phase IIa Trial (SCIENS Study) | Crit Care Explor | Bauer 2021 | 72 | Yes | Therapeutic interventions | Europe | No | Yes | Yes - clinicaltrials.gov | Yes | Other | Yes | Industry |
| Efficacy of adjuvant use of midodrine in patients with septic shock: An open label randomized controlled trial. | Pharmacotherapy | El-Nagdy 2025 | 100 | Yes | Therapeutic interventions | Africa | No | No | Yes - clinicaltrials.gov | Yes | Mortality | No | Not reported |
| Efficacy of Continuous vs. Intermittent Administration of Cefepime in Adult ICU Patients with Gram-Negative Bacilli Bacteremia: A Randomized Double-Blind Clinical Study. | Antibiotics | Alvarez-Moreno 2024 | 32 | No | Therapeutic interventions | Latin America | No | Yes | Yes - clinicaltrials.gov | Yes | Other | No | Non-industry |
| Efficacy of coupled plasma filtration adsorption (CPFA) in patients with septic shock: a multicenter randomised controlled clinical trial | BMJ Open | Livigni 2014 | 192 | No | Other strategies | Europe | No | Yes | Yes - clinicaltrials.gov | Yes | Mortality | No | Industry |
| Efficacy of Levosimendan in the Treatment of Patients With Severe Septic Cardiomyopathy | J Cardiothorac Vasc Anesth | Sun 2023 | 30 | No | Therapeutic interventions | Asia/Pacific | No | No | No | No | Mortality | No | Non-industry |
| Efficacy of phenylephrine versus noradrenaline in management of patients presenting with septic shock in the intensive care unit | Rawal Medical Journal | Hussain 2014 | 42 | No | Therapeutic interventions | Asia/Pacific | No | No | No | No | Other | Yes | Not reported |
| Efficacy of single-dose intravenous immunoglobulin administration for severe sepsis and septic shock | J Intensive Care | Hamano 2013 | 79 | No | Therapeutic interventions | Asia/Pacific | No | No | No | No | Other | Yes | Not reported |
| Efficacy of targeting high mean arterial pressure for older patients with septic shock (OPTPRESS): a multicentre, pragmatic, open-label, randomised controlled trial. | Intensive Care Med | Endo 2025 | 518 | Yes | Monitoring systems | Asia/Pacific | No | Yes | Yes - Other | Yes | Mortality | Yes | Non-industry |
| Efficacy of ulinastatin combined with alanyglutamine for patients with sepsis | Int J Clin Exp Med | Yuan 2020 | 84 | No | Therapeutic interventions | Asia/Pacific | No | No | No | No | Other | Yes | Not reported |
| Efficacy of xuebijing injection in the adjunctive therapy of acute respiratory distress syndrome caused by sepsis | Int J Clin Exp Med | Zhen 2019 | 32 | No | Therapeutic interventions | Asia/Pacific | No | Yes | No | No | Other | Yes | Not reported |
| Electric Muscle Stimulation for Weaning from Mechanical Ventilation in Elder Patients with Severe Sepsis and Acute Respiratory Failure √¢¬Ä¬ì A Pilot Study | International Journal of Gerontology | Shen 2017 | 25 | Yes | Other strategies | Asia/Pacific | No | No | No | No | Other | Yes | Non-industry |
| Electro-acupuncture attenuates inflammatory responses and intraabdominal pressure in septic patients: a randomized controlled trial | Medicine | Meng 2018 | 82 | Yes | Other strategies | Asia/Pacific | No | No | Yes - Other | Yes | Other | Yes | Non-industry |
| Electroacupuncture Improves Intestinal Dysfunction in Septic Patients: a Randomised Controlled Trial | Biomed Res Int | Meng 2018 | 71 | No | Other strategies | Asia/Pacific | No | Yes | Yes - Other | Yes | Other | Yes | Non-industry |
| Elevation of cardiac output and oxygen delivery improves outcome in septic shock | Chest | Tuchschmidt 1992 | 26 | No | Care bundles | North America | No | No | No | No | Mortality | No | Not reported |
| Empirical Micafungin Treatment and Survival Without Invasive Fungal Infection in Adults With ICU-Acquired Sepsis, <i>Candida</i> Colonization, and Multiple Organ Failure The EMPIRICUS Randomized Clinical Trial | JAMA | EMPIRICUSTrialGrp 2016 | 260 | Yes | Therapeutic interventions | Europe | No | Yes | Yes - clinicaltrials.gov | Yes | Other | No | Non-industry |
| Endotoxin and cytokin reduction function of the oXiris filter in a prospective double-blinded cross-over setting in patients with critical Gram-septic shock and continuous renal replacement therapy requiring acute kidney injury | PLoS One | Broman 2018 | 16 | No | Therapeutic interventions | Europe | No | Yes | Yes - clinicaltrials.gov | Yes | Other | Yes | Industry |
| Endotoxin Removal in Septic Shock with the Alteco LPS Adsorber Was Safe But Showed no Benefit Compared to Placebo in the Double-Blind Randomized Controlled Trial-the Asset Study | Shock | Lipcsey 2020 | 15 | Yes | Other strategies | Europe | Yes | Yes | Yes - clinicaltrials.gov | Yes | Other | No | Industry |
| Enoximone in contrast to dobutamine improves hepatosplanchnic function in fluid-optimized septic shock patients | Crit Care Med | Kern 2001 | 48 | No | Therapeutic interventions | Europe | No | No | No | No | Other | Yes | Not reported |
| Enteral nutrition with eicosapentaenoic acid, gamma-linolenic acid and antioxidants in the early treatment of sepsis: results from a multicenter, prospective, randomized, double-blinded, controlled study: the INTERSEPT study. | Crit Care | Pontes-Arruda 2011 | 106 | Yes | Other strategies | Latin America | No | Yes | Yes - clinicaltrials.gov | Yes | Other | Yes | Industry |
| Enteral nutrition with omega-3 fatty acids in critically ill septic patients: a randomized double-blinded study | Saudi J Anaesth | Ibrahim 2018 | 55 | No | Other strategies | Africa | No | No | No | No | Other | Yes | Not reported |
| Evaluating the effect of dexmedetomidine on hemodynamic status of patients with septic shock admitted to intensive care unit: a single-blind randomized controlled trial | Iran J Pharm Res | Gheibi 2020 | 66 | No | Therapeutic interventions | Asia/Pacific | No | No | Yes - Other | Yes | Other | Yes | Non-industry |
| Evaluating the effects of curcumin nanomicelles on clinical outcome and cellular immune responses in critically ill sepsis patients: A randomized, double-blind, and placebo-controlled trial | Front Nutr | Karimi 2022 | 40 | No | Therapeutic interventions | Asia/Pacific | No | No | Yes - Other | Yes | Other | Yes | Non-industry |
| Evaluating the effects of Esmolol on cardiac function in patients with Septic cardiomyopathy by Speck-tracking echocardiography-a randomized controlled trial | BMC Anesthesiol | Wang 2023 | 100 | Yes | Therapeutic interventions | Asia/Pacific | No | Yes | Yes - Other | Yes | Other | Yes | Non-industry |
| Evaluating the efficacy and safety of two doses of the polyclonal anti-tumor necrosis factor-√é¬± fragment antibody AZD9773 in adult patients with severe sepsis and/or septic shock: randomized, double-blind, placebo-controlled phase IIb study* | Crit Care Med | Bernard 2014 | 100 | No | Therapeutic interventions | Europe | Yes | Yes | Yes - clinicaltrials.gov | Yes | Other | No | Industry |
| Evaluating Vitamin C in Septic Shock: a Randomized Controlled Trial of Vitamin C Monotherapy | Crit Care Med | Wacker 2022 | 124 | Yes | Therapeutic interventions | North America | No | Yes | Yes - Other | Yes | Mortality | No | Non-industry |
| Evaluation of endotoxin release and cytokine production induced by antibiotics in patients with Gram-negative nosocomial pneumonia | Crit Care Med | Maskin 2002 | 20 | No | Therapeutic interventions | Latin America | No | No | No | No | Other | No | Industry |
| Evaluation of Hydrocortisone, Vitamin C, and Thiamine for the Treatment of Septic Shock: a Randomized Controlled Trial (The HYVITS Trial) | Shock | Mohamed 2023 | 106 | Yes | Therapeutic interventions | Asia/Pacific | No | Yes | Yes - clinicaltrials.gov | Yes | Mortality | No | Non-industry |
| Evaluation of the Effects of a Combination of Vitamin C, Thiamine and Hydrocortisone vs Hydrocortisone Alone on ICU Outcome in Patients with Septic Shock: A Randomized Controlled Trial. | Indian J Crit Care Med | Sharma 2024 | 91 | Yes | Care bundles | Asia/Pacific | No | No | No | No | Mortality | No | Not reported |
| Evaluation of the safety and efficacy of beta blockers in septic patients: a randomized control trial | AIN SHAMS JOURNAL OF ANESTHESIOLOGY | Gadallah 2020 | 60 | No | Therapeutic interventions | Africa | No | No | No | No | Other | Yes | Not reported |
| Examination of setarud (IMOD√¢¬Ñ¬¢) in the management of patients with severe sepsis | Daru | Mahmoodpoor 2010 | 20 | No | Therapeutic interventions | Asia/Pacific | No | No | Yes - Other | Yes | Other | Yes | Industry |
| Exploring choices of early nutritional support for patients with sepsis based on changes in intestinal microecology | Crit Care | Yang 2023 | 197 | Yes | Monitoring systems | Asia/Pacific | No | No | No | No | Mortality | Yes | Not reported |
| Extended drotrecogin alfa (activated) treatment in patients with prolonged septic shock | Intensive Care Med | Dhainaut 2009 | 193 | No | Therapeutic interventions | More than one | Yes | Yes | No | No | Other | No | Industry |
| Extracorporeal cytokine adsorption in septic shock: a proof of concept randomized, controlled pilot study | J Crit Care | Hawchar 2019 | 20 | No | Other strategies | Europe | No | No | Yes - clinicaltrials.gov | Yes | Other | Yes | Non-industry |
| Extravascular lung water monitoring of renal replacement therapy in lung water scavenging for septic acute kidney injury | Int J Clin Exp Med | Liu 2015 | 57 | Yes | Monitoring systems | Asia/Pacific | No | No | No | No | Other | Yes | Not reported |
| Feasibility Assessment of a Biomarker-Guided Kidney-Sparing Sepsis Bundle: The Limiting Acute Kidney Injury Progression In Sepsis Trial. | Crit Care Explor | Gomez 2023 | 19 | Yes | Care bundles | North America | No | Yes | Yes - clinicaltrials.gov | Yes | Other | Yes | Non-industry |
| Fever control using external cooling in septic shock: a randomized controlled trial | Am J Respir Crit Care Med | Schortgen 2012 | 101 | No | Other strategies | Europe | No | Yes | No | No | Other | No | Non-industry |
| Filgrastim in patients with pneumonia and severe sepsis or septic shock | Chest | Wunderink 2001 | 12 | No | Therapeutic interventions | North America | No | Yes | No | No | Other | No | Industry |
| Fluconazole improves survival in septic shock: a randomized double-blind prospective study | Crit Care Med | Jacobs 2003 | 71 | No | Therapeutic interventions | Asia/Pacific | No | No | No | No | Mortality | Yes | Not reported |
| Fludrocortisone dose-response relationship in septic shock: a randomised phase II trial. | Intensive Care Med | Walsham 2024 | 153 | No | Therapeutic interventions | Asia/Pacific | No | Yes | Yes - clinicaltrials.gov | Yes | Other | No | Non-industry |
| Fluid Response Evaluation in Sepsis Hypotension and Shock: a Randomized Clinical Trial | Chest | Douglas 2020 | 150 | Yes | Monitoring systems | More than one | Yes | Yes | Yes - clinicaltrials.gov | Yes | Other | Yes | Industry |
| Fluid resuscitation with colloids of different molecular weight in septic shock | Intensive Care Med | Molnar 2004 | 15 | No | Other strategies | Europe | No | No | No | No | Other | No | Non-industry |
| Gastric tonometry versus cardiac index as resuscitation goals in septic shock: a multicenter, randomized, controlled trial | Crit Care | Palizas 2009 | 66 | No | Monitoring systems | More than one | Yes | Yes | No | No | Mortality | No | Non-industry |
| Glibenclamide dose response in patients with septic shock | Crit Care | Morelli 2007 | 30 | No | Therapeutic interventions | Europe | Yes | Yes | No | No | Other | No | Non-industry |
| Global end-diastolic volume index vs CVP goal-directed fluid resuscitation for COPD patients with septic shock: a randomized controlled trial | Am J Emerg Med | Yu 2017 | 71 | Yes | Care bundles | Asia/Pacific | No | No | Yes - Other | Yes | Other | No | Not reported |
| Granulocyte-macrophage colony-stimulating factor (GM-CSF) in patients presenting sepsis-induced immunosuppression: the GRID randomized controlled trial | J Crit Care | Vacheron 2023 | 98 | No | Therapeutic interventions | Europe | No | Yes | Yes - clinicaltrials.gov | Yes | Other | No | Non-industry |
| Granulocyte-macrophage colony-stimulating factor to reverse sepsis-associated immunosuppression: a double-blind, randomized, placebo-controlled multicenter trial | Am J Respir Crit Care Med | Meisel 2009 | 38 | No | Therapeutic interventions | Europe | No | Yes | Yes - clinicaltrials.gov | Yes | Other | Yes | Non-industry |
| Hemodialysis membrane with a high-molecular-weight cutoff and cytokine levels in sepsis complicated by acute renal failure: a phase 1 randomized trial | Am J Kidney Dis | Haase 2007 | 10 | No | Other strategies | Asia/Pacific | No | No | Yes - clinicaltrials.gov | Yes | Other | Yes | Industry |
| Hemodynamic effects of 6% and 10% hydroxyethyl starch solutions versus 4% albumin solution in septic patients | Journal of Clinical Anesthesia | Friedman 2008 | 13 | No | Other strategies | Europe | No | No | No | No | Other | Yes | Industry |
| Hemoperfusion using the LPS-selective mesoporous polymeric adsorbent in septic shock: a multicenter randomized clinical trial | Shock | Rey 2023 | 38 | No | Therapeutic interventions | Europe | No | No | Yes - clinicaltrials.gov | Yes | Other | Yes | Not reported |
| Heparanase inhibition leads to improvement in patients with acute gastrointestinal injuries induced by sepsis | World J Gastroenterol | Chen 2023 | 48 | No | Therapeutic interventions | Asia/Pacific | No | No | Yes - Other | Yes | Other | Yes | Non-industry |
| Heparanase inhibitor improves clinical study in patients with septic cardiomyopathy. | Frontiers in medicine | Chen 2024 | 105 | No | Therapeutic interventions | Asia/Pacific | No | No | Yes - Other | Yes | Other | Yes | Non-industry |
| High versus Low Blood-Pressure Target in Patients with Septic Shock | N Engl J Med | Asfar 2014 | 798 | Yes | Other strategies | Europe | No | Yes | Yes - clinicaltrials.gov | Yes | Mortality | No | Non-industry |
| High-dose antithrombin III in severe sepsis: A randomized controlled trial | JAMA | Warren 2001 | 698 | No | Therapeutic interventions | More than one | Yes | Yes | No | No | Mortality | No | Industry |
| High-Dose IV Hydroxocobalamin (Vitamin B12) in Septic Shock: a Double-Blind, Allocation-Concealed, Placebo-Controlled Single-Center Pilot Randomized Controlled Trial (The Intravenous Hydroxocobalamin in Septic Shock Trial) | Chest | Patel 2023 | 20 | Yes | Therapeutic interventions | North America | No | No | Yes - clinicaltrials.gov | Yes | Other | Yes | Non-industry |
| High-Dose Versus Conventional-Dose Continuous Venovenous Hemodiafiltration and Patient and Kidney Survival and Cytokine Removal in Sepsis-Associated Acute Kidney Injury: A Randomized Controlled Trial | Am J Kidney Dis | HICORESInvestigators 2016 | 212 | Yes | Other strategies | Asia/Pacific | No | Yes | Yes - clinicaltrials.gov | Yes | Mortality | No | Industry |
| High-dose vitamin C improves norepinephrine level in patients with septic shock: A single-center, prospective, randomized controlled trial. | Medicine | Li 2024 | 71 | Yes | Therapeutic interventions | Asia/Pacific | No | No | No | No | Mortality | No | Non-industry |
| High-flow nasal oxygen cannula vs. noninvasive mechanical ventilation to prevent reintubation in sepsis: a randomized controlled trial | Ann Intensive Care | Tongyoo 2021 | 222 | Yes | Other strategies | Asia/Pacific | No | No | Yes - clinicaltrials.gov | Yes | Other | No | Non-industry |
| High-volume haemofiltration in human septic shock | Intensive Care Med | Cole 2001 | 11 | No | Other strategies | Asia/Pacific | No | No | No | No | Other | No | Industry |
| High-volume hemofiltration in adult burn patients with septic shock and acute kidney injury: a multicenter randomized controlled trial | Crit Care | Chung 2017 | 28 | No | Other strategies | North America | No | Yes | Yes - clinicaltrials.gov | Yes | Other | Yes | Non-industry |
| High-volume versus standard-volume haemofiltration for septic shock patients with acute kidney injury (IVOIRE study): a multicentre randomized controlled trial | Intensive Care Med | Joannes-Boyau 2013 | 140 | No | Therapeutic interventions | Europe | Yes | Yes | Yes - clinicaltrials.gov | Yes | Mortality | No | Non-industry |
| Hydrocortisone plus Fludrocortisone for Adults with Septic Shock | N Engl J Med | Annane 2018 | 1241 | No | Therapeutic interventions | Europe | No | Yes | Yes - clinicaltrials.gov | Yes | Mortality | Yes | Non-industry |
| Hydrocortisone Therapy for Patients with Septic Shock | N Engl J Med | Sprung 2008 | 499 | Yes | Therapeutic interventions | More than one | Yes | Yes | Yes - Other | Yes | Mortality | No | Non-industry |
| Hydrocortisone treatment in early sepsis-associated acute respiratory distress syndrome: results of a randomized controlled trial | Crit Care | Tongyoo 2016 | 206 | Yes | Therapeutic interventions | Asia/Pacific | No | No | Yes - clinicaltrials.gov | Yes | Mortality | No | Non-industry |
| Hydrocortisone, vitamin C and thiamine for the treatment of sepsis and septic shock following cardiac surgery | Indian J Anaesth | Balakrishnan 2018 | 24 | No | Therapeutic interventions | Asia/Pacific | No | No | No | Yes | Other | Yes | Not reported |
| Hydroxyethyl starch 130/0.42 increased death at 90 days compared with Ringer's acetate in severe sepsis | N Engl J Med | Perner 2012 | 798 | Yes | Other strategies | Europe | Yes | Yes | Yes - clinicaltrials.gov | Yes | Mortality | No | Non-industry |
| Hyperglycemia Risk Evaluation of Hydrocortisone Intermittent Boluses versus Continuous Infusion in Septic Shock: A Prospective Randomized Trial | Anesthesia: Essays and Research | Ram 2022 | 112 | Yes | Therapeutic interventions | Asia/Pacific | No | No | No | No | Other | No | Non-industry |
| Hyperoxia and hypertonic saline in patients with septic shock (HYPERS2S): a two-by-two factorial, multicentre, randomised, clinical trial | Lancet | Asfar 2017 | 868 | Yes | Therapeutic interventions | Europe | No | Yes | Yes - clinicaltrials.gov | Yes | Mortality | No | Non-industry |
| Hypertonic fluid administration in patients with septic shock: a prospective randomized controlled pilot study | Shock | vanHaren 2012 | 24 | No | Other strategies | Asia/Pacific | No | No | Yes - Other | Yes | Other | No | Non-industry |
| IgM-enriched immunoglobulins (Pentaglobin) may improve the microcirculation in sepsis: a pilot randomized trial | Ann Intensive Care | Domizi 2019 | 20 | Yes | Therapeutic interventions | Europe | No | No | Yes - clinicaltrials.gov | Yes | Other | Yes | Non-industry |
| IgMA-enriched immunoglobulin in neutropenic patients with sepsis syndrome and septic shock: a randomized, controlled, multiple-center trial | Crit Care Med | Hentrich 2006 | 211 | No | Therapeutic interventions | Europe | No | Yes | No | No | Mortality | No | Industry |
| Iloprost and Organ Dysfunction in Adults With Septic Shock and Endotheliopathy: A Randomized Clinical Trial. | JAMA | Bestle 2024 | 279 | Yes | Therapeutic interventions | Europe | No | Yes | Yes - clinicaltrials.gov | Yes | Other | No | Non-industry |
| Immune Checkpoint Inhibition in Sepsis: a Phase 1b Randomized, Placebo-Controlled, Single Ascending Dose Study of Antiprogrammed Cell Death-Ligand 1 Antibody (BMS-936559) | Crit Care Med | Hotchkiss 2019 | 20 | No | Therapeutic interventions | North America | No | Yes | Yes - clinicaltrials.gov | Yes | Other | Yes | Industry |
| Immunologic and hemodynamic effects of "low-dose" hydrocortisone in septic shock: a double-blind, randomized, placebo-controlled, crossover study | Am J Respir Crit Care Med | Keh 2003 | 40 | No | Therapeutic interventions | Europe | No | No | No | No | Other | Yes | Non-industry |
| Immunomodulatory effects of glutamine-enriched nutritional support in elderly patients with severe sepsis: a prospective, randomized, controlled study | Journal of Organ Dysfunction | Cai 2008 | 55 | No | Therapeutic interventions | Asia/Pacific | No | No | No | No | Other | Yes | Non-industry |
| Immunotherapy improves immune homeostasis and increases survival rate of septic patients | Chin J Traumatol | Huang 2009 | 34 | No | Therapeutic interventions | Asia/Pacific | No | No | No | No | Mortality | Yes | Not reported |
| Impact of continuous venovenous hemofiltration on organ failure during the early phase of severe sepsis: a randomized controlled trial | Crit Care Med | Payen 2009 | 80 | No | Other strategies | Europe | No | Yes | No | No | Other | No | Industry |
| Impact of early versus late administration of norepinephrine on the hemodynamic outcome and mortality in septic shock | Anaesth pain intensive care | Elghareeb 2025 | 200 | No | Other strategies | Africa | No | No | No | No | Mortality | Yes | Non-industry |
| Impact of oral omega-3 fatty acids supplementation in early sepsis on clinical outcome and immunomodulation | The Egyptian Journal of Critical Care Medicine | Hosny 2013 | 50 | Yes | Other strategies | Africa | No | No | No | No | Other | Yes | Not reported |
| Impact of using midodrine for vasopressor weaning in septic shock patients: a randomized controlled trial | Anaesth pain intensive care | Mahmoud 2025 | 150 | No | Therapeutic interventions | Africa | No | No | No | No | Other | Yes | Non-industry |
| Impact of Very Early Physical Therapy During Septic Shock on Skeletal Muscle: a Randomized Controlled Trial | Crit Care Med | Hickmann 2018 | 21 | No | Other strategies | Europe | No | No | Yes - clinicaltrials.gov | Yes | Other | No | Non-industry |
| Impact on fluid balance of an optimized restrictive strategy targeting non-resuscitative fluids in intensive care patients with septic shock: a single-blind, multicenter, randomized, controlled, pilot study. | Crit Care | Boulet 2024 | 50 | Yes | Monitoring systems | Europe | No | Yes | Yes - clinicaltrials.gov | Yes | Other | No | Non-industry |
| Impacts of different hemofiltration methods on the prognosis of patients with sepsis | Biomed Res Int | Ye 2017 | 100 | No | Other strategies | Asia/Pacific | No | No | No | No | Other | Yes | Non-industry |
| Improved sepsis bundles in the treatment of septic shock: a prospective clinical study | Am J Emerg Med | Lu 2015 | 105 | No | Care bundles | Asia/Pacific | No | Yes | No | No | Other | No | Non-industry |
| Incidence of hypotension according to the discontinuation order of vasopressors in the management of septic shock: a prospective randomized trial (DOVSS) | Crit Care | Jeon 2018 | 40 | No | Therapeutic interventions | Asia/Pacific | No | No | Yes - clinicaltrials.gov | Yes | Other | Yes | Non-industry |
| Increasing mean arterial pressure in patients with septic shock: effects on oxygen variables and renal function | Crit Care Med | Bourgoin 2005 | 14 | No | Monitoring systems | Europe | No | No | No | No | Other | No | Non-industry |
| Induced hypothermia in patients with septic shock and respiratory failure (CASS): a randomised, controlled, open-label trial | Lancet | Itenov 2018 | 432 | Yes | Other strategies | More than one | Yes | Yes | Yes - clinicaltrials.gov | Yes | Mortality | No | Non-industry |
| Influence of angiotensin-converting enzyme inhibitor enalaprilat on endothelial-derived substances in the critically ill | Crit Care Med | Boldt 1998 | 20 | No | Therapeutic interventions | Europe | No | No | No | No | Other | Yes | Not reported |
| Influence of liberal versus conservative oxygen therapies on the hemodynamic parameters of mechanically ventilated patients with sepsis: a randomized clinical trial. | BMC anesthesiology | Ghazaly 2024 | 106 | No | Other strategies | Africa | No | No | Yes - clinicaltrials.gov | Yes | Other | No | Non-industry |
| Influence of N-acetylcysteine on indirect indicators of tissue oxygenation in septic shock patients: results from a prospective, randomized, double-blind study | Crit Care Med | Spies 1994 | 58 | No | Therapeutic interventions | North America | No | No | No | No | Other | No | Industry |
| Infusion of methylene blue in human septic shock: a pilot, randomized, controlled study | Crit Care Med | Kirov 2001 | 10 | No | Therapeutic interventions | Europe | No | No | No | No | Other | Yes | Non-industry |
| Initial evaluation of human recombinant interleukin-1 receptor antagonist in the treatment of sepsis syndrome: a randomized, open-label, placebo-controlled multicenter trial | Crit Care Med | Fisher 1994 | 99 | Yes | Therapeutic interventions | North America | No | Yes | No | No | Mortality | Yes | Industry |
| Intensive insulin therapy and pentastarch resuscitation in severe sepsis | N Engl J Med | Brunkhorst 2008 | 537 | Yes | Care bundles | Europe | No | Yes | Yes - clinicaltrials.gov | Yes | Mortality | No | Industry |
| Intensive insulin treatment improves forearm blood flow in critically ill patients: a randomized parallel design clinical trial | Crit Care | Zuran 2009 | 29 | No | Therapeutic interventions | Europe | No | No | Yes - Other | No | Other | Yes | Not reported |
| INTERSEPT: an international, multicenter, placebo-controlled trial of monoclonal antibody to human tumor necrosis factor-alpha in patients with sepsis. International Sepsis Trial Study Group | Crit Care Med | Cohen 1996 | 564 | No | Therapeutic interventions | More than one | Yes | Yes | No | No | Mortality | No | Industry |
| Intravenous administration of ulinastatin (human urinary trypsin inhibitor) in severe sepsis: a multicenter randomized controlled study | Intensive Care Med | Karnad 2014 | 122 | Yes | Therapeutic interventions | Asia/Pacific | No | Yes | Yes - Other | Yes | Mortality | Yes | Industry |
| Intravenous thiamine as an adjuvant therapy for hyperlactatemia in septic shock patients | Crit Care Shock | Harun 2019 | 65 | Yes | Therapeutic interventions | Asia/Pacific | No | No | No | No | Other | No | Non-industry |
| Intravenous vitamin C administration to patients with septic shock: a pilot randomised controlled trial | Crit Care | Rosengrave 2022 | 40 | No | Therapeutic interventions | Asia/Pacific | No | No | Yes - Other | Yes | Other | No | Non-industry |
| Intravenous Vitamin C in Adults with Sepsis in the Intensive Care Unit | N Engl J Med | Lamontagne 2022 | 862 | Yes | Therapeutic interventions | More than one | Yes | Yes | Yes - clinicaltrials.gov | Yes | Other | No | Non-industry |
| Intravenously administered interleukin-7 to reverse lymphopenia in patients with septic shock: a double-blind, randomized, placebo-controlled trial | Ann Intensive Care | Daix 2023 | 21 | No | Therapeutic interventions | More than one | Yes | Yes | Yes - clinicaltrials.gov | Yes | Other | Yes | Industry |
| Is continuous infusion ceftriaxone better than once-a-day dosing in intensive care? A randomized controlled pilot study | J Antimicrob Chemother | Roberts 2007 | 57 | No | Therapeutic interventions | Asia/Pacific | No | No | No | No | Other | No | Non-industry |
| Lactated Ringer's Versus 4% Albumin on Lactated Ringer's in Early Sepsis Therapy in Cancer Patients: a Pilot Single-Center Randomized Trial | Crit Care Med | Park 2019 | 360 | Yes | Other strategies | Latin America | No | No | Yes - clinicaltrials.gov | Yes | Mortality | No | Non-industry |
| Landiolol and Organ Failure in Patients With Septic Shock: the STRESS-L Randomized Clinical Trial | JAMA | Whitehouse 2023 | 340 | No | Therapeutic interventions | Europe | No | Yes | Yes - Other | Yes | Other | No | Non-industry |
| Landiolol for heart rate control in patients with septic shock and persistent tachycardia A multicenter Randomized Clinical Trial (Landi-SEP) | Intensive Care Med | Rehberg 2024 | 196 | No | Therapeutic interventions | Europe | Yes | Yes | Yes - Other | Yes | Other | Yes | Industry |
| Lenercept (p55 tumor necrosis factor receptor fusion protein) in severe sepsis and early septic shock: a randomized, double-blind, placebo-controlled, multicenter phase III trial with 1,342 patients | Crit Care Med | Abraham 2001 | 342 | No | Therapeutic interventions | North America | Yes | Yes | No | No | Mortality | No | Industry |
| Levosimendan for resuscitating the microcirculation in patients with septic shock: a randomized controlled study | Crit Care | Morelli 2010 | 20 | No | Therapeutic interventions | Europe | Yes | Yes | Yes - clinicaltrials.gov | Yes | Other | Yes | Not reported |
| Levosimendan for the prevention of acute organ dysfunction in sepsis | N Engl J Med | Gordon 2016 | 515 | Yes | Therapeutic interventions | Europe | No | Yes | Yes - Other | Yes | Other | No | Non-industry |
| Levosimendan Versus Dobutamine in Myocardial Injury Patients with Septic Shock: a Randomized Controlled Trial | Med Sci Monit | Meng 2016 | 38 | No | Therapeutic interventions | Asia/Pacific | No | No | No | No | Other | Yes | Non-industry |
| Liberal Versus Restrictive Transfusion Strategy in Critically Ill Oncologic Patients: the Transfusion Requirements in Critically Ill Oncologic Patients Randomized Controlled Trial | Crit Care Med | Bergamin 2017 | 300 | Yes | Other strategies | Latin America | No | No | Yes - clinicaltrials.gov | Yes | Mortality | No | Not reported |
| Linezolid pharmacokinetic/pharmacodynamic profile in critically ill septic patients: intermittent versus continuous infusion | Int J Antimicrob Agents | Adembri 2008 | 18 | No | Therapeutic interventions | Europe | No | No | No | No | Other | Yes | Non-industry |
| Long chain versus medium chain lipids in patients with ARDS: effects on pulmonary haemodynamics and gas exchange | Intensive Care Med | Smirniotis 1998 | 11 | No | Other strategies | Europe | No | No | No | No | Other | Yes | Not reported |
| Low central venous pressure is not associated with low perfusion event in the setting of septic shock: a randomized controlled trial | Crit Care Shock | George 2022 | 43 | No | Monitoring systems | Asia/Pacific | No | No | Yes - clinicaltrials.gov | Yes | Other | No | Not reported |
| Low-dose hydrocortisone during severe sepsis: effects on microalbuminuria | Crit Care Med | Rinaldi 2006 | 40 | No | Therapeutic interventions | Europe | No | No | No | No | Other | Yes | Not reported |
| Low-dose hydrocortisone improves shock reversal and reduces cytokine levels in early hyperdynamic septic shock | Crit Care Med | Oppert 2005 | 41 | No | Therapeutic interventions | Europe | No | No | No | No | Other | Yes | Not reported |
| Low-dose hydrocortisone in patients with cirrhosis and septic shock: a randomized controlled trial | CMAJ | Arabi 2010 | 75 | No | Therapeutic interventions | Asia/Pacific | No | No | Yes - Other | Yes | Mortality | No | Non-industry |
| Low-dose hydrocortisone treatment for patients with septic shock: a pilot study comparing 3days with 7days | Respirology | Huh 2011 | 130 | Yes | Therapeutic interventions | Asia/Pacific | No | No | No | No | Mortality | No | Not reported |
| Low-dose vasopressin in the treatment of vasodilatory septic shock | J Trauma | Malay 1999 | 10 | No | Therapeutic interventions | More than one | Yes | Yes | Yes - clinicaltrials.gov | Yes | Mortality | No | Industry |
| Lower versus higher hemoglobin threshold for transfusion in septic shock | N Engl J Med | Holst 2014 | 998 | Yes | Other strategies | Europe | Yes | Yes | Yes - clinicaltrials.gov | Yes | Mortality | No | Non-industry |
| LY315920NA/S-5920, a selective inhibitor of group IIA secretory phospholipase A2, fails to improve clinical outcome for patients with severe sepsis | Crit Care Med | Zeiher 2005 | 373 | No | Therapeutic interventions | More than one | Yes | Yes | No | No | Mortality | No | Industry |
| Mega-dose sodium ascorbate: a pilot, single-dose, physiological effect, double-blind, randomized, controlled trial | Crit Care | Yanase 2023 | 30 | Yes | Therapeutic interventions | Asia/Pacific | No | No | Yes - Other | Yes | Other | No | Non-industry |
| Meropenem dosing in critically ill patients with sepsis and without renal dysfunction: intermittent bolus versus continuous administration? Monte Carlo dosing simulations and subcutaneous tissue distribution | J Antimicrob Chemother | Roberts 2009 | 10 | No | Therapeutic interventions | Asia/Pacific | No | No | No | No | Other | Yes | Non-industry |
| Metabolic effects of a D-beta-hydroxybutyrate infusion in septic patients: inhibition of lipolysis and glucose production but not leucine oxidation | Crit Care Med | Beylot 1994 | 12 | No | Therapeutic interventions | Europe | No | No | No | No | Other | No | Non-industry |
| Metabolic resuscitation therapy in critically ill patients with sepsis and septic shock: a pilot prospective randomized controlled trial | Open Medicine | Feng 2023 | 136 | Yes | Other strategies | Asia/Pacific | No | Yes | Yes - Other | Yes | Mortality | Yes | Non-industry |
| Metabolic resuscitation using hydrocortisone ascorbic acid thiamine: Do individual components influence reversal of shock independently? | Indian J Crit Care Med | Reddy 2020 | 27 | No | Therapeutic interventions | Asia/Pacific | No | No | No | No | Other | No | Non-industry |
| Microcirculation properties of 20 % albumin in sepsis; a randomised controlled trial. | J Crit Care | Cusack 2025 | 103 | Yes | Other strategies | Europe | No | No | Yes - clinicaltrials.gov | Yes | Other | Yes | Industry |
| Microcirculatory effects of the transfusion of leukodepleted or non-leukodepleted red blood cells in patients with sepsis: a pilot study | Crit Care | Donati 2014 | 20 | No | Other strategies | Europe | No | No | Yes - clinicaltrials.gov | Yes | Other | Yes | Non-industry |
| Midodrine improves clinical and economic outcomes in patients with septic shock: a randomized controlled clinical trial | Irish Journal of Medical Science | Adly 2022 | 60 | Yes | Therapeutic interventions | Africa | No | No | Yes - clinicaltrials.gov | No | Other | Yes | Non-industry |
| Mixed fibers diet in surgical ICU septic patients | Crit Care | Chittawatanarat 2010 | 17 | No | Other strategies | Asia/Pacific | No | No | No | No | Other | Yes | Industry |
| Multicenter evaluation of a human monoclonal antibody to Enterobacteriaceae common antigen in patients with Gram-negative sepsis | Crit Care Med | Albertson 2003 | 826 | Yes | Therapeutic interventions | North America | No | Yes | Yes - Other | No | Mortality | Yes | Industry |
| Multicenter, double-blind, placebo-controlled study of the use of filgrastim in patients hospitalized with pneumonia and severe sepsis | Crit Care Med | Root 2003 | 701 | Yes | Therapeutic interventions | More than one | Yes | Yes | No | No | Mortality | No | Industry |
| Multiple-center, randomized, placebo-controlled, double-blind study of the nitric oxide synthase inhibitor 546C88: effect on survival in patients with septic shock | Crit Care Med | Lopez 2004 | 797 | Yes | Therapeutic interventions | More than one | Yes | Yes | No | Yes | Mortality | No | Industry |
| Muscle wasting and function after muscle activation and early protocol-based physiotherapy: an explorative trial | Journal of Cachexia, Sarcopenia and Muscle | Wollersheim 2019 | 50 | Yes | Other strategies | Europe | No | No | Yes - Other | Yes | Other | No | Non-industry |
| Muscle weakness in septic patients requiring mechanical ventilation: protective effect of transcutaneous neuromuscular electrical stimulation | J Crit Care | Rodriguez 2012 | 16 | No | Other strategies | Latin America | No | No | No | No | Other | Yes | Non-industry |
| N-acetyl-L-cysteine depresses cardiac performance in patients with septic shock | Crit Care Med | Peake 1996 | 20 | Yes | Therapeutic interventions | Europe | No | No | No | No | Other | Yes | Not reported |
| N-acetylcysteine increases liver blood flow and improves liver function in septic shock patients: results of a prospective, randomized, double-blind study | Crit Care Med | Rank 2000 | 30 | No | Therapeutic interventions | Europe | No | Yes | No | No | Other | Yes | Not reported |
| N-acetylcysteine reduces respiratory burst but augments neutrophil phagocytosis in intensive care unit patients | Crit Care Med | Heller 2001 | 15 | No | Therapeutic interventions | Europe | No | Yes | No | No | Other | Yes | Non-industry |
| Nangibotide in patients with septic shock: a Phase 2a randomized controlled clinical trial | Intensive Care Med | Francois 2020 | 49 | No | Therapeutic interventions | Europe | Yes | Yes | Yes - clinicaltrials.gov | Yes | Other | No | Industry |
| Nano-curcumin supplementation in critically ill patients with sepsis: a randomized clinical trial investigating the inflammatory biomarkers, oxidative stress indices, endothelial function, clinical outcomes and nutritional status | Food Funct | Karimi 2022 | 40 | No | Therapeutic interventions | Asia/Pacific | No | Yes | Yes - Other | Yes | Other | Yes | Non-industry |
| Neuromuscular electrical stimulation acutely mobilizes endothelial progenitor cells in critically ill patients with sepsis | Ann Intensive Care | Stefanou 2016 | 32 | Yes | Other strategies | Europe | No | Yes | No | No | Other | Yes | Non-industry |
| Neuromuscular Electrical Stimulator as a Protective Treatment against Intensive Care Unit Muscle Wasting in Sepsis/Septic Shock Patients | J Coll Physicians Surg Pak | Cebeci 2022 | 40 | No | Other strategies | Europe | No | No | Yes - clinicaltrials.gov | Yes | Other | Yes | Not reported |
| Nocturnal urine 6-hydroxy sulfate melatonin is associated with the outcome of subjects with sepsis | Emerg Crit Care Med | Li 2022 | 36 | Yes | Monitoring systems | Asia/Pacific | No | No | Yes - clinicaltrials.gov | Yes | Other | No | Non-industry |
| Norepinephrine in low to moderate doses may not increase luminal concentrations of L-lactate in the gut in patients with septic shock | Acta Anaesthesiol Scand | Ibsen 2007 | 8 | No | Therapeutic interventions | Europe | No | No | No | No | Other | No | Not reported |
| Norepinephrine or dopamine for the treatment of hyperdynamic septic shock? | Chest | Martin 1993 | 16 | No | Therapeutic interventions | Europe | No | No | No | No | Other | Yes | Not reported |
| Norepinephrine plus dobutamine versus epinephrine alone for management of septic shock: a randomised trial | Lancet | Annane 2007 | 330 | No | Therapeutic interventions | Europe | No | Yes | Yes - clinicaltrials.gov | Yes | Mortality | No | Non-industry |
| Norepinephrine supplemented with dobutamine or epinephrine for the cardiovascular support of patients with septic shock | Indian J Crit Care Med | Mahmoud 2012 | 60 | No | Therapeutic interventions | Africa | No | No | No | No | Other | Yes | Non-industry |
| Norepinephrine titration in patients with sepsis-induced encephalopathy: cerebral pulsatility index compared to mean arterial pressure guided protocol: randomized controlled trial. | BMC anesthesiology | Salem 2025 | 112 | No | Therapeutic interventions | Africa | No | No | Yes - clinicaltrials.gov | Yes | Mortality | No | Non-industry |
| Norepinephrine weaning in septic shock patients by closed loop control based on fuzzy logic | Crit Care | Merouani 2008 | 39 | Yes | Monitoring systems | Europe | No | No | Yes - clinicaltrials.gov | Yes | Other | Yes | Non-industry |
| Nursing Based on Humanistic Care Concept for Continuous Blood Purification for Patients with Severe Sepsis in the Intensive Care Unit. | Alternative therapies in health and medicine | Fan 2024 | 80 | No | Other strategies | Asia/Pacific | No | No | No | No | Other | Yes | Non-industry |
| Open randomized phase II trial of an extracorporeal endotoxin adsorber in suspected Gram-negative sepsis | Crit Care Med | Reinhart 2004 | 76 | No | Other strategies | Europe | Yes | Yes | No | Yes | Other | No | Industry |
| Optimizing left ventricular-arterial coupling during the initial resuscitation in septic shock - a pilot prospective randomized study | BMC Anesthesiol | Zhou 2022 | 83 | Yes | Monitoring systems | Asia/Pacific | No | No | Yes - Other | Yes | Mortality | No | Non-industry |
| Oral Midodrine Administration During the First 24 Hours of Sepsis to Reduce the Need of Vasoactive Agents: Placebo-Controlled Feasibility Clinical Trial. | Crit Care Explor | Lal 2021 | 32 | No | Therapeutic interventions | More than one | Yes | Yes | Yes - clinicaltrials.gov | Yes | Other | No | Non-industry |
| Outcome of ulinastatin vs metabolic resuscitation using ascorbic acid, thiamine and glucocorticoid in early treatment of sepsis-a randomised controlled trial | Journal of Clinical and Diagnostic Research | Yadav 2021 | 60 | No | Therapeutic interventions | Asia/Pacific | No | No | No | No | Other | Yes | Not reported |
| Outcomes of High-Dose Versus Low-Dose Vitamin D on Prognosis of Sepsis Requiring Mechanical Ventilation: A Randomized Controlled Trial. | J Intensive Care Med | Ashoor 2024 | 80 | Yes | Therapeutic interventions | Africa | No | No | Yes - clinicaltrials.gov | Yes | Other | Yes | Non-industry |
| Outcomes of Metabolic Resuscitation Using Ascorbic Acid, Thiamine, and Glucocorticoids in the Early Treatment of Sepsis: the ORANGES Trial | Chest | Iglesias 2020 | 137 | Yes | Therapeutic interventions | North America | No | Yes | Yes - clinicaltrials.gov | Yes | Other | Yes | Non-industry |
| Oxygen delivery, oxygen consumption, and gastric intramucosal pH are not improved by a computer-controlled, closed-loop, vecuronium infusion in severe sepsis and septic shock | Crit Care Med | Freebairn 1997 | 18 | No | Monitoring systems | Europe | No | No | No | No | Other | No | Not reported |
| P(v-a)CO2/C(a-v)O2-directed resuscitation does not improve prognosis compared with SvO2 in severe sepsis and septic shock: a prospective multicenter randomized controlled clinical study | J Crit Care | Su 2018 | 228 | Yes | Care bundles | Asia/Pacific | No | Yes | Yes - clinicaltrials.gov | Yes | Mortality | No | Not reported |
| Pancreatic enzyme replacement therapy can improve infection level nutrition condition and prognosis of patients with sepsis | Food and Nutrition Research | Zhao 2025 | 25 | No | Other strategies | Asia/Pacific | No | No | No | No | Other | Yes | Non-industry |
| Parenteral administration of different amounts of branch-chain amino acids in septic patients: clinical and metabolic aspects | Crit Care Med | Garcia-de-Lorenzo 1997 | 25 | No | Other strategies | Europe | No | Yes | No | No | Other | Yes | Industry |
| Parenteral nutrition with fish oil modulates cytokine response in patients with sepsis | Am J Respir Crit Care Med | Mayer 2003 | 21 | No | Other strategies | Europe | No | Yes | No | No | Other | Yes | Industry |
| Pentoxifylline and oxygen consumption in severe sepsis--a preliminary report | Acta Anaesthesiol Scand | Castanon-Gonzalez 1995 | 13 | No | Therapeutic interventions | Latin America | No | No | No | No | Other | No | Not reported |
| Pharmacokinetic and Pharmacodynamic Efficacies of Continuous versus Intermittent Administration of Meropenem in Patients with Severe Sepsis and Septic Shock: a Prospective Randomized Pilot Study | Chin Med J | Zhao 2017 | 25 | No | Therapeutic interventions | Asia/Pacific | No | No | No | No | Other | No | Non-industry |
| Pharmacokinetic evaluation of meropenem and imipenem in critically ill patients with sepsis | Clin Pharmacokinet | Novelli 2005 | 20 | No | Therapeutic interventions | Europe | No | No | No | No | Other | Yes | Not reported |
| Pharmacokinetics and Pharmacodynamics of Linezolid in Patients With Sepsis Receiving Continuous Venovenous Hemofiltration and Extended Daily Hemofiltration | J Infect Dis | Zheng 2020 | 20 | No | Therapeutic interventions | Asia/Pacific | No | Yes | No | No | Other | No | Non-industry |
| Pharmacokinetics of piperacillin in critically ill patients receiving continuous venovenous haemofiltration: a randomised controlled trial of continuous infusion versus intermittent bolus administration | Int J Antimicrob Agents | Jamal 2015 | 16 | No | Therapeutic interventions | Asia/Pacific | Yes | No | No | No | Other | Yes | Non-industry |
| Phase 2 trial of eritoran tetrasodium (E5564), a toll-like receptor 4 antagonist, in patients with severe sepsis | Crit Care Med | Tidswell 2010 | 300 | No | Therapeutic interventions | North America | Yes | Yes | Yes - Other | No | Mortality | No | Industry |
| Phase 3 Pilot Randomized Controlled Trial Comparing Early Trophic Enteral Nutrition With "No Enteral Nutrition" in Mechanically Ventilated Patients With Septic Shock | J Parenter Enteral Nutr | Patel 2020 | 31 | No | Therapeutic interventions | North America | No | No | Yes - clinicaltrials.gov | Yes | Other | Yes | Non-industry |
| Phase I safety trial of intravenous ascorbic acid in patients with severe sepsis | Journal of Translational Medicine | Fowler 2014 | 24 | Yes | Therapeutic interventions | North America | No | No | Yes - clinicaltrials.gov | Yes | Other | Yes | Non-industry |
| Phase II multicenter clinical study of the platelet-activating factor receptor antagonist BB-882 in the treatment of sepsis | Crit Care Med | Vincent 2000 | 75 | No | Therapeutic interventions | Europe | Yes | Yes | No | No | Mortality | No | Industry |
| Phase-3 trial of recombinant human alkaline phosphatase for patients with sepsis-associated acute kidney injury (REVIVAL) | Intensive Care Med | Pickkers 2024 | 330 | No | Therapeutic interventions | More than one | Yes | Yes | Yes - clinicaltrials.gov | Yes | Mortality | No | Industry |
| Phenylephrine versus norepinephrine for initial hemodynamic support of patients with septic shock: a randomized, controlled trial | Crit Care | Morelli 2008 | 32 | No | Therapeutic interventions | Europe | Yes | Yes | Yes - clinicaltrials.gov | Yes | Other | No | Non-industry |
| Physiological-dose steroid therapy in sepsis | Crit Care | Yildiz 2002 | 20 | No | Therapeutic interventions | Europe | No | No | Yes - Other | No | Mortality | No | Not reported |
| Pilot study on the effects of high cutoff hemofiltration on the need for norepinephrine in septic patients with acute renal failure | Crit Care Med | Morgera 2006 | 20 | No | Other strategies | Europe | No | No | No | No | Other | Yes | Not reported |
| Piperacillin penetration into tissue of critically ill patients with sepsis--bolus versus continuous administration? | Crit Care Med | Roberts 2009 | 13 | No | Therapeutic interventions | Asia/Pacific | No | No | No | No | Other | Yes | Non-industry |
| Plasma as endothelial rescue in septic shock: A randomized, phase 2a pilot trial. | Transfusion | Clausen 2024 | 44 | No | Other strategies | Europe | No | No | Yes - clinicaltrials.gov | Yes | Other | No | Industry |
| Platelet-activating factor receptor antagonist BN 52021 in the treatment of severe sepsis: A randomized, double-blind, placebo-controlled, multicenter clinical trial | Crit Care Med | Dhainaut 1994 | 67 | No | Therapeutic interventions | Europe | No | Yes | No | No | Mortality | No | Industry |
| Polymyxin B therapy based on therapeutic drug monitoring in carbapenem-resistant organisms sepsis: the PMB-CROS randomized clinical trial | Crit Care | Liu 2023 | 311 | Yes | Therapeutic interventions | Asia/Pacific | No | Yes | Yes - Other | Yes | Other | No | Non-industry |
| Polymyxin-B hemoperfusion inactivates circulating proapoptotic factors | Intensive Care Med | Cantaluppi 2008 | 16 | No | Other strategies | Europe | No | Yes | No | No | Other | Yes | Non-industry |
| Positive effect of septimeb√¢¬Ñ¬¢ on mortality rate in severe sepsis: a novel non antibiotic strategy | Daru | Eslami 2012 | 29 | No | Therapeutic interventions | Asia/Pacific | No | Yes | Yes - Other | Yes | Mortality | No | Non-industry |
| Pre-emptive hydrocortisone therapy in early septic shock: a double-blind, allocation-concealed, pilot randomized controlled trial. | Daru | Emami 2025 | 40 | No | Therapeutic interventions | Asia/Pacific | No | No | Yes - Other | Yes | Other | Yes | Non-industry |
| Preload dependence indices to titrate volume expansion during septic shock: a randomized controlled trial | Crit Care | Richard 2015 | 30 | No | Monitoring systems | Europe | No | No | Yes - clinicaltrials.gov | Yes | Other | No | Non-industry |
| Procalcitonin (PCT)-guided algorithm reduces length of antibiotic treatment in surgical intensive care patients with severe sepsis: results of a prospective randomized study | Langenbecks Arch Surg | Schroeder 2009 | 14 | No | Therapeutic interventions | Europe | No | No | No | No | Other | Yes | Not reported |
| Procalcitonin algorithm in critically ill adults with undifferentiated infection or suspected sepsis. A randomized controlled trial | Am J Respir Crit Care Med | Shehabi 2014 | 400 | Yes | Monitoring systems | Asia/Pacific | No | Yes | Yes - Other | Yes | Other | No | Non-industry |
| Procalcitonin to guide duration of antibiotic therapy in intensive care patients: a randomized prospective controlled trial | Crit Care | Hochreiter 2009 | 57 | No | Monitoring systems | Europe | No | No | Yes - Other | Yes | Other | Yes | Industry |
| Procalcitonin versus C-reactive protein for guiding antibiotic therapy in sepsis: a randomized trial | Crit Care Med | Oliveira 2013 | 97 | Yes | Monitoring systems | Latin America | No | Yes | Yes - clinicaltrials.gov | Yes | Other | No | Non-industry |
| Procalcitonin-Guided Management and Duration of Antibiotic Therapy in Critically Ill Cancer Patients With Sepsis (Pro-Can Study): A Randomized Controlled Trial. | Crit Care Explor | Nazer 2024 | 77 | No | Monitoring systems | Asia/Pacific | No | No | Yes - clinicaltrials.gov | Yes | Other | No | Non-industry |
| Procalcitonin-Guided Treatment on Duration of Antibiotic Therapy and Cost in Septic Patients (PRODA): a Multi-Center Randomized Controlled Trial | J Korean Med Sci | Jeon 2019 | 52 | Yes | Monitoring systems | Asia/Pacific | No | Yes | Yes - clinicaltrials.gov | Yes | Other | Yes | Non-industry |
| Prophylactic fenoldopam for renal protection in sepsis: a randomized, double-blind, placebo-controlled pilot trial | Crit Care Med | Morelli 2005 | 150 | No | Therapeutic interventions | Europe | No | Yes | No | No | Other | Yes | Non-industry |
| Prophylactic heparin in patients with severe sepsis treated with drotrecogin alfa (activated) | Am J Respir Crit Care Med | Levi 2007 | 1935 | Yes | Therapeutic interventions | More than one | Yes | Yes | Yes - clinicaltrials.gov | Yes | Other | No | Industry |
| Propofol increases preload dependency in septic shock patients | J Surg Res | Yu 2015 | 43 | No | Therapeutic interventions | Asia/Pacific | No | No | Yes - clinicaltrials.gov | Yes | Other | Yes | Non-industry |
| Prospective evaluation of the efficacy, safety, and optimal biomarker enrichment strategy for nangibotide, a TREM-1 inhibitor, in patients with septic shock (ASTONISH): a double-blind, randomised, controlled, phase 2b trial | Lancet Respir Med | Francois 2023 | 402 | No | Therapeutic interventions | More than one | Yes | Yes | Yes - clinicaltrials.gov | Yes | Other | No | Industry |
| Protective effect of rhubarb combined with ulinastatin for patients with sepsis | Medicine (Baltimore) | Meng 2020 | 75 | No | Therapeutic interventions | Asia/Pacific | No | No | No | No | Other | Yes | Not reported |
| Protective effect of Xuebijing injection on myocardial injury in patients with sepsis: a randomized clinical trial | Journal of Traditional Chinese Medicine | Zhang 2016 | 72 | Yes | Therapeutic interventions | Asia/Pacific | No | Yes | No | No | Other | Yes | Non-industry |
| Protein C zymogen in severe sepsis: a double-blinded, placebo-controlled, randomized study | Intensive Care Med | Pappalardo 2016 | 37 | Yes | Therapeutic interventions | Europe | No | No | Yes - clinicaltrials.gov | Yes | Other | No | Non-industry |
| Protocolized reduction of non-resuscitation fluids versus usual care in septic shock patients (REDUSE): a randomized multicentre feasibility trial. | Crit Care | Linden 2024 | 134 | No | Other strategies | Europe | No | Yes | Yes - clinicaltrials.gov | Yes | Other | Yes | Non-industry |
| Randomized Clinical Trial of Antioxidant Therapy Patients with Septic Shock and Organ Dysfunction in the ICU: SOFA Score Reduction by Improvement of the Enzymatic and Non-Enzymatic Antioxidant System | Cells | Aisa-Alvarez 2023 | 131 | Yes | Therapeutic interventions | Latin America | No | Yes | Yes - clinicaltrials.gov | No | Other | Yes | Non-industry |
| Randomized controlled clinical trial evaluating multiplex polymerase chain reaction for pathogen identification and therapy adaptation in critical care patients with pulmonary or abdominal sepsis | J Int Med Res | Tafelski 2015 | 37 | No | Therapeutic interventions | Europe | No | Yes | Yes - Other | Yes | Other | Yes | Industry |
| Randomized controlled trial of calcitriol in severe sepsis | Am J Respir Crit Care Med | Leaf 2014 | 67 | Yes | Therapeutic interventions | North America | No | Yes | Yes - clinicaltrials.gov | Yes | Other | No | Non-industry |
| Randomized trial evaluating serial protein C levels in severe sepsis patients treated with variable doses of drotrecogin alfa (activated) | Crit Care | Shorr 2010 | 433 | Yes | Monitoring systems | More than one | Yes | Yes | Yes - clinicaltrials.gov | Yes | Other | Yes | Industry |
| Randomized, double blind, placebo-controlled trial of fish-oil-based lipid emulsion infusion for treatment of critically ill patients with severe sepsis | Asian J Surg | Khor 2011 | 28 | Yes | Other strategies | Asia/Pacific | No | No | Yes - clinicaltrials.gov | Yes | Other | Yes | Not reported |
| Randomized, double-blind, placebo-controlled crossover pilot study of a potassium channel blocker in patients with septic shock | Crit Care Med | Warrillow 2006 | 10 | No | Therapeutic interventions | Asia/Pacific | No | No | No | No | Other | No | Non-industry |
| Randomized, double-blind, placebo-controlled trial of granulocyte colony-stimulating factor in patients with septic shock | Crit Care Med | Stephens 2008 | 164 | Yes | Therapeutic interventions | Asia/Pacific | No | No | Yes - Other | Yes | Mortality | No | Non-industry |
| Randomized, placebo-controlled trial of acetaminophen for the reduction of oxidative injury in severe sepsis: the Acetaminophen for the Reduction of Oxidative Injury in Severe Sepsis trial | Crit Care Med | Janz 2015 | 22 | No | Therapeutic interventions | North America | No | No | Yes - clinicaltrials.gov | Yes | Other | No | Non-industry |
| Randomized, placebo-controlled trial of the anti-tumor necrosis factor antibody fragment afelimomab in hyperinflammatory response during severe sepsis: the RAMSES Study | Crit Care Med | Reinhart 2001 | 498 | No | Therapeutic interventions | More than one | Yes | Yes | No | Yes | Mortality | No | Industry |
| Ranitidine is unable to maintain gastric pH levels above 4 in septic patients | J Crit Care | TerziCoelho 2009 | 20 | No | Therapeutic interventions | Latin America | No | No | No | No | Other | Yes | Non-industry |
| Recombinant human activated protein C for adults with septic shock: a randomized controlled trial | Am J Respir Crit Care Med | Annane 2013 | 411 | No | Therapeutic interventions | Europe | No | Yes | Yes - clinicaltrials.gov | Yes | Mortality | No | Non-industry |
| Recombinant human interleukin 1 receptor antagonist in the treatment of patients with sepsis syndrome. Results from a randomized, double-blind, placebo-controlled trial. Phase III rhIL-1ra Sepsis Syndrome Study Group | JAMA | Fisher 1994 | 893 | Yes | Therapeutic interventions | More than one | Yes | Yes | No | No | Mortality | No | Industry |
| Recombinant human platelet-activating factor acetylhydrolase for treatment of severe sepsis: results of a phase III, multicenter, randomized, double-blind, placebo-controlled, clinical trial | Crit Care Med | Opal 2004 | 522 | No | Therapeutic interventions | More than one | Yes | Yes | No | No | Mortality | No | Industry |
| Recombinant platelet-activating factor acetylhydrolase to prevent acute respiratory distress syndrome and mortality in severe sepsis: phase IIb, multicenter, randomized, placebo-controlled, clinical trial | Crit Care Med | Schuster 2003 | 127 | No | Therapeutic interventions | North America | No | Yes | No | No | Other | No | Industry |
| Red blood cell transfusion does not increase oxygen consumption in critically ill septic patients | Crit Care | Fernandes 2001 | 15 | No | Other strategies | Latin America | No | No | No | No | Other | No | Not reported |
| Remote ischemic conditioning in septic shock: the RECO-Sepsis randomized clinical trial | Intensive Care Med | Cour 2022 | 178 | Yes | Other strategies | Europe | No | Yes | Yes - clinicaltrials.gov | Yes | Other | No | Non-industry |
| Removal of humoral mediators and the effect on the survival of septic patients by hemoperfusion with neutral microporous resin column | Ther Apher Dial | Huang 2010 | 24 | No | Other strategies | Asia/Pacific | No | No | No | No | Other | Yes | Not reported |
| Renal protective effect and clinical analysis of vitamin B6 in patients with sepsis | Shock | Wang 2024 | 128 | No | Therapeutic interventions | Asia/Pacific | No | Yes | Yes - clinicaltrials.gov | Yes | Other | Yes | Non-industry |
| Restricting volumes of resuscitation fluid in adults with septic shock after initial management: the CLASSIC randomised, parallel-group, multicentre feasibility trial | Intensive Care Med | Hjortrup 2016 | 151 | Yes | Other strategies | Europe | Yes | Yes | Yes - clinicaltrials.gov | Yes | Other | Yes | Non-industry |
| Restriction of Intravenous Fluid in ICU Patients with Septic Shock | N Engl J Med | Meyhoff 2022 | 1531 | Yes | Other strategies | More than one | Yes | Yes | Yes - clinicaltrials.gov | Yes | Mortality | No | Non-industry |
| Results of a pilot study on the effects of propofol and dexmedetomidine on inflammatory responses and intraabdominal pressure in severe sepsis | J Clin Anesth | Tasdogan 2009 | 20 | No | Therapeutic interventions | Europe | No | No | No | No | Other | Yes | Non-industry |
| Reversal of late septic shock with supraphysiologic doses of hydrocortisone | Crit Care Med | Bollaert 1998 | 41 | No | Therapeutic interventions | North America | No | No | No | No | Other | Yes | Not reported |
| Right dose, right now: bedside, real-time, data-driven, and personalised antibiotic dosing in critically ill patients with sepsis or septic shock-a two-centre randomised clinical trial | Crit Care | Roggeveen 2022 | 349 | Yes | Monitoring systems | Europe | No | Yes | Yes - Other | Yes | Other | Yes | Non-industry |
| Role of dexmedetomidine in modifying immune paralysis in patients with septic shock: randomized controlled trial | Intensive Care Medicine Experimental | Elayashy 2023 | 12 | No | Therapeutic interventions | Africa | No | No | Yes - clinicaltrials.gov | Yes | Other | No | Non-industry |
| Role of edaravone in managemant of septic peritonitis | J Anaesthesiol Clin Pharmacol | Elbaradey 2016 | | No | Therapeutic interventions | Africa | No | No | No | No | Other | Yes | Non-industry |
| Role of IV Vitamin C in Treatment and Prognosis of Sepsis | Journal of Cardiovascular Disease Research | Shakya 2024 | 76 | No | Therapeutic interventions | Asia/Pacific | No | No | No | No | Other | Yes | Not reported |
| Roles of small-dose recombinant human brain natriuretic peptide without bolus in Chinese older patients with septic cardiac dysfunction | European Geriatric Medicine | Zhang 2019 | 250 | Yes | Therapeutic interventions | Asia/Pacific | No | No | No | No | Mortality | No | Not reported |
| Rosuvastatin for sepsis-associated acute respiratory distress syndrome | N Engl J Med | Lung 2014 | 1000 | No | Therapeutic interventions | North America | No | Yes | Yes - clinicaltrials.gov | Yes | Mortality | No | Industry |
| Safety and efficacy of affinity-purified, anti-tumor necrosis factor-alpha, ovine fab for injection (CytoFab) in severe sepsis | Crit Care Med | Rice 2006 | 81 | Yes | Therapeutic interventions | North America | Yes | Yes | Yes - Other | No | Other | No | Industry |
| Safety and Efficacy of Vitamin C, Vitamin B1, and Hydrocortisone in clinical outcome of septic shock receiving standard care: a quasi experimental randomized open label two arm parallel group study | European Journal of Molecular and Clinical Medicine | Raghu 2021 | 240 | No | Therapeutic interventions | Asia/Pacific | No | Yes | Yes - Other | Yes | Mortality | No | Non-industry |
| Safety and tolerability of non-neutralizing adrenomedullin antibody adrecizumab (HAM8101) in septic shock patients: the AdrenOSS-2 phase 2a biomarker-guided trial | Intensive Care Med | Laterre 2021 | 301 | Yes | Therapeutic interventions | Europe | Yes | Yes | Yes - clinicaltrials.gov | Yes | Other | Yes | Industry |
| Safety and vasopressor effect of rosuvastatin in septic patients | Egyptian Journal of Anaesthesia | ElGendy 2014 | 108 | No | Therapeutic interventions | Africa | No | No | No | No | Other | Yes | Non-industry |
| Safety of megadose meropenem in the empirical treatment of nosocomial sepsis: a pilot randomized clinical trial | Future Microbiology | Salehi 2023 | 30 | Yes | Therapeutic interventions | Asia/Pacific | No | No | No | No | Other | Yes | Not reported |
| Safety, tolerability, pharmacokinetics, and efficacy of kukoamine B in patients with sepsis: a randomized phase IIa trial | J Crit Care | Hu 2023 | 44 | Yes | Therapeutic interventions | Asia/Pacific | No | Yes | Yes - clinicaltrials.gov | Yes | Other | Yes | Industry |
| Score-based immunoglobulin G therapy of patients with sepsis: the SBITS study | Crit Care Med | Werdan 2007 | 624 | Yes | Therapeutic interventions | Europe | No | Yes | Yes - Other | Yes | Mortality | No | Industry |
| Selepressin, a novel selective vasopressin V1A agonist, is an effective substitute for norepinephrine in a phase IIa randomized, placebo-controlled trial in septic shock patients | Crit Care | Russell 2017 | 53 | Yes | Therapeutic interventions | More than one | Yes | Yes | Yes - clinicaltrials.gov | Yes | Other | Yes | Industry |
| Serum IL-6 and IL-1-ra with sequential organ failure assessment scores in septic patients receiving high-volume haemofiltration and continuous venovenous haemofiltration | NEPHROLOGY | Ghani 2006 | 33 | Yes | Therapeutic interventions | Asia/Pacific | No | No | No | No | Other | Yes | Industry |
| Serum procalcitonin as a biomarker to determine the duration of antibiotic therapy in adult patients with sepsis and septic shock in intensive care units: a prospective study | Indian J Crit Care Med | Vishalashi 2021 | 90 | No | Monitoring systems | Asia/Pacific | No | No | No | No | Other | Yes | Non-industry |
| Shenfu injection for improving cellular immunity and clinical outcome in patients with sepsis or septic shock | Am J Emerg Med | Zhang 2017 | 157 | No | Therapeutic interventions | Asia/Pacific | No | No | No | No | Other | Yes | Non-industry |
| Single-blinded, randomized, and controlled clinical trial evaluating the effects of Omega-3 fatty acids among septic patients with intestinal dysfunction: a pilot study | Exp Ther Med | Chen 2017 | 48 | No | Other strategies | Asia/Pacific | No | No | No | No | Mortality | Yes | Industry |
| Soluble fiber reduces the incidence of diarrhea in septic patients receiving total enteral nutrition: a prospective, double-blind, randomized, and controlled trial | Clin Nutr | Spapen 2001 | 25 | No | Other strategies | Europe | No | No | No | No | Other | Yes | Industry |
| Solumedrol Treatment for Severe Sepsis in Humans with a Blunted Adrenocorticotropic Hormone-Cortisol Response: a Prospective Randomized Double-Blind Placebo-Controlled Pilot Clinical Trial | J Intensive Care Med | Birudaraju 2022 | 54 | No | Therapeutic interventions | North America | No | No | No | No | Other | Yes | Industry |
| StO2 guided early resuscitation in subjects with severe sepsis or septic shock: a pilot randomised trial | JOURNAL OF CLINICAL MONITORING AND COMPUTING | Nardi 2013 | 30 | No | Monitoring systems | Europe | No | No | Yes - clinicaltrials.gov | Yes | Other | No | Not reported |
| Stress doses of hydrocortisone in septic shock: beneficial effects on opsonization-dependent neutrophil functions | Intensive Care Med | Kaufmann 2008 | 15 | No | Therapeutic interventions | Europe | No | No | No | No | Other | Yes | Not reported |
| Stress doses of hydrocortisone reverse hyperdynamic septic shock: a prospective, randomized, double-blind, single-center study | Crit Care Med | Briegel 1999 | 20 | No | Therapeutic interventions | Europe | No | No | No | No | Other | Yes | Not reported |
| Talactoferrin in Severe Sepsis: results From the Phase II/III Oral tAlactoferrin in Severe sepsIS Trial | Crit Care Med | Vincent 2015 | 305 | No | Therapeutic interventions | More than one | Yes | Yes | Yes - clinicaltrials.gov | Yes | Mortality | No | Industry |
| Targeted Fluid Minimization Following Initial Resuscitation in Septic Shock: a Pilot Study | Chest | Chen 2015 | 82 | Yes | Monitoring systems | North America | No | No | Yes - clinicaltrials.gov | Yes | Other | No | Non-industry |
| Targeting matrix metalloproteinases with intravenous doxycycline in severe sepsis--A randomised placebo-controlled pilot trial | Pharmacol Res | Nukarinen 2015 | 24 | Yes | Therapeutic interventions | Europe | No | No | Yes - Other | Yes | Other | No | Non-industry |
| Targeting skeletal muscle tissue oxygenation (StO2) in adults with severe sepsis and septic shock: a randomised controlled trial (OTO-StS Study) | BMJ Open | Nardi 2018 | 190 | No | Monitoring systems | Europe | Yes | Yes | Yes - clinicaltrials.gov | Yes | Other | No | Non-industry |
| Terlipressin or norepinephrine in hyperdynamic septic shock: a prospective, randomized study | Crit Care Med | Albanese 2005 | 20 | No | Therapeutic interventions | Europe | No | No | No | No | Other | No | Not reported |
| Terlipressin versus norepinephrine as infusion in patients with septic shock: a multicentre, randomised, double-blinded trial | Intensive Care Med | Liu 2018 | 526 | Yes | Therapeutic interventions | Asia/Pacific | No | Yes | Yes - clinicaltrials.gov | Yes | Mortality | No | Non-industry |
| Testing a conceptual model on early opening of the microcirculation in severe sepsis and septic shock <i>A randomised controlled pilot study</i> | Eur J Anaesthesiol | vanderVoort 2015 | 90 | No | Other strategies | Europe | No | No | Yes - clinicaltrials.gov | Yes | Other | No | Non-industry |
| The clinical and paraclinical effectiveness of four-hour infusion vs. half-hour infusion of high-dose ampicillin-sulbactam in treatment of critically ill patients with sepsis or septic shock: an assessor-blinded randomized clinical trial | J Crit Care | Mirjalili 2023 | 136 | No | Therapeutic interventions | Asia/Pacific | No | Yes | Yes - Other | Yes | Other | Yes | Non-industry |
| The clinical effectiveness of sivelestat in treating sepsis patients with both acute respiratory distress syndrome and septic cardiomyopathy. | Journal of Cardiothoracic Surge | Lv 2024 | 70 | Yes | Therapeutic interventions | Asia/Pacific | No | No | Yes - Other | Yes | Other | Yes | Non-industry |
| The contrasting effects of dobutamine and dopamine on gastric mucosal perfusion in septic patients | Am J Respir Crit Care Med | Neviere 1996 | | No | Therapeutic interventions | Europe | No | No | No | No | Other | Yes | Not reported |
| The contrasting effects of dopamine and norepinephrine on systemic and splanchnic oxygen utilization in hyperdynamic sepsis | JAMA | Marik 1994 | 20 | No | Therapeutic interventions | North America | No | No | No | No | Other | Yes | Not reported |
| The effect of a novel extracorporeal cytokine hemoadsorption device on IL-6 elimination in septic patients: A randomized controlled trial | PLoS One | Schadler 2017 | 100 | Yes | Other strategies | Europe | No | Yes | Yes - clinicaltrials.gov | Yes | Other | No | Industry |
| The effect of atrial natriuretic peptide infusion on intestinal injury in septic shock | J Anaesthesiol Clin Pharmacol | Elbaradey 2016 | 40 | No | Therapeutic interventions | Africa | No | No | No | No | Other | Yes | Non-industry |
| The effect of body position changes on stroke volume variation in 66 mechanically ventilated patients with sepsis | J Crit Care | Daihua 2012 | 60 | No | Other strategies | Asia/Pacific | No | No | No | No | Other | Yes | Not reported |
| The effect of continuous infusion of meropenem antibiotic on clinical signs and changes of procalcitonin in patients with acute sepsis related to cancer | Oncology and Radiotherapy | Farokhi 2020 | 60 | No | Therapeutic interventions | Asia/Pacific | No | No | No | No | Other | Yes | Not reported |
| The effect of glutathione and N-acetylcysteine on lipoperoxidative damage in patients with early septic shock | Am J Respir Crit Care Med | Ortolani 2000 | 30 | No | Therapeutic interventions | Europe | No | Yes | No | No | Other | Yes | Not reported |
| The effect of N-acetylcysteine on nuclear factor-kappa B activation, interleukin-6, interleukin-8, and intercellular adhesion molecule-1 expression in patients with sepsis | Crit Care Med | Paterson 2003 | 20 | No | Therapeutic interventions | Europe | No | No | No | No | Other | Yes | Non-industry |
| The effect of Na-selenite treatment on the oxidative stress-antioxidants balance of multiple organ failure | J Crit Care | Woth 2014 | 40 | No | Therapeutic interventions | Europe | No | No | No | No | Other | No | Not reported |
| The effect of polymyxin B hemoperfusion on modulation of human leukocyte antigen DR in severe sepsis patients | Crit Care | Srisawat 2018 | 59 | No | Other strategies | Asia/Pacific | No | No | Yes - clinicaltrials.gov | Yes | Other | Yes | Non-industry |
| The Effect of Xinmailong Infusion on Sepsis-Induced Myocardial Dysfunction: a Pragmatic Randomized Controlled Trial | Shock | He 2021 | 192 | Yes | Therapeutic interventions | Asia/Pacific | No | Yes | Yes - Other | Yes | Other | Yes | Non-industry |
| The Effects of Atorvastatin on Inflammatory Responses and Mortality in Septic Shock: a Single-center, Randomized Controlled Trial | Indian J Crit Care Med | Singh 2017 | 73 | Yes | Therapeutic interventions | Asia/Pacific | No | No | Yes - clinicaltrials.gov | Yes | Mortality | No | Non-industry |
| The Effects of Genistein as Supplement to Oral/ Enteral Nutrition on Inflammatory Cytokines in Septic ICU patients: a Prospective, Single-center, Randomized Controlled Pilot Study | Journal of Clinical Practice and Research | Elay 2023 | 16 | No | Other strategies | Europe | No | No | Yes - clinicaltrials.gov | Yes | Other | No | Not reported |
| The effects of hydroxyethyl starch solution in critically ill patients | Minerva Anestesiol | Palumbo 2006 | 20 | No | Other strategies | Europe | No | No | No | No | Other | Yes | Not reported |
| The effects of hypertonic fluid administration on the gene expression of inflammatory mediators in circulating leucocytes in patients with septic shock: a preliminary study. | Annals of intensive care | vanHaren 2011 | 24 | No | Other strategies | Asia/Pacific | No | No | Yes - Other | Yes | Other | Yes | Non-industry |
| The effects of ibuprofen on the physiology and survival of patients with sepsis. The Ibuprofen in Sepsis Study Group | N Engl J Med | Bernard 1997 | 455 | No | Therapeutic interventions | North America | Yes | Yes | No | Yes | Mortality | No | Non-industry |
| The effects of IgM-enriched immunoglobulin preparations in patients with severe sepsis [ISRCTN28863830] | Crit Care | Tugrul 2002 | 21 | No | Therapeutic interventions | Europe | No | No | Yes - Other | Yes | Other | No | Not reported |
| The effects of intravenous antioxidants in patients with septic shock | Free Radic Biol Med | Galley 1997 | 16 | No | Therapeutic interventions | Europe | No | No | No | No | Other | Yes | Non-industry |
| The effects of L-carnitine supplementation on inflammation, oxidative stress, and clinical outcomes in critically Ill patients with sepsis: a randomized, double-blind, controlled trial | Nutrition Journal | Keshani 2024 | 60 | No | Other strategies | Asia/Pacific | No | No | Yes - Other | Yes | Other | Yes | Non-industry |
| The effects of levosimendan vs dobutamine added to dopamine on liver functions assessed with noninvasive liver function monitoring in patients with septic shock | J Crit Care | Memis 2012 | 30 | No | Therapeutic interventions | Europe | No | No | No | No | Other | Yes | Non-industry |
| The effects of moderate-dose steroid therapy in sepsis: A placebo-controlled, randomized study | J Res Med Sci | Yildiz 2011 | 55 | No | Therapeutic interventions | Europe | No | No | Yes - clinicaltrials.gov | Yes | Mortality | No | Not reported |
| The effects of saffron supplementation on inflammation and hematological parameters in patients with sepsis: a randomized controlled trial. | Nutrition journal | Hassanizadeh 2025 | 90 | No | Other strategies | Asia/Pacific | No | No | Yes - Other | Yes | Other | Yes | Non-industry |
| The efficacy and safety of thymosin alpha1 for sepsis (TESTS): multicentre, double blinded, randomised, placebo controlled, phase 3 trial. | BMJ | Wu 2025 | 1106 | Yes | Therapeutic interventions | Asia/Pacific | No | No | Yes - clinicaltrials.gov | Yes | Mortality | No | Industry |
| The efficacy of modified HuangLian JieDu decoction for early enteral nutrition in patients with sepsis: a randomized controlled study | Medicine (Baltimore) | Wang 2022 | 86 | No | Therapeutic interventions | Asia/Pacific | No | No | Yes - Other | Yes | Mortality | No | Non-industry |
| The efficacy of thymosin alpha 1 for severe sepsis (ETASS): a multicenter, single-blind, randomized and controlled trial | Crit Care | Wu 2013 | 361 | Yes | Therapeutic interventions | Asia/Pacific | No | No | Yes - clinicaltrials.gov | Yes | Mortality | No | Non-industry |
| The immunological benefit of higher dose N-acetyl cysteine following mechanical ventilation in critically ill patients | Daru | Najafi 2014 | 44 | No | Therapeutic interventions | Asia/Pacific | No | Yes | Yes - Other | Yes | Other | No | Non-industry |
| The impact of an omega-3 fatty acid rich lipid emulsion on fatty acid profiles in critically ill septic patients | Prostaglandins Leukot Essent Fatty Acids | Hall 2016 | 41 | No | Other strategies | Europe | No | No | No | No | Other | Yes | Industry |
| The impact of lactate-buffered high-volume hemofiltration on acid-base balance | Intensive Care Med | Cole 2003 | 10 | No | Other strategies | Asia/Pacific | No | No | No | No | Other | No | Industry |
| The influence of alpha-tocopherol on cytokine levels and gastric intramucosal pH in severe sepsis | Balkan Medican Journal | Memis 2008 | 20 | No | Therapeutic interventions | Europe | No | No | No | No | Other | No | Not reported |
| The influence of methylene blue infusion on cytokine levels during severe sepsis | Anaesth pain intensive care | Memis 2002 | 15 | No | Therapeutic interventions | Europe | No | No | No | No | Other | No | Not reported |
| The influence of N-acetyl cysteine infusion oncytokine levels and gastric intramucosal pH during severe sepsis | Crit Care | Emet 2004 | 53 | No | Therapeutic interventions | Europe | No | No | No | No | Other | No | Not reported |
| The premature closure of ROMPA clinical trial: mortality reduction in septic shock by plasma adsorption | BMJ Open | Gimenez-Esparza 2019 | 49 | Yes | Other strategies | Europe | No | Yes | Yes - clinicaltrials.gov | Yes | Mortality | No | Industry |
| The protective effect of ulinastatin in severe sepsis. A mechanistic approach | Farmacia | Zhou 2020 | 16 | No | Therapeutic interventions | Asia/Pacific | No | No | No | No | Mortality | Yes | Not reported |
| The Restrictive IV Fluid Trial in Severe Sepsis and Septic Shock (RIFTS): a Randomized Pilot Study | Crit Care Med | Corl 2019 | 109 | Yes | Other strategies | North America | No | Yes | Yes - clinicaltrials.gov | Yes | Mortality | No | Non-industry |
| Therapeutic effects of curcumin and piperine combination in critically ill patients with sepsis: a randomized double-blind controlled trial. | Trials | Alikiaii 2025 | 66 | No | Therapeutic interventions | Asia/Pacific | No | No | Yes - Other | Yes | Other | Yes | Non-industry |
| Therapeutic Hyperthermia Is Associated With Improved Survival in Afebrile Critically Ill Patients With Sepsis: a Pilot Randomized Trial | Crit Care Med | Drewry 2022 | 56 | Yes | Other strategies | North America | No | No | Yes - clinicaltrials.gov | Yes | Other | No | Non-industry |
| Therapeutic Plasma Exchange Protects Patients with Sepsis-Associated Disseminated Intravascular Coagulation by Improving Endothelial Function | Clin Appl Thromb Hemost | Weng 2021 | 40 | No | Other strategies | Asia/Pacific | No | No | No | No | Mortality | Yes | Non-industry |
| Thiamine for Renal Protection in Septic Shock (TRPSS): a Randomized, Placebo-controlled, Clinical Trial | Am J Respir Crit Care Med | Moskowitz 2023 | 88 | Yes | Therapeutic interventions | North America | No | Yes | Yes - clinicaltrials.gov | Yes | Other | No | Non-industry |
| Time course of organ failure in patients with septic shock treated with hydrocortisone: results of the Corticus study | Intensive Care Med | Moreno 2011 | 499 | No | Therapeutic interventions | Europe | Yes | Yes | Yes - Other | Yes | Mortality | No | Non-industry |
| Timing of Renal-Replacement Therapy in Patients with Acute Kidney Injury and Sepsis | N Engl J Med | Barbar 2018 | 488 | Yes | Other strategies | Europe | No | Yes | Yes - clinicaltrials.gov | Yes | Mortality | No | Non-industry |
| Traditional Chinese medicine bundle therapy for septic acute gastrointestinal injury: a multicenter randomized controlled trial | Complement Ther Med | Xing 2019 | 302 | No | Therapeutic interventions | Asia/Pacific | No | Yes | Yes - Other | Yes | Mortality | Yes | Non-industry |
| Treatment of patients with severe sepsis using ulinastatin and thymosin alpha1: a prospective, randomized, controlled pilot study | Chin Med J | Chen 2009 | 114 | No | Therapeutic interventions | Asia/Pacific | No | Yes | No | No | Other | Yes | Non-industry |
| Treatment of septic shock with the tumor necrosis factor receptor: fc fusion protein. The Soluble TNF Receptor Sepsis Study Group | N Engl J Med | Fisher 1996 | 141 | Yes | Therapeutic interventions | North America | No | Yes | No | No | Mortality | No | Industry |
| Triiodothyronine hormone supplementation therapy in septic shock patients with euthyroid sick syndrome: two pilot, placebo-controlled, randomized trials. | Anaesth Crit Care Pain Med | Kovacevic 2004 | 95 | Yes | Therapeutic interventions | Europe | No | No | Yes - clinicaltrials.gov | Yes | Mortality | Yes | Not reported |
| Ubiquinol (reduced coenzyme Q10) in patients with severe sepsis or septic shock-a randomized, double-blind, placebo-controlled pilot trial | Crit Care | Donnino 2015 | 41 | Yes | Therapeutic interventions | North America | No | No | Yes - clinicaltrials.gov | Yes | Other | Yes | Industry |
| Ultrasound-Guided Fluid Volume Management in Patients With Septic Shock: A Randomized Controlled Trial. | Journal of trauma nursing : the official journal of the Society of Trauma Nurses | Li 2025 | 113 | No | Care bundles | Asia/Pacific | No | No | No | No | Other | Yes | Not reported |
| Use of heptaminol hydrochloride for catecholamine weaning in septic shock | American Journal of Therapeutics | Bahloul 2012 | 49 | Yes | Therapeutic interventions | Asia/Pacific | No | No | No | Yes | Other | Yes | Not reported |
| Use of military anti-shock trousers in resuscitation of acute circulatory failure patients in intensive care unit | Royal Thai Army Medical Journal | Boontoterm 2022 | 60 | Yes | Other strategies | Asia/Pacific | No | No | No | No | Other | Yes | Non-industry |
| Use of procalcitonin to shorten antibiotic treatment duration in septic patients: a randomized trial | Am J Respir Crit Care Med | Nobre 2008 | 79 | Yes | Monitoring systems | Europe | No | No | Yes - clinicaltrials.gov | Yes | Other | Yes | Non-industry |
| Use of stepwise lactate kinetics-oriented hemodynamic therapy could improve the clinical outcomes of patients with sepsis-associated hyperlactatemia | Crit Care | Zhou 2017 | 360 | No | Other strategies | Asia/Pacific | No | No | Yes - clinicaltrials.gov | Yes | Mortality | Yes | Non-industry |
| Using Body Composition Analysis for Improved Nutritional Intervention in Septic Patients: a Prospective Interventional Study | Nutrients | Hung 2023 | 132 | Yes | Other strategies | Asia/Pacific | No | No | Yes - clinicaltrials.gov | Yes | Other | No | Non-industry |
| Utilization of NIRS Monitor to Compare the Regional Cerebral Oxygen Saturation Between Dexmedetomidine and Propofol Sedation in Mechanically Ventilated Critically ill Patients with Sepsis- A Prospective Randomized Control Trial. | J Intensive Care Med | Patidar 2025 | 54 | Yes | Monitoring systems | Asia/Pacific | No | No | Yes - Other | Yes | Other | Yes | Not reported |
| Vasopressin in Septic Shock; Assessment of Sepsis Biomarkers: a Randomized, Controlled Trial | Indian J Crit Care Med | Barzegar 2017 | 42 | No | Therapeutic interventions | Asia/Pacific | No | Yes | Yes - Other | Yes | Other | No | Non-industry |
| Vasopressin Versus Norepinephrine for the Management of Septic Shock in Cancer Patients: the VANCS II Randomized Clinical Trial | Crit Care Med | Hajjar 2019 | 250 | Yes | Therapeutic interventions | Latin America | No | No | Yes - clinicaltrials.gov | Yes | Mortality | No | Non-industry |
| Very Early Use of Esmolol in Hyperkinetic Septic Shock Patients with Persistent Tachycardia: A Randomized Controlled Pilot Study | Shock | Lu 2025 | 12 | No | Therapeutic interventions | Asia/Pacific | No | No | Yes - Other | Yes | Other | Yes | Non-industry |
| Very high volume hemofiltration with the Cascade system in septic shock patients | Intensive Care Med | Quenot 2015 | 60 | Yes | Other strategies | Europe | No | Yes | Yes - clinicaltrials.gov | Yes | Other | No | Industry |
| Vitamin A treatment for severe sepsis in humans; a prospective randomized double blind placebo-controlled clinical trial | Clin Nutr | Cherukuri 2019 | 63 | No | Therapeutic interventions | North America | No | No | Yes - clinicaltrials.gov | Yes | Mortality | No | Industry |
| Vitamin c therapy or routine care in septic shock (ViCTOR) trial: effect of intravenous vitamin C, thiamine, and hydrocortisone administration on inpatient mortality among patients with septic shock | Indian J Crit Care Med | Mohamed 2020 | 88 | Yes | Therapeutic interventions | Asia/Pacific | No | No | Yes - Other | Yes | Mortality | No | Non-industry |

## **Table S5B: Risk of bias of the randomized controlled trials (N = 591) included in the systematic review**

| **Study title** | **Study ID** | **Allocation concealment** | **Blinding of participants and personnel** | **Blinding of outcome assessment** | **Incomplete outcome data** | **Selective reporting** | **Other sources of bias** |
| --- | --- | --- | --- | --- | --- | --- | --- |
| (1,3)-beta-D-Glucan-guided antifungal therapy in adults with sepsis: the CandiSep randomized clinical trial. | Bloos 2022 | Low | High | Low | Low | Low | Unsure |
| (1,3)-beta-D-Glucan-based empirical antifungal interruption in suspected invasive candidiasis: a randomized trial. | DePascale 2020 | Low | High | High | Low | Low | Unsure |
| A dose-finding study of methylene blue to inhibit nitric oxide actions in the hemodynamics of human septic shock | Juffermans 2010 | Low | Low | Low | Low | Unsure | High |
| A modified goal-directed protocol improves clinical outcomes in intensive care unit patients with septic shock: a randomized controlled trial | Lin 2006 | Unsure | High | High | Low | Unsure | High |
| A multicenter randomized trial of atorvastatin therapy in intensive care patients with severe sepsis | Kruger 2013 | Low | Low | Low | Low | Low | Unsure |
| A multicenter randomized trial of continuous versus intermittent beta-lactam infusion in severe sepsis | Dulhunty 2015 | Low | Low | Low | Low | Low | Low |
| A multicenter, open-label, prospective, randomized, dose-ranging pharmacokinetic study of the anti-TNF-alpha antibody afelimomab in patients with sepsis syndrome | Gallagher 2001 | Unsure | High | High | Low | Unsure | High |
| A new immunomodulatory therapy for severe sepsis: ulinastatin Plus Thymosin {alpha} 1 | Li 2009 | Unsure | Low | Unsure | Low | Unsure | High |
| A phase 2 randomized, placebo-controlled trial of inulin for the prevention of gut pathogen colonization and infection among patients admitted to the intensive care unit for sepsis. | Park 2025 | Low | Low | Low | Low | Low | Unsure |
| A phase I trial evaluating the safety, tolerability, pharmacokinetics and pharmacodynamics of intravenously administered low-anticoagulant heparin (M6229) in critically ill sepsis patients | vanMourik 2025 | High | High | Low | Low | Low | High |
| A phase I trial of low-dose inhaled carbon monoxide in sepsis-induced ARDS | Fredenburgh 2018 | Low | Unsure | Low | Low | Low | High |
| A phase II randomized, controlled trial of continuous hemofiltration in sepsis | Cole 2002 | Unsure | Unsure | Low | Low | High | High |
| A phase II, single-center, double-blind, randomized placebo-controlled trial to explore the efficacy and safety of intravenous melatonin in surgical patients with severe sepsis admitted to the intensive care unit | Mansilla-Rosello 2023 | Low | Low | Low | Low | Low | High |
| A Pilot Double-Blind Placebo-Controlled Randomized Clinical Trial to Investigate the Effects of Early Enteral Nutrients in Sepsis | Shah 2021 | Low | Low | Low | Low | Low | High |
| A Pilot Feasibility Randomized Controlled Trial of Intravenous Vitamin C in Adults with Sepsis in the Intensive Care Unit: the Lessening Organ Dysfunction with Vitamin C-India (LOVIT-India) Trial | Vijayaraghavan 2023 | Low | Low | Low | Low | Low | High |
| A pilot randomised controlled trial in intensive care patients comparing 7 days' treatment with empirical antibiotics with 2 days' treatment for hospital-acquired infection of unknown origin. | Scawn 2012 | Low | High | Low | Low | Low | Low |
| A pilot randomized controlled trial of comparison between extended daily hemodialysis and continuous veno-venous hemodialysis in patients of acute kidney injury with septic shock | Mishra 2017 | Unsure | High | High | Low | Low | High |
| A pilot randomized study comparing high and low volume hemofiltration on vasopressor use in septic shock | Boussekey 2008 | High | High | Unsure | Low | High | High |
| A pilot study of coupled plasma filtration with adsorption in septic shock | Ronco 2002 | Unsure | High | High | Low | Unsure | High |
| A pilot study of high-adsorption hemofiltration in human septic shock | Haase 2007 | Low | High | High | High | Unsure | High |
| A pilot-controlled study of a polymyxim B-immobilized hemoperfusion cartridge in patients with severe sepsis secondary to intra-abdominal infection | Vincent 2005 | Unsure | High | High | Low | Low | High |
| A placebo-controlled, double-blind, dose-escalation study to assess the safety, tolerability and pharmacokinetics/pharmacodynamics of single and multiple intravenous infusions of AZD9773 in patients with severe sepsis and septic shock | Morris 2012 | Low | Low | Low | Low | Low | High |
| A prospective open label randomized noninferiority trial to compare the efficacy and safety of monotherapy with noradrenaline and terlipressin in patients of cirrhosis with septic shock admitted to the intensive care unit (nct01836224) | Choudhury 2014 | Low | High | Unsure | Low | Low | High |
| A prospective, randomised clinical study comparing triple therapy regimen to hydrocortisone monotherapy in reducing mortality in septic shock patients | Hussein 2021 | Unsure | High | High | Low | Low | Unsure |
| A randomised-controlled trial (TARGET-C) of high vs. low target mean arterial pressure in patients with cirrhosis and septic shock | Maiwall 2023 | Low | High | Low | Low | Low | High |
| A randomized and controlled trial of the effect of treatment aimed at maximizing oxygen delivery in patients with severe sepsis or septic shock | Alia 1999 | Low | High | Low | Low | Unsure | Unsure |
| A randomized controlled trial investigating the effects of parenteral fish oil on survival outcomes in critically ill patients with sepsis: a pilot study | Hall 2015 | Unsure | High | High | Low | Unsure | High |
| A randomized phase II trial of granulocyte-macrophage colony-stimulating factor therapy in severe sepsis with respiratory dysfunction | Presneill 2002 | Unsure | Low | Low | Low | Unsure | High |
| A randomized pilot study of parenteral glutamine supplementation in severe sepsis | Poulose 2017 | Low | Low | Low | Low | Low | High |
| A Randomized Trial of Mycobacterium w in Severe Presumed Gram-Negative Sepsis | Sehgal 2021 | Low | Low | Low | Low | Low | High |
| A randomized trial of Mycobacterium w in severe sepsis | Sehgal 2015 | Low | Low | Low | Low | Low | High |
| A randomized trial to compare procalcitonin and C-reactive protein in assessing severity of sepsis and in guiding antibacterial therapy in Egyptian critically ill patients | Ali 2021 | Low | High | Unsure | Low | Low | Low |
| A randomized-controlled trial of arginine infusion in severe sepsis on microcirculation and metabolism | Luiking 2020 | Low | Low | Low | Low | Low | High |
| A randomized, double-blind, placebo-controlled trial of TAK-242 for the treatment of severe sepsis | Rice 2010 | Low | Low | Low | Low | Unsure | High |
| A randomized, double-blind, placebo-controlled, Phase 2b study to evaluate the safety and efficacy of recombinant human soluble thrombomodulin, ART-123, in patients with sepsis and suspected disseminated intravascular coagulation | Vincent 2013 | Low | Low | Low | Low | Low | High |
| A second large controlled clinical study of E5, a monoclonal antibody to endotoxin: results of a prospective, multicenter, randomized, controlled trial. The E5 Sepsis Study Group | Bone 1995 | Unsure | Low | Low | Unsure | High | High |
| ACETATE RINGER'S SOLUTION VERSUS NORMAL SALINE SOLUTION IN SEPSIS: A RANDOMIZED, CONTROLLED TRIAL. | Zhang 2024 | High | High | Low | Low | Low | High |
| Acetylsalicylic Acid Treatment in Patients With Sepsis and Septic Shock: A Phase 2, Placebo-Controlled, Randomized Clinical Trial. | Almeida 2025 | Low | Low | Low | Low | Low | Unsure |
| Acute bag-valve breathing maneuvers plus manual chest compression is safe during stable septic shock: a randomized clinical trial | Blattner 2017 | Low | High | Low | Low | Unsure | High |
| Acute haemodynamic effects of a hypertonic saline/dextran solution in stable patients with severe sepsis | Oliveira 2002 | Unsure | Low | Low | Low | Unsure | High |
| Addition of terlipressin to norepinephrine in septic shock and effect of renal perfusion: a pilot study | Wang 2022 | Low | High | Low | Low | Low | High |
| Adjunctive Glucocorticoid Therapy in Patients with Septic Shock | Venkatesh 2018 | Low | Low | Low | Low | Low | Low |
| Adjunctive granisetron therapy in patients with sepsis or septic shock (GRANTISS): a single-center, single-blinded, randomized, controlled clinical trial | Guan 2022 | Low | High | Low | Low | Low | High |
| Adjunctive homeopathic treatment in patients with severe sepsis: a randomized, double-blind, placebo-controlled trial in an intensive care unit | Frass 2005 | Low | Low | Low | Unsure | Unsure | High |
| Adjunctive Sedation with Dexmedetomidine for the Prevention of Severe Inflammation and Septic Encephalopathy: A Pilot Randomized Controlled Study. | Iten 2025 | Low | High | Low | High | Low | High |
| Adjunctive sepsis therapy with aminophylline (STAP): a randomized controlled trial | Zhang 2022 | Low | High | Low | Low | Low | High |
| Administration of amphotericin B in lipid emulsion decreases nephrotoxicity: results of a prospective, randomized, controlled study in critically ill patients. | Sorkine 1996 | Unsure | High | Unsure | Unsure | Unsure | High |
| Administration of higher doses of amikacin in early stages of sepsis in critically ill patients | Najmeddin 2014 | High | High | High | High | Unsure | High |
| Administration of low-dose dopamine to nonoliguric patients with sepsis syndrome does not raise intramucosal gastric pH nor improve creatinine clearance | Olson 1996 | Unsure | Low | Low | Low | Unsure | High |
| Administration of the nitric oxide synthase inhibitor NG-methyl-L-arginine hydrochloride (546C88) by intravenous infusion for up to 72 hours can promote the resolution of shock in patients with severe sepsis: results of a randomized, double-blind, placebo | Bakker 2004 | Low | Low | Low | Low | Unsure | Unsure |
| Aerosolized prostacyclin and inhaled nitric oxide in septic shock--different effects on splanchnic oxygenation? | Eichelbronner 1996 | Unsure | High | High | Low | Unsure | High |
| Albumin replacement in patients with severe sepsis or septic shock | Caironi 2014 | Low | High | Low | Low | Low | Low |
| Alkaline phosphatase for treatment of sepsis-induced acute kidney injury: a prospective randomized double-blind placebo-controlled trial | Pickkers 2012 | Low | Low | Low | Low | Low | High |
| Alkaline phosphatase treatment improves renal function in severe sepsis or septic shock patients | Heemskerk 2009 | Low | Low | Low | Low | Unsure | High |
| Alteco endotoxin hemoadsorption in Gram-negative septic shock patients | Shum 2014 | Low | High | High | Low | Low | High |
| An Electronic Tool for the Evaluation and Treatment of Sepsis in the ICU: a Randomized Controlled Trial | Semler 2015 | Unsure | High | Low | Low | Low | Low |
| An open-label, randomized controlled trial to assess a ketogenic diet in critically ill patients with sepsis. | Rahmel 2024 | Unsure | High | Low | Low | Low | High |
| An open-label, randomized, phase 3 study of the efficacy and safety of antithrombin gamma in patients with sepsis-induced disseminated intravascular coagulation syndrome | AntithrombinGammaStudyGrp 2018 | Unsure | High | Unsure | High | Low | High |
| Analysis on the effects of dexmedetomidine on ventilator free days and mortality in sepsis patients receiving mechanical ventilaiton | Kushare 2024 | Unsure | High | High | Low | Unsure | High |
| Antibiotic stewardship program in Intensive Care Unit: First report from Iran. | Vahidi 2018 | Unsure | High | High | Low | Low | High |
| Antithrombin III in patients with severe sepsis: a pharmacokinetic study | Ilias 2000 | Unsure | High | High | Low | Unsure | High |
| Antithrombin III in patients with severe sepsis. A randomized, placeho-controlled, double-blind multicenter trial plus a meta-analysis on all randomized, placebo-controlled, double-blind trials with antithrombin III in severe sepsis | Eisele 1998 | Unsure | Low | Low | Low | Unsure | High |
| Application of a combination of lactated Ringer's solution and ulinastatin for early resuscitation in sepsis | Min 2023 | Unsure | High | High | Low | Unsure | High |
| Application of ultrasound-guided external jugular vein puncture in intensive care unit (ICU) patients with severe sepsis: a randomised trial | Luo 2021 | Low | Low | Low | Low | Low | Low |
| Assessment of hemodynamic efficacy and safety of 6% hydroxyethylstarch 130/0.4 vs. 0.9% NaCl fluid replacement in patients with severe sepsis: the CRYSTMAS study | Guidet 2012 | Low | Low | Low | Low | Low | High |
| Assessment of the effect of unfractionated heparin administered either by intravenous infusion vs. subcutaneous injection on heparin-binding protein, and plasminogen activator inhibitor-1 in critically ill septic patients: a randomized controlled trial. | Kassem 2024 | Unsure | High | Unsure | Low | Low | High |
| Assessment of the safety and efficacy of the monoclonal anti-tumor necrosis factor antibody-fragment, MAK 195F, in patients with sepsis and septic shock: a multicenter, randomized, placebo-controlled, dose-ranging study | Reinhart 1996 | Unsure | Low | Low | Low | Unsure | High |
| Assessment of the safety of recombinant tissue factor pathway inhibitor in patients with severe sepsis: a multicenter, randomized, placebo-controlled, single-blind, dose escalation study | Abraham 2001 | Unsure | High | Low | Low | Unsure | Unsure |
| Beneficial effects of short-term vasopressin infusion during severe septic shock | Patel 2002 | Unsure | Low | Low | Low | Unsure | High |
| Beta-Lactam Infusion in Severe Sepsis (BLISS): a prospective, two-centre, open-labelled randomised controlled trial of continuous versus intermittent beta-lactam infusion in critically ill patients with severe sepsis | Abdul-Aziz 2016 | Low | High | Low | Low | Low | Unsure |
| Biomarker-Guided Antibiotic Duration for Hospitalized Patients With Suspected Sepsis: The ADAPT-Sepsis Randomized Clinical Trial. | Dark 2025 | Low | Low | Low | Low | Low | Low |
| C1-esterase inhibitor infusion increases survival rates for patients with sepsis* | Igonin 2012 | Unsure | High | High | Low | Unsure | High |
| C1-inhibitor in patients with severe sepsis and septic shock: beneficial effect on renal dysfunction | Caliezi 2002 | Low | Low | Low | Low | Unsure | High |
| Calcitriol in Sepsis-A Single-Centre Randomised Control Trial. | Thampi 2024 | Low | Low | Low | Low | Unsure | Unsure |
| Can the global end-diastolic volume index guide fluid management in septic patients? A multicenter randomized controlled trial | Morisawa 2020 | Unsure | High | High | Low | Low | High |
| Cardiovascular effects of the nitric oxide synthase inhibitor NG-methyl-L-arginine hydrochloride (546C88) in patients with septic shock: results of a randomized, double-blind, placebo-controlled multicenter study (study no. 144-002) | Watson 2004 | Unsure | Low | Low | Low | Unsure | High |
| CD14 receptor occupancy in severe sepsis: results of a phase I clinical trial with a recombinant chimeric CD14 monoclonal antibody (IC14) | Reinhart 2004 | Unsure | Low | Low | Low | Unsure | High |
| CDP571, a humanized antibody to human tumor necrosis factor-alpha: Safety, pharmacokinetics, immune response, and influence of the antibody on cytokine concentrations in patients with septic shock | Dhainaut 1995 | Low | Low | Low | Low | Unsure | Unsure |
| Cerebral autoregulation-directed optimal blood pressure management reduced the risk of delirium in patients with septic shock | Peng 2024 | Unsure | High | High | Low | Low | High |
| Ciprofol versus propofol for long-term sedation in mechanically ventilated patients with sepsis: a randomized controlled trial. | Zhao 2025 | Unsure | High | Low | Low | Low | High |
| Circulating protein carbonyls are specifically elevated in critically ill patients with pneumonia relative to other sources of sepsis | Spencer 2022 | Unsure | Low | Low | Unsure | Unsure | High |
| Clearance of vancomycin during high-volume haemofiltration: Impact of pre-dilution | Uchino 2002 | Unsure | High | Low | Low | Low | High |
| Clinical and biochemical endpoints and predictors of response to plasma exchange in septic shock: results from a randomized controlled trial | Stahl 2022 | Unsure | High | Low | Low | Low | High |
| Clinical and economic impact of procalcitonin to shorten antimicrobial therapy in septic patients with proven bacterial infection in an intensive care setting | Deliberato 2013 | Unsure | High | High | High | Unsure | High |
| Clinical and metabolic effects of two lipid emulsions on the parenteral nutrition of septic patients | Garnacho-Montero 2002 | Unsure | High | High | High | Unsure | High |
| Clinical Assessment of Continuous Hemodialysis with the Medium Cutoff EMiC√Ç¬Æ2 Membrane in Patients with Septic Shock | Ferrari 2022 | Unsure | High | Low | Low | Low | Low |
| Clinical effect of combined ulinastatin and continuous renal replacement therapy on management of severe sepsis with acute kidney injury | Fang 2017 | Unsure | High | High | High | Unsure | High |
| Clinical efficacy of continuous infusion of piperacillin compared with intermittent dosing in septic critically ill patients | Rafati 2006 | Unsure | High | High | Low | Unsure | High |
| Clinical efficacy of ulinastatin in the treatment of unliquefied pyogenic liver abscess complicated by septic shock: a randomized controlled trial | Guo 2023 | Unsure | High | High | Low | Unsure | High |
| Clinical outcome study of critically-ill septic patients given taurine supplemented enteral nutrition | Elmokadem 2015 | Unsure | High | High | Low | Low | High |
| Clinical outcomes of empirical high-dose meropenem in critically ill patients with sepsis and septic shock: a randomized controlled trial | Lertwattanachai 2020 | Low | High | Low | Low | Low | High |
| Clinical Trial Assessment of Intermittent and Continuous Infusion Dose of N-Acetylcysteine on Redox Status of the Body in Patients with Sepsis Admitted to the ICU | PeivandiYazdi 2020 | Unsure | Low | Low | Low | Low | High |
| Co-administration of iloprost and eptifibatide in septic shock (CO-ILEPSS)-a randomised, controlled, double-blind investigator-initiated trial investigating safety and efficacy | Berthelsen 2019 | Low | Low | Unsure | High | Unsure | High |
| Combination therapy of vitamin c and thiamine on matrix metalloproteinas-es-9 (Mmp-9) for septic in icu | Lubis 2021 | Unsure | Low | Unsure | High | Unsure | High |
| Combination therapy with milrinone and esmolol for heart protection in patients with severe sepsis: a prospective, randomized trial | Wang 2015 | Unsure | High | Unsure | Low | Unsure | High |
| Combined Treatment With Hydrocortisone, Vitamin C, and Thiamine for Sepsis and Septic Shock: a Randomized Controlled Trial | Chang 2020 | Unsure | High | Low | Unsure | Low | High |
| Comparable Effect of Two-Step Versus Extended Infusions on the Pharmacokinetics of Imipenem in Patients with Sepsis and Septic Shock | Huang 2020 | Unsure | High | Low | Low | Low | High |
| Comparative efficacy and prognostic impact of continuous versus intermittent hydrocortisone administration in septic shock patients. | Jin 2025 | Low | Low | Low | Low | Unsure | High |
| Comparative evaluation of central venous pressure and sonographic inferior vena cava variability in assessing fluid responsiveness in septic shock | Garg 2016 | Unsure | High | High | Low | Unsure | High |
| Comparative study between high and low dose methylene blue infusion in septic cancer patients: a randomized, blinded, controlled study. | Shaker 2025 | Low | High | Low | Low | Low | High |
| Comparison Between Continuous and Intermittent Administration of Hydrocortisone During Septic Shock: a Randomized Controlled Clinical Trial | Tilouche 2019 | Unsure | High | High | Low | Low | High |
| Comparison Between Norepinephrine Alone Versus Norepinephrine/Vasopressin Combination for Resuscitation in Septic Shock A Randomized Clinical Trial | Hussien 2021 | Low | Low | Low | Low | Low | Unsure |
| Comparison of 6% hydroxyethyl starch 130/0.4 and saline solution for resuscitation of the microcirculation during the early goal-directed therapy of septic patients | Dubin 2010 | Unsure | High | Low | High | Unsure | High |
| Comparison of dexmedetomidine and propofol in mechanically ventilated patients with sepsis: A pilot study | MarkSigler 2018 | Low | High | High | High | Low | High |
| Comparison of intermittent and bolus enteral feeding methods on enteral feeding intolerance of patients with sepsis: a triple-blind controlled trial in intensive care units | Nasiri 2017 | Low | Low | Low | Low | Low | High |
| Comparison of norepinephrine and dobutamine to epinephrine for hemodynamics, lactate metabolism, and gastric tonometric variables in septic shock: a prospective, randomized study | Levy 1997 | Unsure | High | High | Low | Unsure | High |
| Comparison of norepinephrine and dopamine in the management of septic shock using impedance cardiography | Mathur 2007 | Unsure | High | Unsure | Low | Unsure | High |
| Comparison of Norepinephrine and Terlipressin vs Norepinephrine Alone for Management of Septic Shock: a Randomized Control Study | Sahoo 2022 | Low | High | Low | Low | Low | High |
| Comparison of phenylephrine and norepinephrine in the management of dopamine-resistant septic shock | Jain 2010 | Unsure | High | Low | Low | Unsure | High |
| Comparison of systemic and regional effects of dobutamine and dopexamine in norepinephrine-treated septic shock | Levy 1999 | Unsure | High | High | Low | Unsure | High |
| Comparison of systemic and renal effects of dopexamine and dopamine in norepinephrine-treated septic shock | Schmoelz 2006 | Low | Low | Low | Low | Low | Unsure |
| Comparison of the cytokine adsorption ability in continuous renal replacement therapy using polyethyleneimine-coated polyacrylonitrile (AN69ST) or polymethylmethacrylate (PMMA) hemofilters: a pilot single-center open-label randomized control trial. | Nakamura 2023 | Unsure | High | High | Low | Low | High |
| Comparison of the eff ects of intravenous, enteral and enteral + intravenous supply of glutamine on malnutrition in sepsis | Koksal 2011 | Unsure | High | Low | Low | Unsure | High |
| Comparison of the effects of subcutaneous versus continuous infusion of heparin on key inflammatory parameters following sepsis | Nouri 2016 | Unsure | High | Unsure | Low | Low | High |
| Comparison of the effects of vitamin C and thiamine on refractory hypotension in patients with sepsis: A randomized controlled trial | Nandhini 2022 | Unsure | Low | Low | Low | Low | High |
| Comparison of vasopressin and phenylephrine in treatment of dopamine resistant septic shock - A randomised control trial | Patro 2021 | Low | High | Low | Low | Unsure | High |
| Confirmatory interleukin-1 receptor antagonist trial in severe sepsis: a phase III, randomized, double-blind, placebo-controlled, multicenter trial. The Interleukin-1 Receptor Antagonist Sepsis Investigator Group | Opal 1997 | Low | Low | Low | Low | Low | Low |
| Confirmatory platelet-activating factor receptor antagonist trial in patients with severe Gram-negative bacterial sepsis: A phase III, randomized, double-blind, placebo-controlled, multicenter trial | Dhainaut 1998 | Low | Low | Low | Low | Unsure | Unsure |
| Conservative Fluid Management After Sepsis Resuscitation: a Pilot Randomized Trial | Semler 2020 | Low | High | Low | Low | Low | High |
| Continuous infusion of beta-lactam antibiotics in severe sepsis: a multicenter double-blind, randomized controlled trial | Dulhunty 2013 | Low | Low | Low | Low | Low | High |
| Continuous renal replacement therapy in sepsis-associated acute kidney injury: effects on inflammatory mediators and coagulation function | Wu 2021 | Unsure | High | Unsure | Low | Unsure | High |
| Continuous terlipressin versus vasopressin infusion in septic shock (TERLIVAP): a randomized, controlled pilot study | Morelli 2009 | Unsure | High | High | Low | Low | High |
| Continuous veno-venous hemofiltration for septic shock | Hui 2017 | Unsure | High | Unsure | Low | Unsure | High |
| Continuous versus intermittent bolus dosing of beta-lactam antibiotics in a South African multi-disciplinary intensive care unit: A randomized controlled trial. | Khan 2025 | Low | High | Low | Low | Low | High |
| Continuous vs Intermittent beta-Lactam Antibiotic Infusions in Critically Ill Patients With Sepsis: The BLING III Randomized Clinical Trial. | Dulhunty 2024 | Low | High | Low | Low | Low | Low |
| Continuous vs Intermittent Meropenem Administration in Critically Ill Patients With Sepsis: the MERCY Randomized Clinical Trial | Monti 2023 | Low | Low | Low | Low | Low | Low |
| Corticosteroid treatment and intensive insulin therapy for septic shock in adults: a randomized controlled trial | Annane 2010 | Low | High | Low | Low | Low | Unsure |
| Cost-Effectiveness of de novo Simvastatin as Adjunctive Therapy in Patients Critically Ill with Sepsis | MostafaEladawy 2022 | Unsure | High | High | High | Unsure | High |
| Coupled plasma filtration adsorption (CPFA) plus Continuous Veno-Venous Haemofiltration (CVVH) versus CVVH alone as an adjunctive therapy in the treatment of sepsis | Hassan 2013 | Unsure | High | High | High | Unsure | High |
| Critical Care Ultrasound Goal-directed Versus Early Goal-directed Therapy in Septic Shock: a Randomized Controlled Study | Zhang 2021 | Unsure | High | Low | Low | Unsure | High |
| Critically ill septic patients have elevated oxidative stress biomarkers: lack of attenuation by parenteral vitamin C | Vlasiuk 2022 | Unsure | Low | Low | Low | Low | High |
| De-escalation versus continuation of empirical antimicrobial treatment in severe sepsis: a multicenter non-blinded randomized noninferiority trial | Leone 2014 | Low | High | Low | Low | Low | Unsure |
| Determination of end point of fluid resuscitation using simplified lung ultrasound protocol in patients with septic shock | Ismail 2019 | Unsure | High | High | Low | Unsure | High |
| Dexmedetomidine for Reducing Mortality in Patients With Septic Shock: A Randomized Controlled Trial (DecatSepsis). | EzzAl-Regal 2024 | Low | High | Low | Low | Low | High |
| Dexmedetomidine or Propofol for Sedation in Mechanically Ventilated Adults with Sepsis | Hughes 2021 | Low | Low | Low | Low | Low | Low |
| Dexmedetomidine protects against acute kidney injury in patients with septic shock | Liu 2020 | Unsure | High | High | Low | Unsure | High |
| Dexmedetomidine to Reduce Vasopressor Resistance in Refractory Septic Shock: alpha2 Agonist Dexmedetomidine for REfractory Septic Shock (ADRESS): A Double-Blind Randomized Controlled Pilot Trial. | Dargent 2025 | Low | Low | Low | Unsure | Low | High |
| Dexmedetomidine versus propofol or midazolam in patients with abdominal sepsis regarding inflammatory response and capillary leak | Moeen 2022 | Low | High | Low | Low | Low | High |
| Dextrose 5% versus normal saline as maintenance fluid therapy in patients with septic shock (DEMANDS): a randomized controlled trial | Fayed 2024 | Low | High | Low | Low | Low | Unsure |
| Dialysis complications in acute kidney injury patients treated with prolonged intermittent renal replacement therapy sessions lasting 10 versus 6 hours: results of a randomized clinical trial | Albino 2015 | Low | High | Low | Low | Low | Unsure |
| Distribution of normal saline and 5% albumin infusions in septic patients | Ernest 1999 | Unsure | High | Low | Low | Low | High |
| Dobutamine and gastric-to-arterial carbon dioxide gap in severe sepsis without shock | Lebuffe 2002 | Low | High | High | Low | Unsure | High |
| Does Maintaining a Targeted Abdominal Perfusion Pressure Reduce Renal Damage in Patients with Septic Shock?: a Randomized, Controlled, and Open-label Study | Ozkarakas 2023 | Unsure | High | Low | Low | Low | Unsure |
| Does N-acetyl-L-cysteine influence cytokine response during early human septic shock? | Spapen 1998 | Low | Low | Low | Low | Low | High |
| Does nano-curcumin supplementation improve hematological indices in critically ill patients with sepsis? A randomized controlled clinical trial | Naeini 2022 | Unsure | Low | Low | High | Unsure | High |
| Dopexamine and norepinephrine versus epinephrine on gastric perfusion in patients with septic shock: a randomized study [NCT00134212]. | Seguin 2006 | Low | High | Low | Low | Low | High |
| Drotrecogin alfa (activated) for adults with severe sepsis and a low risk of death | Abraham 2005 | Unsure | Low | Low | Low | Low | Unsure |
| Drotrecogin Alfa (Activated) in Adults with Septic Shock | Ranieri 2012 | Low | Low | Low | Low | Low | Low |
| Dyslipidemia: a prospective controlled randomized trial of intensive glycemic control in sepsis | Cappi 2012 | Unsure | High | Unsure | Low | Unsure | High |
| E5 murine monoclonal antiendotoxin antibody in gram-negative sepsis: A randomized controlled trial | Angus 2000 | Low | Low | Low | Low | Low | Unsure |
| Early adjunctive methylene blue in patients with septic shock: a randomized controlled trial | Ibarra-Estrada 2023 | Low | Low | Low | Low | Low | Unsure |
| Early administration of hydrocortisone, vitamin C, and thiamine in adult patients with septic shock: a randomized controlled clinical trial | Lyu 2022 | Low | Low | Low | Low | Low | High |
| Early dexamethasone treatment for septic shock patients: a prospective randomized clinical trial | Cicarelli 2007 | Unsure | Low | Low | Low | High | High |
| Early enteral supplementation with key pharmaconutrients improves Sequential Organ Failure Assessment score in critically ill patients with sepsis: outcome of a randomized, controlled, double-blind trial | Beale 2008 | Unsure | Low | Unsure | High | Unsure | High |
| Early high-dose continuous veno-venous hemofiltration alleviates the alterations of CD4+ T lymphocyte subsets in septic patients combined with acute kidney injury | Jin 2022 | Unsure | High | High | Low | Unsure | High |
| Early Initiation of Low-Dose Hydrocortisone Therapy for Septic Shock in Geriatric Patients: a Randomized Control Trial | Agarwal 2022 | Unsure | High | Low | Low | Unsure | Unsure |
| Early initiation of low-dose hydrocortisone treatment for septic shock in adults: a randomized clinical trial | Lv 2017 | Unsure | Low | Low | Low | Low | High |
| Early Lactate-Guided Resuscitation of Elderly Septic Patients | Chen 2022 | Unsure | High | Unsure | Low | Low | High |
| Early low-dose glucocorticoid therapy effectively suppresses serum pro-inflammatory factors such as IL-6 and inhibits apoptosis of CD4+ cells in septic shock patients | Jiang 2019 | Unsure | High | High | Low | Unsure | High |
| Early peripheral perfusion targeted fluid therapy leads to less fluid administration in patients with septic shock: a prospective randomized controlled trial | VanGenderen 2014 | Unsure | High | Low | Low | Low | High |
| Early physical rehabilitation in intensive care patients with sepsis syndromes: a pilot randomised controlled trial | Kayambu 2015 | Low | Low | Low | High | Low | High |
| Early use of polymyxin B hemoperfusion in abdominal septic shock: the EUPHAS randomized controlled trial | Cruz 2009 | Low | High | Low | Low | Unsure | High |
| Early use of polymyxin B hemoperfusion in patients with septic shock due to peritonitis: a multicenter randomized control trial | Payen 2015 | Low | Low | High | Low | Low | High |
| Echocardiogram-guided resuscitation versus early goal-directed therapy in the treatment of septic shock: a randomized, controlled, feasibility trial | Lanspa 2018 | Low | Low | Low | Low | Low | High |
| Echocardiography-guided hemodynamic management of severe sepsis and septic shock in adults: a randomized controlled trial | Alhabashy 2021 | Low | High | Low | Unsure | Unsure | Unsure |
| Effect of a chimeric antibody to tumor necrosis factor-alpha on cytokine and physiologic responses in patients with severe sepsis - A randomized, clinical trial | Clark 1998 | Unsure | Low | Low | Low | High | High |
| Effect of a multifaceted educational intervention for anti-infectious measures on sepsis mortality: a cluster randomized trial | Bloos 2017 | Unsure | High | Low | Unsure | Low | High |
| Effect of A Probiotic Preparation on Gut Microbiota in Critically Ill Septic Patients Admitted to Intensive Care Unit: a Pilot Randomized Controlled Trial | Mahmoodpoor 2023 | Low | Low | Unsure | High | Unsure | High |
| Effect of a Recombinant Human Soluble Thrombomodulin on Mortality in Patients With Sepsis-Associated Coagulopathy: the SCARLET Randomized Clinical Trial | Vincent 2019 | Low | Low | Low | Low | Low | Unsure |
| Effect of a Resuscitation Strategy Targeting Peripheral Perfusion Status vs Serum Lactate Levels on 28-Day Mortality Among Patients With Septic Shock: the ANDROMEDA-SHOCK Randomized Clinical Trial | Hernandez 2019 | Low | High | Low | Low | Low | Low |
| Effect of an enteral diet enriched with eicosapentaenoic acid, gamma-linolenic acid and anti-oxidants on the outcome of mechanically ventilated, critically ill, septic patients | Grau-Carmona 2011 | Low | High | High | High | Low | High |
| Effect of an Herbal-Based Injection on 28-Day Mortality in Patients With Sepsis: The EXIT-SEP Randomized Clinical Trial. | Liu 2023 | Low | Low | Low | Low | Low | Low |
| Effect of antithrombin III supplementation on inflammatory response in patients with severe sepsis | Inthorn 1998 | Unsure | High | Low | Low | Unsure | High |
| Effect of Ascorbic Acid, Corticosteroids, and Thiamine on Organ Injury in Septic Shock: The ACTS Randomized Clinical Trial | Moskowitz 2020 | Low | Low | Low | Low | Low | Low |
| Effect of Cholecalciferol Supplementation on Vitamin D Status and Cathelicidin Levels in Sepsis: a Randomized, Placebo-Controlled Trial | Quraishi 2015 | Low | Low | Low | Low | Low | High |
| Effect of continuous blood purification combined with reduced glutathione on endotoxin, inflammatory mediators and severity of liver injury in patients with septic shock | Liu 2024 | Unsure | High | High | Low | Unsure | High |
| Effect of Continuous Infusion vs Bolus Dose of Hydrocortisone in Septic Shock: A Prospective Randomized Study. | Salhotra 2024 | Low | Low | Low | Low | Low | High |
| Effect of continuous renal replacement therapy on kidney injury molecule-1 and neutrophil gelatinase-associated lipocalin in patients with septic acute kidney injury | Shao 2017 | Unsure | High | Unsure | Low | Unsure | High |
| Effect of CRRT with oXiris filter on hemodynamic instability in surgical septic shock with AKI: a pilot randomized controlled trial | Feng 2022 | Unsure | High | High | Low | Low | High |
| Effect of Dexmedetomidine on Mortality and Ventilator-Free Days in Patients Requiring Mechanical Ventilation With Sepsis: a Randomized Clinical Trial | Kawazoe 2017 | Low | High | Low | Low | Low | Unsure |
| Effect of dexmedetomidine vs midazolam on the microcirculation of septic patients who are mechanically ventilated | MohamedAtefRefaat 2022 | Low | High | Low | Low | Low | High |
| Effect of Early Administration of Vitamin D on Clinical Outcome in Critically Ill Sepsis Patients: a Randomized Placebo-controlled Trial | Bhattacharyya 2021 | Low | Low | Low | Low | Unsure | Unsure |
| Effect of empirical treatment with moxifloxacin and meropenem vs meropenem on sepsis-related organ dysfunction in patients with severe sepsis: a randomized trial | Brunkhorst 2012 | Low | High | Low | Low | Low | Low |
| Effect of enteral diet enriched with eicosapentaenoic acid, gamma-linolenic acid, and antioxidants in patients with sepsis-induced acute respiratory distress syndrome | Shirai 2015 | Unsure | High | Unsure | Low | Low | High |
| Effect of eritoran, an antagonist of MD2-TLR4, on mortality in patients with severe sepsis: the ACCESS randomized trial | Opal 2013 | Low | Low | Low | Low | Low | Low |
| Effect of focused cardiopulmonary ultrasonography on clinical outcome of septic shock: a randomized study | Li 2021 | Unsure | High | Low | Low | Low | Unsure |
| Effect of Ganciclovir on IL-6 Levels Among Cytomegalovirus-Seropositive Adults With Critical Illness: a Randomized Clinical Trial | Limaye 2017 | Low | Low | Low | Low | Low | Low |
| Effect of glucose-insulin-potassium infusion on hemodynamics in patients with septic shock | Effat 2021 | Unsure | High | High | Unsure | High | High |
| Effect of granulocyte-monocyte colony-stimulating factor therapy on leukocyte function and clearance of serious infection in nonneutropenic patients | Rosenbloom 2005 | Low | High | High | Low | Unsure | High |
| Effect of heart rate control with esmolol on hemodynamic and clinical outcomes in patients with septic shock: a randomized clinical trial | Morelli 2013 | Unsure | High | High | Low | Low | High |
| Effect of high-dose Ascorbic acid on vasopressor's requirement in septic shock. | Zabet 2016 | Unsure | Low | Low | Low | Unsure | High |
| Effect of high-dose intravenous ascorbic acid on microcirculation and endothelial glycocalyx during sepsis and septic shock: a double-blind, randomized, placebo-controlled study | Belousoviene 2023 | Low | Low | Low | Unsure | High | High |
| Effect of Human Recombinant Alkaline Phosphatase on 7-Day Creatinine Clearance in Patients With Sepsis-Associated Acute Kidney Injury: a Randomized Clinical Trial | Pickkers 2018 | Low | Low | Low | Low | Low | Unsure |
| Effect of intravenous clarithromycin in patients with sepsis, respiratory and multiple organ dysfunction syndrome: a randomized clinical trial | Karakike 2022 | Low | Low | Low | Low | Low | Unsure |
| Effect of long-term and high-dose antithrombin supplementation on coagulation and fibrinolysis in patients with severe sepsis | Hoffmann 2004 | Unsure | High | High | High | Unsure | High |
| Effect of magnesium supplementation on lactate clearance in critically ill patients with severe sepsis: a randomized clinical trial | Noormandi 2020 | Unsure | High | Unsure | Low | Low | High |
| Effect of mode of hydrocortisone administration on glycemic control in patients with septic shock: a prospective randomized trial | Loisa 2007 | Low | High | High | Unsure | Low | Unsure |
| Effect of n-3 fatty acids on markers of brain injury and incidence of sepsis-associated delirium in septic patients | Burkhart 2014 | Low | High | Low | Low | Low | Unsure |
| Effect of Neutrophil Elastase Inhibitor (Sivelestat Sodium) on Oxygenation in Patients with Sepsis-Induced Acute Respiratory Distress Syndrome. | Wu 2025 | Low | Low | Low | Low | Low | Unsure |
| Effect of pentoxifylline in severe sepsis: results of a randomized, double-blind, placebo-controlled study | Staubach 1998 | Unsure | Low | Low | Low | Low | High |
| Effect of pentoxifylline on organ dysfunction and mortality in severe sepsis | Elgendy 2020 | Unsure | Low | Low | Low | Low | High |
| Effect of physostigmine on recovery from septic shock following intra-abdominal infection - Results from a randomized, double-blind, placebo-controlled, monocentric pilot trial (Anticholium R per Se). | Pinder 2019 | Low | Low | Low | Low | Low | High |
| Effect of Prolonged-Release Pirfenidone on Renal Function in Septic Acute Kidney Injury Patients: A Double-Blind Placebo-Controlled Clinical Trial | Chavez-Iniguez 2021 | Low | Low | Low | Low | Low | High |
| Effect of selenium supplementation on biochemical markers and outcome in critically ill patients | Mishra 2007 | Unsure | Low | Low | High | Unsure | High |
| Effect of Sitting Baduanjin exercise on early rehabilitation of sepsis patients with non-invasive ventilation : a randomized controlled trial. | Chen 2024 | Low | High | Low | Low | Low | High |
| Effect of small-dose levosimendan on mortality rates and organ functions in Chinese elderly patients with sepsis | Wang 2017 | Unsure | Low | Unsure | Low | Unsure | High |
| Effect of sodium selenite administration and procalcitonin-guided therapy on mortality in patients with severe sepsis or septic shock: a randomized clinical trial | Bloos 2016 | Low | Unsure | Low | Low | Low | Unsure |
| Effect of stress doses of hydrocortisone on S-100B vs. interleukin-8 and polymorphonuclear elastase levels in human septic shock | Mussack 2005 | Unsure | Low | Low | Low | Unsure | High |
| Effect of tanshinone iia on platelet parameters, coagulation function and blood lactate in patients with sepsis | Xia 2020 | Unsure | High | High | Low | Unsure | High |
| Effect of Targeted Polymyxin B Hemoperfusion on 28-Day Mortality in Patients With Septic Shock and Elevated Endotoxin Level: The EUPHRATES Randomized Clinical Trial | Dellinger 2018 | Low | Low | Low | Low | Low | Unsure |
| Effect of the antiendotoxic agent, taurolidine, in the treatment of sepsis syndrome: A placebo-controlled, double-blind trial (vol 23, pg 1033, 1995) | Willatts 1996 | Unsure | Low | Low | Low | Unsure | High |
| Effect of the Shenfu Injection Combined with Early Goal-Directed Therapy on Organ Functions and Outcomes of Septic Shock Patients | Li 2015 | Unsure | High | High | Low | Unsure | High |
| Effect of therapeutic drug monitoring-based dose optimization of piperacillin/tazobactam on sepsis-related organ dysfunction in patients with sepsis: a randomized controlled trial | Hagel 2022 | Low | High | Low | Low | Low | Low |
| Effect of Thiamine on Clinical Outcomes in Septic Shock Patients: a Randomized, Double-Blinded Pilot Study | Pereira 2023 | Low | Low | Low | High | Unsure | High |
| Effect of transcutaneous electrical muscle stimulation on muscle volume in patients with septic shock | Poulsen 2011 | Unsure | High | Low | Low | Low | High |
| Effect of treatment with low doses of hydrocortisone and fludrocortisone on mortality in patients with septic shock | Annane 2002 | Low | Low | Low | Low | Unsure | Unsure |
| Effect of two volume responsiveness evaluation methods on fluid resuscitation and prognosis in septic shock patients | Xu 2014 | Unsure | High | Low | Low | Unsure | High |
| Effect of Vitamin C Infusion on Organ Failure and Biomarkers of Inflammation and Vascular Injury in Patients With Sepsis and Severe Acute Respiratory Failure: the CITRIS-ALI Randomized Clinical Trial | Fowler 2019 | Low | Low | Low | Unsure | Low | Unsure |
| Effect of Vitamin C, Hydrocortisone, and Thiamine vs Hydrocortisone Alone on Time Alive and Free of Vasopressor Support Among Patients With Septic Shock: the VITAMINS Randomized Clinical Trial | Fujii 2020 | Low | High | Low | Low | Low | Low |
| Effect of Vitamin C, Thiamine, and Hydrocortisone on Ventilator- And Vasopressor-Free Days in Patients with Sepsis- And VICTAS Randomized Clinical Trial | Sevransky 2021 | Low | Low | Low | Low | Low | High |
| Effect on extrapulmonary sepsis-induced acute lung injury by hemoperfusion with neutral microporous resin column | Huang 2013 | Unsure | High | High | High | Unsure | High |
| Effectiveness and safety of Shenfu injection in septic patients with hypoperfusion: A multi-center, open-label, randomized, controlled trial. | Liu 2024 | Unsure | High | Low | Low | Low | Unsure |
| Effectiveness of enteral ivabradine for heart rate control in septic shock: A randomised controlled trial | Datta 2021 | Low | High | Low | Low | Low | Unsure |
| Effects of blood transfusion on oxygen transport variables in severe sepsis | Lorente 1993 | Unsure | High | High | Low | Unsure | High |
| Effects of capillary refill time-vs. lactate-targeted fluid resuscitation on regional, microcirculatory and hypoxia-related perfusion parameters in septic shock: a randomized controlled trial | Castro 2020 | Low | High | Unsure | Low | Low | High |
| Effects of carrimycin on biomarkers of inflammation and immune function in tumor patients with sepsis: a multicenter double-blind randomized controlled trial | Nan 2023 | Low | Low | Low | High | Low | High |
| Effects of comprehensive nursing on negative emotion and prognosis of patients with sepsis | Shen 2021 | Unsure | High | High | Low | Unsure | High |
| Effects of continuous haemofiltration <i>vs</i> intermittent haemodialysis on systemic haemodynamics and splanchnic regional perfusion in septic shock patients:: a prospective, randomized clinical trial | John 2001 | Unsure | High | High | Low | Unsure | High |
| Effects of continuous renal replacement therapy on inflammation-related anemia, iron metabolism and prognosis in sepsis patients with acute kidney injury | An 2023 | Low | High | Unsure | Unsure | Low | Unsure |
| Effects of corticotropin-releasing hormone on proopiomelanocortin derivatives and monocytic HLA-DR expression in patients with septic shock | Matejec 2013 | Unsure | Low | Low | Low | Unsure | High |
| Effects of crude rhubarb on intestinal permeability in septic patients | Fang 2007 | Unsure | High | High | High | Unsure | High |
| Effects of dexmedetomidine on renal function in patients with septic shock | Chen 2018 | Unsure | High | Unsure | Unsure | High | High |
| Effects of dobutamine on systemic, regional and microcirculatory perfusion parameters in septic shock. A randomized, placebo-controlled, double-blind, crossover study | Hernandez 2013 | Low | Low | Low | Low | Low | High |
| Effects of dopamine, norepinephrine, and epinephrine on the splanchnic circulation in septic shock: which is best? | DeBacker 2003 | Unsure | High | High | Low | Unsure | High |
| Effects of Early Continuous Venovenous Hemofiltration on E-Selectin, Hemodynamic Stability, and Ventilatory Function in Patients with Septic-Shock-Induced Acute Respiratory Distress Syndrome | Meng 2016 | Unsure | High | High | High | Unsure | High |
| Effects of early enteral nutrition on Th17/Treg cells and IL-23/IL-17 in septic patients | Sun 2019 | Low | High | Low | Low | Low | High |
| Effects of Early Use of Methylene Blue and Vasopressin on Noradrenaline Dose in Septic Shock: A Randomized Controlled Trial | Kuri 2025 | Low | High | Low | Low | Low | High |
| Effects of endotoxin adsorber hemoperfusion on sublingual microcirculation in patients with septic shock: a randomized controlled trial | Chen 2020 | Unsure | High | Low | Unsure | Low | High |
| Effects of enteral feeding with eicosapentaenoic acid, gamma-linolenic acid, and antioxidants in mechanically ventilated patients with severe sepsis and septic shock | Pontes-Arruda 2006 | Unsure | Low | Low | High | Unsure | High |
| Effects of epinephrine, norepinephrine, or the combination of norepinephrine and dobutamine on gastric mucosa in septic shock | Duranteau 1999 | Unsure | High | High | Low | Unsure | High |
| Effects of Esketamine Versus Remifentanil on Hemodynamics and Prognosis in Patients with Septic Shock Receiving Invasive Mechanical Ventilation: A Randomized Controlled Trial. | Li 2025 | Low | High | High | Low | Low | High |
| Effects of fluid resuscitation under the guidance of PICCO on the immune function and inflammatory mediator in patients with septic shock | Yao 2017 | Unsure | High | Unsure | Low | Unsure | High |
| Effects of high doses of selenium, as sodium selenite, in septic shock: a placebo-controlled, randomized, double-blind, phase II study | Forceville 2007 | Low | Low | Low | Low | Low | Unsure |
| Effects of high-dose of intravenous immunoglobulin and antibiotics on survival for severe sepsis undergoing surgery | Rodriguez 2005 | Low | Low | Low | Low | Unsure | High |
| Effects of hydrocortisone combined with vitamin C and vitamin B1 versus hydrocortisone alone on microcirculation in septic shock patients: a pilot study | Wang 2023 | Low | High | Low | High | Low | High |
| Effects of hydroxyethylstarch and gelatin on renal function in severe sepsis: a multicentre randomised study | Schortgen 2001 | Low | High | Low | Low | Low | Unsure |
| Effects of ibuprofen on the physiology and survival of hypothermic sepsis | Arons 1999 | Low | Low | Low | Low | Unsure | Unsure |
| Effects of IgM-enriched immunoglobulin therapy in septic-shock-induced multiple organ failure: pilot study | Toth 2013 | Unsure | High | Low | Low | Low | High |
| Effects of Increasing Hydrocortisone to 300 mg Per Day in the Treatment of Septic Shock: a Pilot Study | Hyvernat 2016 | Low | Low | Low | Low | Low | High |
| Effects of Levosimendan on Cellular Metabolic Alterations in Patients With Septic Shock: a Randomized Controlled Pilot Study | Hajjej 2017 | Unsure | Low | Low | Low | Unsure | High |
| Effects of levosimendan on mitochondrial function in patients with septic shock: A randomized trial | Torraco 2014 | Unsure | High | Low | Low | Low | High |
| Effects of levosimendan on right ventricular afterload in patients with acute respiratory distress syndrome: a pilot study | Morelli 2006 | Low | Low | Low | Low | Unsure | High |
| Effects of levosimendan on systemic and regional hemodynamics in septic myocardial depression | Morelli 2005 | Unsure | Low | Low | High | Unsure | High |
| Effects of lornoxicam on the physiology of severe sepsis | memis 2004 | Unsure | Low | Low | Low | Unsure | High |
| Effects of low-dose furosemide combined with aminophylline on the renal function in septic shock patients | Mai 2023 | Unsure | Low | Unsure | Low | Low | High |
| Effects of Melatonin and Propolis Supplementation on Inflammation, Oxidative Stress, and Clinical Outcomes in Patients with Primary Pneumosepsis: A Randomized Controlled Clinical Trial | Pahlavani 2022 | Unsure | Low | Low | Low | Low | High |
| Effects of N-acetylcysteine on microalbuminuria and organ failure in acute severe sepsis: results of a pilot study | Spapen 2005 | Unsure | Unsure | Unsure | Low | Low | High |
| Effects of nanocurcumin on inflammatory factors and clinical outcomes in critically ill patients with sepsis: a pilot randomized clinical trial | Karimi 2020 | Unsure | Low | Unsure | Low | Low | High |
| Effects of nitroglycerin on sublingual microcirculatory blood flow in patients with severe sepsis/septic shock after a strict resuscitation protocol: a double-blind randomized placebo controlled trial | Boerma 2010 | Low | Low | Low | Unsure | Unsure | High |
| Effects of norepinephrine, epinephrine, and norepinephrine-dobutamine on systemic and gastric mucosal oxygenation in septic shock | Zhou 2002 | Unsure | High | Low | Low | Unsure | High |
| Effects of parenteral glutamine supplementation on endocan levels in septic patients | Kilic 2021 | Unsure | High | High | Low | Unsure | High |
| Effects of pentoxifylline on circulating cytokine concentrations and hemodynamics in patients with septic shock: results from a double-blind, randomized, placebo-controlled study | Zeni 1996 | Unsure | High | Unsure | Low | Unsure | High |
| Effects of propofol and dexmedetomidine on indocyanine green elimination assessed with L√Ñ¬∞MON to patients with early septic shock: a pilot study | Memis 2009 | Unsure | High | High | Low | Unsure | High |
| Effects of recombinant human growth hormone in patients with severe sepsis | Voerman 1992 | Unsure | Low | Low | Unsure | Low | High |
| Effects of Shenfu Injection in the Treatment of Septic Shock Patients: a Multicenter, Controlled, Randomized, Open-Label Trial | Li 2016 | Unsure | High | High | Low | Low | High |
| Effects of Shenfu injection on sublingual microcirculation in septic shock patients: a randomized controlled trial | Wang 2022 | Low | Low | Low | Low | Unsure | High |
| Effects of short-term fenoldopam infusion on gastric mucosal blood flow in septic shock | Morelli 2004 | Unsure | Low | Low | Low | Unsure | High |
| Effects of short-term simultaneous infusion of dobutamine and terlipressin in patients with septic shock: the DOBUPRESS study | Morelli 2008 | Unsure | High | High | Low | Unsure | High |
| Effects of target temperature management on the outcome of septic patients with fever | Gao 2017 | Unsure | High | High | Low | Unsure | High |
| Effects of thiamine on balance between matrix metalloproteinases-9 (Mmp9) and tissue inhibitors of metalloproteinases-1 (timp-1) | Lubis 2021 | Unsure | Unsure | Unsure | High | Unsure | High |
| Effects of thiamine on vasopressor requirements in patients with septic shock: a prospective randomized controlled trial | Petsakul 2020 | Low | Low | Low | Low | Low | High |
| Effects of triple combination of hydrocortisone, thiamine, and Vitamin C on clinical outcome in patients with septic shock: a single-center randomized controlled trial | Jamshidi 2021 | Unsure | Unsure | Unsure | Low | Low | High |
| Effects of vasopressinergic receptor agonists on sublingual microcirculation in norepinephrine-dependent septic shock | Morelli 2011 | Unsure | Low | Low | Low | Low | High |
| Efficacy and Prognosis of HA380 Perfusion Combined with Continuous Venovenous Hemofiltration in the Treatment of Sepsis | Wang 2024 | Unsure | Unsure | Unsure | Low | Unsure | High |
| Efficacy and Safety of a Balanced Gelatine Solution for Fluid Resuscitation in Sepsis: A Prospective, Randomised, Controlled, Double-Blind Trial-GENIUS Trial. | Marx 2025 | Low | Low | Low | High | Low | High |
| Efficacy and safety of a phospholipid emulsion (GR270773) in Gram-negative severe sepsis: results of a phase II multicenter, randomized, placebo-controlled, dose-finding clinical trial | Dellinger 2009 | Low | Low | Low | Low | Low | Unsure |
| Efficacy and Safety of Continuous vs Intermittent Linezolid Infusion in Critically Ill Patients with Septic Shock | Albadry 2024 | Unsure | High | Unsure | Low | Unsure | Unsure |
| Efficacy and safety of dopamine versus norepinephrine in the management of septic shock | Patel 2010 | High | High | Low | Low | Low | Unsure |
| Efficacy and safety of landiolol, an ultra-short-acting beta1-selective antagonist, for treatment of sepsis-related tachyarrhythmia (J-Land 3S): a multicentre, open-label, randomised controlled trial. | Kakihana 2020 | Low | High | Low | Low | Low | High |
| Efficacy and safety of LY315920Na/S-5920, a selective inhibitor of 14-kDa group IIA secretory phospholipase A2, in patients with suspected sepsis and organ failure | Abraham 2003 | Low | Low | Low | Low | Unsure | Unsure |
| Efficacy and safety of procalcitonin guidance in reducing the duration of antibiotic treatment in critically ill patients: a randomised, controlled, open-label trial | deJong 2016 | Low | High | Low | Low | Low | Low |
| Efficacy and safety of recombinant human activated protein C for severe sepsis | Bernard 2001 | Low | Low | Low | Low | Low | High |
| Efficacy and Safety of Recombinant Human Thrombopoietin (rhTPO) on Coagulation Function and Inflammatory Factors in the Treatment of Patients with Sepsis-Related Thrombocytopenia. | Wang 2025 | Unsure | High | High | Low | Low | High |
| Efficacy and safety of the monoclonal anti-tumor necrosis factor antibody F(ab')2 fragment afelimomab in patients with severe sepsis and elevated interleukin-6 levels | Panacek 2004 | Low | Low | Low | Low | Unsure | Unsure |
| Efficacy and safety of the platelet-activating factor receptor antagonist BN 52021 (Ginkgolide B) in patients with severe sepsis: a randomised, double-blind, placebo-controlled, multicentre trial | Albrecht 2004 | Low | Low | Low | Low | Unsure | Unsure |
| Efficacy and safety of tifacogin (recombinant tissue factor pathway inhibitor) in severe sepsis: a randomized controlled trial | Abraham 2003 | Low | Low | Low | Low | Unsure | Unsure |
| Efficacy and Safety of Vilobelimab (IFX-1), a Novel Monoclonal Anti-C5a Antibody, in Patients With Early Severe Sepsis or Septic Shock-A Randomized, Placebo-Controlled, Double-Blind, Multicenter, Phase IIa Trial (SCIENS Study) | Bauer 2021 | Low | Low | Low | Low | Low | High |
| Efficacy of adjuvant use of midodrine in patients with septic shock: An open label randomized controlled trial. | El-Nagdy 2025 | Unsure | High | Low | Low | Low | Low |
| Efficacy of Continuous vs. Intermittent Administration of Cefepime in Adult ICU Patients with Gram-Negative Bacilli Bacteremia: A Randomized Double-Blind Clinical Study. | Alvarez-Moreno 2024 | Low | Low | Low | Low | Unsure | Unsure |
| Efficacy of coupled plasma filtration adsorption (CPFA) in patients with septic shock: a multicenter randomised controlled clinical trial | Livigni 2014 | Low | High | Low | Low | Low | High |
| Efficacy of Levosimendan in the Treatment of Patients With Severe Septic Cardiomyopathy | Sun 2023 | Unsure | High | Low | Low | Low | High |
| Efficacy of phenylephrine versus noradrenaline in management of patients presenting with septic shock in the intensive care unit | Hussain 2014 | Unsure | High | High | Low | Unsure | High |
| Efficacy of single-dose intravenous immunoglobulin administration for severe sepsis and septic shock | Hamano 2013 | Unsure | High | High | Low | Unsure | High |
| Efficacy of targeting high mean arterial pressure for older patients with septic shock (OPTPRESS): a multicentre, pragmatic, open-label, randomised controlled trial. | Endo 2025 | Low | High | Low | Low | Low | Low |
| Efficacy of ulinastatin combined with alanyglutamine for patients with sepsis | Yuan 2020 | Unsure | High | Unsure | Low | Unsure | High |
| Efficacy of xuebijing injection in the adjunctive therapy of acute respiratory distress syndrome caused by sepsis | Zhen 2019 | Unsure | High | Low | Low | Unsure | High |
| Electric Muscle Stimulation for Weaning from Mechanical Ventilation in Elder Patients with Severe Sepsis and Acute Respiratory Failure √¢¬Ä¬ì A Pilot Study | Shen 2017 | Unsure | High | High | Low | Unsure | Low |
| Electro-acupuncture attenuates inflammatory responses and intraabdominal pressure in septic patients: a randomized controlled trial | Meng 2018 | Unsure | High | High | Low | Unsure | High |
| Electroacupuncture Improves Intestinal Dysfunction in Septic Patients: a Randomised Controlled Trial | Meng 2018 | Unsure | High | High | High | Unsure | High |
| Elevation of cardiac output and oxygen delivery improves outcome in septic shock | Tuchschmidt 1992 | Unsure | High | Low | Low | Low | High |
| Empirical Micafungin Treatment and Survival Without Invasive Fungal Infection in Adults With ICU-Acquired Sepsis, <i>Candida</i> Colonization, and Multiple Organ Failure The EMPIRICUS Randomized Clinical Trial | EMPIRICUSTrialGrp 2016 | Low | Low | High | Low | Low | Low |
| Endotoxin and cytokin reduction function of the oXiris filter in a prospective double-blinded cross-over setting in patients with critical Gram-septic shock and continuous renal replacement therapy requiring acute kidney injury | Broman 2018 | Unsure | Low | Low | Unsure | Unsure | High |
| Endotoxin Removal in Septic Shock with the Alteco LPS Adsorber Was Safe But Showed no Benefit Compared to Placebo in the Double-Blind Randomized Controlled Trial-the Asset Study | Lipcsey 2020 | Low | Low | Low | Low | Low | High |
| Enoximone in contrast to dobutamine improves hepatosplanchnic function in fluid-optimized septic shock patients | Kern 2001 | Low | Low | Low | Low | Unsure | High |
| Enteral nutrition with eicosapentaenoic acid, gamma-linolenic acid and antioxidants in the early treatment of sepsis: results from a multicenter, prospective, randomized, double-blinded, controlled study: the INTERSEPT study. | Pontes-Arruda 2011 | Low | Low | Low | Low | Low | High |
| Enteral nutrition with omega-3 fatty acids in critically ill septic patients: a randomized double-blinded study | Ibrahim 2018 | Unsure | Low | Low | Low | Unsure | High |
| Evaluating the effect of dexmedetomidine on hemodynamic status of patients with septic shock admitted to intensive care unit: a single-blind randomized controlled trial | Gheibi 2020 | Unsure | High | Unsure | Low | Low | High |
| Evaluating the effects of curcumin nanomicelles on clinical outcome and cellular immune responses in critically ill sepsis patients: A randomized, double-blind, and placebo-controlled trial | Karimi 2022 | Low | Low | Low | Low | Low | High |
| Evaluating the effects of Esmolol on cardiac function in patients with Septic cardiomyopathy by Speck-tracking echocardiography-a randomized controlled trial | Wang 2023 | Unsure | High | Low | Low | Unsure | Unsure |
| Evaluating the efficacy and safety of two doses of the polyclonal anti-tumor necrosis factor-√é¬± fragment antibody AZD9773 in adult patients with severe sepsis and/or septic shock: randomized, double-blind, placebo-controlled phase IIb study* | Bernard 2014 | Low | Low | Low | Unsure | Low | High |
| Evaluating Vitamin C in Septic Shock: a Randomized Controlled Trial of Vitamin C Monotherapy | Wacker 2022 | Low | Low | Low | Low | Low | Unsure |
| Evaluation of endotoxin release and cytokine production induced by antibiotics in patients with Gram-negative nosocomial pneumonia | Maskin 2002 | Unsure | High | High | High | Unsure | High |
| Evaluation of Hydrocortisone, Vitamin C, and Thiamine for the Treatment of Septic Shock: a Randomized Controlled Trial (The HYVITS Trial) | Mohamed 2023 | Low | High | Low | High | Low | High |
| Evaluation of the Effects of a Combination of Vitamin C, Thiamine and Hydrocortisone vs Hydrocortisone Alone on ICU Outcome in Patients with Septic Shock: A Randomized Controlled Trial. | Sharma 2024 | Low | High | Low | Low | Unsure | High |
| Evaluation of the safety and efficacy of beta blockers in septic patients: a randomized control trial | Gadallah 2020 | Unsure | High | Low | Low | Unsure | High |
| Examination of setarud (IMOD√¢¬Ñ¬¢) in the management of patients with severe sepsis | Mahmoodpoor 2010 | Unsure | High | High | High | Unsure | High |
| Exploring choices of early nutritional support for patients with sepsis based on changes in intestinal microecology | Yang 2023 | Unsure | High | Unsure | Low | Unsure | High |
| Extended drotrecogin alfa (activated) treatment in patients with prolonged septic shock | Dhainaut 2009 | Low | Low | Low | Low | Unsure | High |
| Extracorporeal cytokine adsorption in septic shock: a proof of concept randomized, controlled pilot study | Hawchar 2019 | Unsure | High | High | Low | Low | High |
| Extravascular lung water monitoring of renal replacement therapy in lung water scavenging for septic acute kidney injury | Liu 2015 | Unsure | High | High | Low | Unsure | High |
| Feasibility Assessment of a Biomarker-Guided Kidney-Sparing Sepsis Bundle: The Limiting Acute Kidney Injury Progression In Sepsis Trial. | Gomez 2023 | Low | High | Unsure | Low | Low | High |
| Fever control using external cooling in septic shock: a randomized controlled trial | Schortgen 2012 | Low | High | Low | Low | Low | Unsure |
| Filgrastim in patients with pneumonia and severe sepsis or septic shock | Wunderink 2001 | Unsure | Low | Low | Low | Unsure | Unsure |
| Fluconazole improves survival in septic shock: a randomized double-blind prospective study | Jacobs 2003 | Unsure | Low | Unsure | Low | Unsure | High |
| Fludrocortisone dose-response relationship in septic shock: a randomised phase II trial. | Walsham 2024 | Low | High | High | Low | Low | High |
| Fluid Response Evaluation in Sepsis Hypotension and Shock: a Randomized Clinical Trial | Douglas 2020 | Low | High | Low | Low | Low | Unsure |
| Fluid resuscitation with colloids of different molecular weight in septic shock | Molnar 2004 | Unsure | High | High | Low | Unsure | High |
| Gastric tonometry versus cardiac index as resuscitation goals in septic shock: a multicenter, randomized, controlled trial | Palizas 2009 | Unsure | High | Low | Low | Low | Unsure |
| Glibenclamide dose response in patients with septic shock | Morelli 2007 | Unsure | Low | Low | Low | Unsure | High |
| Global end-diastolic volume index vs CVP goal-directed fluid resuscitation for COPD patients with septic shock: a randomized controlled trial | Yu 2017 | Unsure | High | Low | Low | Low | High |
| Granulocyte-macrophage colony-stimulating factor (GM-CSF) in patients presenting sepsis-induced immunosuppression: the GRID randomized controlled trial | Vacheron 2023 | Low | Low | Low | Low | Low | High |
| Granulocyte-macrophage colony-stimulating factor to reverse sepsis-associated immunosuppression: a double-blind, randomized, placebo-controlled multicenter trial | Meisel 2009 | Low | Low | Low | Low | Low | High |
| Hemodialysis membrane with a high-molecular-weight cutoff and cytokine levels in sepsis complicated by acute renal failure: a phase 1 randomized trial | Haase 2007 | Low | Low | Low | Low | Low | High |
| Hemodynamic effects of 6% and 10% hydroxyethyl starch solutions versus 4% albumin solution in septic patients | Friedman 2008 | Low | High | High | High | Unsure | High |
| Hemoperfusion using the LPS-selective mesoporous polymeric adsorbent in septic shock: a multicenter randomized clinical trial | Rey 2023 | Low | High | High | Low | Low | High |
| Heparanase inhibition leads to improvement in patients with acute gastrointestinal injuries induced by sepsis | Chen 2023 | Low | Low | Unsure | Unsure | Low | High |
| Heparanase inhibitor improves clinical study in patients with septic cardiomyopathy. | Chen 2024 | Unsure | Unsure | Unsure | Low | High | High |
| High versus Low Blood-Pressure Target in Patients with Septic Shock | Asfar 2014 | Low | High | Low | Low | Low | Low |
| High-dose antithrombin III in severe sepsis: A randomized controlled trial | Warren 2001 | Low | Low | Low | Low | Unsure | High |
| High-Dose IV Hydroxocobalamin (Vitamin B12) in Septic Shock: a Double-Blind, Allocation-Concealed, Placebo-Controlled Single-Center Pilot Randomized Controlled Trial (The Intravenous Hydroxocobalamin in Septic Shock Trial) | Patel 2023 | Low | Low | Low | Low | Low | High |
| High-Dose Versus Conventional-Dose Continuous Venovenous Hemodiafiltration and Patient and Kidney Survival and Cytokine Removal in Sepsis-Associated Acute Kidney Injury: A Randomized Controlled Trial | HICORESInvestigators 2016 | Low | High | Low | Low | Low | Low |
| High-dose vitamin C improves norepinephrine level in patients with septic shock: A single-center, prospective, randomized controlled trial. | Li 2024 | Low | Low | Low | High | Low | High |
| High-flow nasal oxygen cannula vs. noninvasive mechanical ventilation to prevent reintubation in sepsis: a randomized controlled trial | Tongyoo 2021 | Low | High | Low | Low | Low | Unsure |
| High-volume haemofiltration in human septic shock | Cole 2001 | Low | High | Low | Low | Low | Unsure |
| High-volume hemofiltration in adult burn patients with septic shock and acute kidney injury: a multicenter randomized controlled trial | Chung 2017 | Low | High | Unsure | High | Low | High |
| High-volume versus standard-volume haemofiltration for septic shock patients with acute kidney injury (IVOIRE study): a multicentre randomized controlled trial | Joannes-Boyau 2013 | Low | High | High | Low | Low | High |
| Hydrocortisone plus Fludrocortisone for Adults with Septic Shock | Annane 2018 | Low | Low | Low | Low | Low | Low |
| Hydrocortisone Therapy for Patients with Septic Shock | Sprung 2008 | Low | Low | Low | Low | Low | Low |
| Hydrocortisone treatment in early sepsis-associated acute respiratory distress syndrome: results of a randomized controlled trial | Tongyoo 2016 | Low | Low | Low | Low | Low | High |
| Hydrocortisone, vitamin C and thiamine for the treatment of sepsis and septic shock following cardiac surgery | Balakrishnan 2018 | Low | Low | Unsure | Low | Unsure | High |
| Hydroxyethyl starch 130/0.42 increased death at 90 days compared with Ringer's acetate in severe sepsis | Perner 2012 | Low | Low | Low | Low | Low | Low |
| Hyperglycemia Risk Evaluation of Hydrocortisone Intermittent Boluses versus Continuous Infusion in Septic Shock: A Prospective Randomized Trial | Ram 2022 | Unsure | High | Low | High | Unsure | High |
| Hyperoxia and hypertonic saline in patients with septic shock (HYPERS2S): a two-by-two factorial, multicentre, randomised, clinical trial | Asfar 2017 | Low | High | Low | Low | Low | Unsure |
| Hypertonic fluid administration in patients with septic shock: a prospective randomized controlled pilot study | vanHaren 2012 | Low | Low | Low | Low | Low | High |
| IgM-enriched immunoglobulins (Pentaglobin) may improve the microcirculation in sepsis: a pilot randomized trial | Domizi 2019 | Unsure | Low | Low | Unsure | Low | High |
| IgMA-enriched immunoglobulin in neutropenic patients with sepsis syndrome and septic shock: a randomized, controlled, multiple-center trial | Hentrich 2006 | Low | High | Low | Low | Low | Unsure |
| Iloprost and Organ Dysfunction in Adults With Septic Shock and Endotheliopathy: A Randomized Clinical Trial. | Bestle 2024 | Low | Low | Low | Unsure | Low | Unsure |
| Immune Checkpoint Inhibition in Sepsis: a Phase 1b Randomized, Placebo-Controlled, Single Ascending Dose Study of Antiprogrammed Cell Death-Ligand 1 Antibody (BMS-936559) | Hotchkiss 2019 | Low | Low | Low | Low | Low | High |
| Immunologic and hemodynamic effects of "low-dose" hydrocortisone in septic shock: a double-blind, randomized, placebo-controlled, crossover study | Keh 2003 | Low | Low | Low | Low | Low | High |
| Immunomodulatory effects of glutamine-enriched nutritional support in elderly patients with severe sepsis: a prospective, randomized, controlled study | Cai 2008 | High | High | Unsure | Low | High | High |
| Immunotherapy improves immune homeostasis and increases survival rate of septic patients | Huang 2009 | Unsure | High | High | Low | Unsure | High |
| Impact of continuous venovenous hemofiltration on organ failure during the early phase of severe sepsis: a randomized controlled trial | Payen 2009 | Unsure | High | Low | Low | Unsure | High |
| Impact of early versus late administration of norepinephrine on the hemodynamic outcome and mortality in septic shock | Elghareeb 2025 | Low | Low | Low | Low | Unsure | High |
| Impact of oral omega-3 fatty acids supplementation in early sepsis on clinical outcome and immunomodulation | Hosny 2013 | High | High | High | Low | Unsure | High |
| Impact of using midodrine for vasopressor weaning in septic shock patients: a randomized controlled trial | Mahmoud 2025 | Low | High | High | Low | Low | High |
| Impact of Very Early Physical Therapy During Septic Shock on Skeletal Muscle: a Randomized Controlled Trial | Hickmann 2018 | Unsure | High | Low | High | Low | High |
| Impact on fluid balance of an optimized restrictive strategy targeting non-resuscitative fluids in intensive care patients with septic shock: a single-blind, multicenter, randomized, controlled, pilot study. | Boulet 2024 | Low | High | Low | Low | Low | Unsure |
| Impacts of different hemofiltration methods on the prognosis of patients with sepsis | Ye 2017 | Unsure | High | Unsure | Low | Unsure | High |
| Improved sepsis bundles in the treatment of septic shock: a prospective clinical study | Lu 2015 | Unsure | High | High | Low | Unsure | High |
| Incidence of hypotension according to the discontinuation order of vasopressors in the management of septic shock: a prospective randomized trial (DOVSS) | Jeon 2018 | Low | Low | Low | Low | Low | High |
| Increasing mean arterial pressure in patients with septic shock: effects on oxygen variables and renal function | Bourgoin 2005 | High | High | Unsure | Low | High | High |
| Induced hypothermia in patients with septic shock and respiratory failure (CASS): a randomised, controlled, open-label trial | Itenov 2018 | Low | Low | Low | Low | Low | Low |
| Influence of angiotensin-converting enzyme inhibitor enalaprilat on endothelial-derived substances in the critically ill | Boldt 1998 | Unsure | Low | Low | Low | Unsure | High |
| Influence of liberal versus conservative oxygen therapies on the hemodynamic parameters of mechanically ventilated patients with sepsis: a randomized clinical trial. | Ghazaly 2024 | Low | High | Low | Low | Low | High |
| Influence of N-acetylcysteine on indirect indicators of tissue oxygenation in septic shock patients: results from a prospective, randomized, double-blind study | Spies 1994 | Unsure | Low | Low | Low | Low | High |
| Infusion of methylene blue in human septic shock: a pilot, randomized, controlled study | Kirov 2001 | Low | High | High | Low | Unsure | High |
| Initial evaluation of human recombinant interleukin-1 receptor antagonist in the treatment of sepsis syndrome: a randomized, open-label, placebo-controlled multicenter trial | Fisher 1994 | Low | High | Low | Low | Low | Unsure |
| Intensive insulin therapy and pentastarch resuscitation in severe sepsis | Brunkhorst 2008 | Low | High | Low | Low | Low | Low |
| Intensive insulin treatment improves forearm blood flow in critically ill patients: a randomized parallel design clinical trial | Zuran 2009 | Unsure | High | Low | Low | Low | Unsure |
| INTERSEPT: an international, multicenter, placebo-controlled trial of monoclonal antibody to human tumor necrosis factor-alpha in patients with sepsis. International Sepsis Trial Study Group | Cohen 1996 | Low | Low | Low | Low | High | High |
| Intravenous administration of ulinastatin (human urinary trypsin inhibitor) in severe sepsis: a multicenter randomized controlled study | Karnad 2014 | Low | Low | Low | Low | Low | High |
| Intravenous thiamine as an adjuvant therapy for hyperlactatemia in septic shock patients | Harun 2019 | Unsure | Low | Low | High | Low | High |
| Intravenous vitamin C administration to patients with septic shock: a pilot randomised controlled trial | Rosengrave 2022 | Low | Low | Low | Low | Low | High |
| Intravenous Vitamin C in Adults with Sepsis in the Intensive Care Unit | Lamontagne 2022 | Low | Low | Low | Low | Low | Low |
| Intravenously administered interleukin-7 to reverse lymphopenia in patients with septic shock: a double-blind, randomized, placebo-controlled trial | Daix 2023 | Low | Low | Low | Unsure | Low | High |
| Is continuous infusion ceftriaxone better than once-a-day dosing in intensive care? A randomized controlled pilot study | Roberts 2007 | Low | High | Low | Low | Unsure | High |
| Lactated Ringer's Versus 4% Albumin on Lactated Ringer's in Early Sepsis Therapy in Cancer Patients: a Pilot Single-Center Randomized Trial | Park 2019 | Low | Low | Low | Low | Low | Low |
| Landiolol and Organ Failure in Patients With Septic Shock: the STRESS-L Randomized Clinical Trial | Whitehouse 2023 | Low | High | Low | Low | Low | High |
| Landiolol for heart rate control in patients with septic shock and persistent tachycardia A multicenter Randomized Clinical Trial (Landi-SEP) | Rehberg 2024 | Low | High | High | Low | Low | Low |
| Lenercept (p55 tumor necrosis factor receptor fusion protein) in severe sepsis and early septic shock: a randomized, double-blind, placebo-controlled, multicenter phase III trial with 1,342 patients | Abraham 2001 | Unsure | Low | Low | Unsure | Unsure |  |
| Levosimendan for resuscitating the microcirculation in patients with septic shock: a randomized controlled study | Morelli 2010 | Unsure | Low | Low | Low | Low | High |
| Levosimendan for the prevention of acute organ dysfunction in sepsis | Gordon 2016 | Low | Low | Low | Low | Low | Low |
| Levosimendan Versus Dobutamine in Myocardial Injury Patients with Septic Shock: a Randomized Controlled Trial | Meng 2016 | Unsure | High | High | Low | Unsure | High |
| Liberal Versus Restrictive Transfusion Strategy in Critically Ill Oncologic Patients: the Transfusion Requirements in Critically Ill Oncologic Patients Randomized Controlled Trial | Bergamin 2017 | Low | High | Low | Low | Low | Unsure |
| Linezolid pharmacokinetic/pharmacodynamic profile in critically ill septic patients: intermittent versus continuous infusion | Adembri 2008 | Unsure | High | Low | Low | Unsure | Unsure |
| Long chain versus medium chain lipids in patients with ARDS: effects on pulmonary haemodynamics and gas exchange | Smirniotis 1998 | Unsure | High | Unsure | Low | Unsure | High |
| Low central venous pressure is not associated with low perfusion event in the setting of septic shock: a randomized controlled trial | George 2022 | Unsure | High | High | Low | Unsure | High |
| Low-dose hydrocortisone during severe sepsis: effects on microalbuminuria | Rinaldi 2006 | Unsure | High | Low | High | Unsure | High |
| Low-dose hydrocortisone improves shock reversal and reduces cytokine levels in early hyperdynamic septic shock | Oppert 2005 | Low | Low | Low | Low | Unsure | High |
| Low-dose hydrocortisone in patients with cirrhosis and septic shock: a randomized controlled trial | Arabi 2010 | Low | Low | Low | Low | Low | Unsure |
| Low-dose hydrocortisone treatment for patients with septic shock: a pilot study comparing 3days with 7days | Huh 2011 | Low | High | Low | Low | Unsure | High |
| Low-dose vasopressin in the treatment of vasodilatory septic shock | Malay 1999 | Low | Low | Low | Low | Low | Low |
| Lower versus higher hemoglobin threshold for transfusion in septic shock | Holst 2014 | Low | High | Low | Low | Low | Low |
| LY315920NA/S-5920, a selective inhibitor of group IIA secretory phospholipase A2, fails to improve clinical outcome for patients with severe sepsis | Zeiher 2005 | Low | Low | Low | Low | Low | High |
| Mega-dose sodium ascorbate: a pilot, single-dose, physiological effect, double-blind, randomized, controlled trial | Yanase 2023 | Low | Low | Low | Low | Low | High |
| Meropenem dosing in critically ill patients with sepsis and without renal dysfunction: intermittent bolus versus continuous administration? Monte Carlo dosing simulations and subcutaneous tissue distribution | Roberts 2009 | Low | High | Low | Low | Unsure | High |
| Metabolic effects of a D-beta-hydroxybutyrate infusion in septic patients: inhibition of lipolysis and glucose production but not leucine oxidation | Beylot 1994 | High | High | Unsure | Low | High | High |
| Metabolic resuscitation therapy in critically ill patients with sepsis and septic shock: a pilot prospective randomized controlled trial | Feng 2023 | Low | High | Low | Low | Low | High |
| Metabolic resuscitation using hydrocortisone ascorbic acid thiamine: Do individual components influence reversal of shock independently? | Reddy 2020 | High | High | High | Unsure | Unsure | High |
| Microcirculation properties of 20 % albumin in sepsis; a randomised controlled trial. | Cusack 2025 | Low | High | Low | Low | Low | Unsure |
| Microcirculatory effects of the transfusion of leukodepleted or non-leukodepleted red blood cells in patients with sepsis: a pilot study | Donati 2014 | Low | Low | Low | Low | Low | High |
| Midodrine improves clinical and economic outcomes in patients with septic shock: a randomized controlled clinical trial | Adly 2022 | Low | High | Unsure | Low | Unsure | Unsure |
| Mixed fibers diet in surgical ICU septic patients | Chittawatanarat 2010 | Low | Low | Low | Low | High | High |
| Multicenter evaluation of a human monoclonal antibody to Enterobacteriaceae common antigen in patients with Gram-negative sepsis | Albertson 2003 | Low | Low | Low | Low | Unsure | Unsure |
| Multicenter, double-blind, placebo-controlled study of the use of filgrastim in patients hospitalized with pneumonia and severe sepsis | Root 2003 | Low | Low | Low | Low | Unsure | Low |
| Multiple-center, randomized, placebo-controlled, double-blind study of the nitric oxide synthase inhibitor 546C88: effect on survival in patients with septic shock | Lopez 2004 | Low | Low | Low | Low | Low | High |
| Muscle wasting and function after muscle activation and early protocol-based physiotherapy: an explorative trial | Wollersheim 2019 | Unsure | High | Low | Unsure | Low | High |
| Muscle weakness in septic patients requiring mechanical ventilation: protective effect of transcutaneous neuromuscular electrical stimulation | Rodriguez 2012 | Unsure | High | Low | Low | Unsure | High |
| N-acetyl-L-cysteine depresses cardiac performance in patients with septic shock | Peake 1996 | Unsure | Low | Low | Low | Unsure | High |
| N-acetylcysteine increases liver blood flow and improves liver function in septic shock patients: results of a prospective, randomized, double-blind study | Rank 2000 | Unsure | Low | Low | Low | Unsure | High |
| N-acetylcysteine reduces respiratory burst but augments neutrophil phagocytosis in intensive care unit patients | Heller 2001 | Low | Low | Low | Low | Unsure | High |
| Nangibotide in patients with septic shock: a Phase 2a randomized controlled clinical trial | Francois 2020 | Low | Low | Low | Unsure | Low | High |
| Nano-curcumin supplementation in critically ill patients with sepsis: a randomized clinical trial investigating the inflammatory biomarkers, oxidative stress indices, endothelial function, clinical outcomes and nutritional status | Karimi 2022 | Low | Low | Low | Low | Low | High |
| Neuromuscular electrical stimulation acutely mobilizes endothelial progenitor cells in critically ill patients with sepsis | Stefanou 2016 | Unsure | High | Low | Low | Low | High |
| Neuromuscular Electrical Stimulator as a Protective Treatment against Intensive Care Unit Muscle Wasting in Sepsis/Septic Shock Patients | Cebeci 2022 | Low | High | Low | High | Low | High |
| Nocturnal urine 6-hydroxy sulfate melatonin is associated with the outcome of subjects with sepsis | Li 2022 | Low | High | Low | Low | Low | High |
| Norepinephrine in low to moderate doses may not increase luminal concentrations of L-lactate in the gut in patients with septic shock | Ibsen 2007 | Low | High | Unsure | Low | Unsure | High |
| Norepinephrine or dopamine for the treatment of hyperdynamic septic shock? | Martin 1993 | Unsure | Low | Low | Low | Unsure | High |
| Norepinephrine plus dobutamine versus epinephrine alone for management of septic shock: a randomised trial | Annane 2007 | Low | Low | Low | Low | Low | Unsure |
| Norepinephrine supplemented with dobutamine or epinephrine for the cardiovascular support of patients with septic shock | Mahmoud 2012 | Low | Low | Low | Low | Unsure | Unsure |
| Norepinephrine titration in patients with sepsis-induced encephalopathy: cerebral pulsatility index compared to mean arterial pressure guided protocol: randomized controlled trial. | Salem 2025 | Low | High | Low | Low | Low | Unsure |
| Norepinephrine weaning in septic shock patients by closed loop control based on fuzzy logic | Merouani 2008 | Unsure | High | High | Low | Unsure | High |
| Nursing Based on Humanistic Care Concept for Continuous Blood Purification for Patients with Severe Sepsis in the Intensive Care Unit. | Fan 2024 | High | High | High | Low | Unsure | High |
| Open randomized phase II trial of an extracorporeal endotoxin adsorber in suspected Gram-negative sepsis | Reinhart 2004 | Low | High | High | Low | Unsure | High |
| Optimizing left ventricular-arterial coupling during the initial resuscitation in septic shock - a pilot prospective randomized study | Zhou 2022 | Unsure | High | Low | Low | Unsure | High |
| Oral Midodrine Administration During the First 24 Hours of Sepsis to Reduce the Need of Vasoactive Agents: Placebo-Controlled Feasibility Clinical Trial. | Lal 2021 | Low | Low | Low | Low | Low | High |
| Outcome of ulinastatin vs metabolic resuscitation using ascorbic acid, thiamine and glucocorticoid in early treatment of sepsis-a randomised controlled trial | Yadav 2021 | Low | High | Low | Low | Unsure | High |
| Outcomes of High-Dose Versus Low-Dose Vitamin D on Prognosis of Sepsis Requiring Mechanical Ventilation: A Randomized Controlled Trial. | Ashoor 2024 | Low | High | Unsure | Unsure | Low | Unsure |
| Outcomes of Metabolic Resuscitation Using Ascorbic Acid, Thiamine, and Glucocorticoids in the Early Treatment of Sepsis: the ORANGES Trial | Iglesias 2020 | Low | Low | Low | Low | Low | Unsure |
| Oxygen delivery, oxygen consumption, and gastric intramucosal pH are not improved by a computer-controlled, closed-loop, vecuronium infusion in severe sepsis and septic shock | Freebairn 1997 | Unsure | High | High | Low | Unsure | High |
| P(v-a)CO2/C(a-v)O2-directed resuscitation does not improve prognosis compared with SvO2 in severe sepsis and septic shock: a prospective multicenter randomized controlled clinical study | Su 2018 | Unsure | High | Low | Low | Low | Unsure |
| Pancreatic enzyme replacement therapy can improve infection level nutrition condition and prognosis of patients with sepsis | Zhao 2025 | Unsure | Low | Low | High | Unsure | High |
| Parenteral administration of different amounts of branch-chain amino acids in septic patients: clinical and metabolic aspects | Garcia-de-Lorenzo 1997 | Low | High | High | Low | Unsure | High |
| Parenteral nutrition with fish oil modulates cytokine response in patients with sepsis | Mayer 2003 | Unsure | High | High | High | Unsure | High |
| Pentoxifylline and oxygen consumption in severe sepsis--a preliminary report | Castanon-Gonzalez 1995 | Unsure | Low | Unsure | Low | High | High |
| Pharmacokinetic and Pharmacodynamic Efficacies of Continuous versus Intermittent Administration of Meropenem in Patients with Severe Sepsis and Septic Shock: a Prospective Randomized Pilot Study | Zhao 2017 | Unsure | High | High | Low | Unsure | High |
| Pharmacokinetic evaluation of meropenem and imipenem in critically ill patients with sepsis | Novelli 2005 | Unsure | High | Unsure | Low | Low | High |
| Pharmacokinetics and Pharmacodynamics of Linezolid in Patients With Sepsis Receiving Continuous Venovenous Hemofiltration and Extended Daily Hemofiltration | Zheng 2020 | Unsure | High | Low | Low | Unsure | High |
| Pharmacokinetics of piperacillin in critically ill patients receiving continuous venovenous haemofiltration: a randomised controlled trial of continuous infusion versus intermittent bolus administration | Jamal 2015 | Low | High | High | Low | Low | High |
| Phase 2 trial of eritoran tetrasodium (E5564), a toll-like receptor 4 antagonist, in patients with severe sepsis | Tidswell 2010 | Low | Low | Low | Low | Low | Unsure |
| Phase 3 Pilot Randomized Controlled Trial Comparing Early Trophic Enteral Nutrition With "No Enteral Nutrition" in Mechanically Ventilated Patients With Septic Shock | Patel 2020 | Low | High | High | Low | Low | High |
| Phase I safety trial of intravenous ascorbic acid in patients with severe sepsis | Fowler 2014 | Low | Low | Low | Unsure | Low | High |
| Phase II multicenter clinical study of the platelet-activating factor receptor antagonist BB-882 in the treatment of sepsis | Vincent 2000 | Low | Low | Low | Low | Low | Unsure |
| Phase-3 trial of recombinant human alkaline phosphatase for patients with sepsis-associated acute kidney injury (REVIVAL) | Pickkers 2024 | Low | Low | Low | Low | Low | High |
| Phenylephrine versus norepinephrine for initial hemodynamic support of patients with septic shock: a randomized, controlled trial | Morelli 2008 | Unsure | Low | Low | Low | Low | High |
| Physiological-dose steroid therapy in sepsis | Yildiz 2002 | Unsure | Low | Low | Low | Unsure | High |
| Pilot study on the effects of high cutoff hemofiltration on the need for norepinephrine in septic patients with acute renal failure | Morgera 2006 | Unsure | High | High | High | Unsure | High |
| Piperacillin penetration into tissue of critically ill patients with sepsis--bolus versus continuous administration? | Roberts 2009 | Low | High | Low | Low | Unsure | High |
| Plasma as endothelial rescue in septic shock: A randomized, phase 2a pilot trial. | Clausen 2024 | Low | High | Low | High | Low | High |
| Platelet-activating factor receptor antagonist BN 52021 in the treatment of severe sepsis: A randomized, double-blind, placebo-controlled, multicenter clinical trial | Dhainaut 1994 | Unsure | Low | Low | Low | Unsure | Unsure |
| Polymyxin B therapy based on therapeutic drug monitoring in carbapenem-resistant organisms sepsis: the PMB-CROS randomized clinical trial | Liu 2023 | Unsure | High | Low | Low | Low | Unsure |
| Polymyxin-B hemoperfusion inactivates circulating proapoptotic factors | Cantaluppi 2008 | High | High | Unsure | Low | High | High |
| Positive effect of septimeb√¢¬Ñ¬¢ on mortality rate in severe sepsis: a novel non antibiotic strategy | Eslami 2012 | Unsure | High | High | Low | Unsure | High |
| Pre-emptive hydrocortisone therapy in early septic shock: a double-blind, allocation-concealed, pilot randomized controlled trial. | Emami 2025 | Low | Low | Low | Low | Low | High |
| Preload dependence indices to titrate volume expansion during septic shock: a randomized controlled trial | Richard 2015 | Low | High | Low | Low | High | High |
| Procalcitonin (PCT)-guided algorithm reduces length of antibiotic treatment in surgical intensive care patients with severe sepsis: results of a prospective randomized study | Schroeder 2009 | Unsure | High | Low | Low | Low | High |
| Procalcitonin algorithm in critically ill adults with undifferentiated infection or suspected sepsis. A randomized controlled trial | Shehabi 2014 | Low | High | Low | Low | Low | Low |
| Procalcitonin to guide duration of antibiotic therapy in intensive care patients: a randomized prospective controlled trial | Hochreiter 2009 | Unsure | High | High | Low | Low | High |
| Procalcitonin versus C-reactive protein for guiding antibiotic therapy in sepsis: a randomized trial | Oliveira 2013 | Low | High | High | Low | Low | High |
| Procalcitonin-Guided Management and Duration of Antibiotic Therapy in Critically Ill Cancer Patients With Sepsis (Pro-Can Study): A Randomized Controlled Trial. | Nazer 2024 | Low | High | Low | Low | Low | High |
| Procalcitonin-Guided Treatment on Duration of Antibiotic Therapy and Cost in Septic Patients (PRODA): a Multi-Center Randomized Controlled Trial | Jeon 2019 | Low | High | Low | Low | Low | Unsure |
| Prophylactic fenoldopam for renal protection in sepsis: a randomized, double-blind, placebo-controlled pilot trial | Morelli 2005 | Low | Low | Low | Low | Unsure | High |
| Prophylactic heparin in patients with severe sepsis treated with drotrecogin alfa (activated) | Levi 2007 | Low | Low | Low | Low | Low | High |
| Propofol increases preload dependency in septic shock patients | Yu 2015 | Unsure | High | High | Low | Low | High |
| Prospective evaluation of the efficacy, safety, and optimal biomarker enrichment strategy for nangibotide, a TREM-1 inhibitor, in patients with septic shock (ASTONISH): a double-blind, randomised, controlled, phase 2b trial | Francois 2023 | Low | Low | Low | Low | Low | Unsure |
| Protective effect of rhubarb combined with ulinastatin for patients with sepsis | Meng 2020 | Unsure | High | High | High | Unsure | High |
| Protective effect of Xuebijing injection on myocardial injury in patients with sepsis: a randomized clinical trial | Zhang 2016 | Unsure | Low | Low | Low | Unsure | High |
| Protein C zymogen in severe sepsis: a double-blinded, placebo-controlled, randomized study | Pappalardo 2016 | Low | Low | Low | Low | Low | High |
| Protocolized reduction of non-resuscitation fluids versus usual care in septic shock patients (REDUSE): a randomized multicentre feasibility trial. | Linden 2024 | Low | High | Low | Low | Low | Low |
| Randomized Clinical Trial of Antioxidant Therapy Patients with Septic Shock and Organ Dysfunction in the ICU: SOFA Score Reduction by Improvement of the Enzymatic and Non-Enzymatic Antioxidant System | Aisa-Alvarez 2023 | Low | Unsure | Low | Low | Unsure | Unsure |
| Randomized controlled clinical trial evaluating multiplex polymerase chain reaction for pathogen identification and therapy adaptation in critical care patients with pulmonary or abdominal sepsis | Tafelski 2015 | Low | Low | Low | Low | Low | Unsure |
| Randomized controlled trial of calcitriol in severe sepsis | Leaf 2014 | Low | Low | Low | Low | Low | High |
| Randomized trial evaluating serial protein C levels in severe sepsis patients treated with variable doses of drotrecogin alfa (activated) | Shorr 2010 | Low | Low | Low | Low | Low | High |
| Randomized, double blind, placebo-controlled trial of fish-oil-based lipid emulsion infusion for treatment of critically ill patients with severe sepsis | Khor 2011 | Low | Low | Low | Low | Unsure | High |
| Randomized, double-blind, placebo-controlled crossover pilot study of a potassium channel blocker in patients with septic shock | Warrillow 2006 | Unsure | Low | Low | Low | Unsure | High |
| Randomized, double-blind, placebo-controlled trial of granulocyte colony-stimulating factor in patients with septic shock | Stephens 2008 | Low | Low | Low | Low | Low | Unsure |
| Randomized, placebo-controlled trial of acetaminophen for the reduction of oxidative injury in severe sepsis: the Acetaminophen for the Reduction of Oxidative Injury in Severe Sepsis trial | Janz 2015 | Low | Low | Low | Unsure | Low | High |
| Randomized, placebo-controlled trial of the anti-tumor necrosis factor antibody fragment afelimomab in hyperinflammatory response during severe sepsis: the RAMSES Study | Reinhart 2001 | Low | Low | Low | Low | Unsure | High |
| Ranitidine is unable to maintain gastric pH levels above 4 in septic patients | TerziCoelho 2009 | Unsure | High | Low | Low | Low | High |
| Recombinant human activated protein C for adults with septic shock: a randomized controlled trial | Annane 2013 | Low | Low | Low | Low | Low | Unsure |
| Recombinant human interleukin 1 receptor antagonist in the treatment of patients with sepsis syndrome. Results from a randomized, double-blind, placebo-controlled trial. Phase III rhIL-1ra Sepsis Syndrome Study Group | Fisher 1994 | Low | Low | Low | Low | Unsure | High |
| Recombinant human platelet-activating factor acetylhydrolase for treatment of severe sepsis: results of a phase III, multicenter, randomized, double-blind, placebo-controlled, clinical trial | Opal 2004 | Low | Low | Low | Low | Unsure | Low |
| Recombinant platelet-activating factor acetylhydrolase to prevent acute respiratory distress syndrome and mortality in severe sepsis: phase IIb, multicenter, randomized, placebo-controlled, clinical trial | Schuster 2003 | Low | Low | Low | Low | Low | Unsure |
| Red blood cell transfusion does not increase oxygen consumption in critically ill septic patients | Fernandes 2001 | Unsure | High | High | Low | Unsure | High |
| Remote ischemic conditioning in septic shock: the RECO-Sepsis randomized clinical trial | Cour 2022 | Low | High | Low | Low | Low | Unsure |
| Removal of humoral mediators and the effect on the survival of septic patients by hemoperfusion with neutral microporous resin column | Huang 2010 | Unsure | High | Low | Unsure | High | High |
| Renal protective effect and clinical analysis of vitamin B6 in patients with sepsis | Wang 2024 | Unsure | Unsure | Unsure | Low | Low | Unsure |
| Restricting volumes of resuscitation fluid in adults with septic shock after initial management: the CLASSIC randomised, parallel-group, multicentre feasibility trial | Hjortrup 2016 | Low | High | Low | Low | Low | High |
| Restriction of Intravenous Fluid in ICU Patients with Septic Shock | Meyhoff 2022 | Low | High | Low | Low | Low | Low |
| Results of a pilot study on the effects of propofol and dexmedetomidine on inflammatory responses and intraabdominal pressure in severe sepsis | Tasdogan 2009 | Unsure | High | Low | Low | Unsure | High |
| Reversal of late septic shock with supraphysiologic doses of hydrocortisone | Bollaert 1998 | High | High | Unsure | Low | High | High |
| Right dose, right now: bedside, real-time, data-driven, and personalised antibiotic dosing in critically ill patients with sepsis or septic shock-a two-centre randomised clinical trial | Roggeveen 2022 | Low | High | Low | Low | Low | High |
| Role of dexmedetomidine in modifying immune paralysis in patients with septic shock: randomized controlled trial | Elayashy 2023 | Low | Low | Low | Low | Low | High |
| Role of edaravone in managemant of septic peritonitis | Elbaradey 2016 | Low | High | Low | Low | Unsure | High |
| Role of IV Vitamin C in Treatment and Prognosis of Sepsis | Shakya 2024 | Unsure | High | High | Low | Unsure | High |
| Roles of small-dose recombinant human brain natriuretic peptide without bolus in Chinese older patients with septic cardiac dysfunction | Zhang 2019 | Unsure | High | Low | Low | Unsure | High |
| Rosuvastatin for sepsis-associated acute respiratory distress syndrome | Lung 2014 | Low | Low | Low | Low | Low | Low |
| Safety and efficacy of affinity-purified, anti-tumor necrosis factor-alpha, ovine fab for injection (CytoFab) in severe sepsis | Rice 2006 | Low | Low | Low | Low | Low | High |
| Safety and Efficacy of Vitamin C, Vitamin B1, and Hydrocortisone in clinical outcome of septic shock receiving standard care: a quasi experimental randomized open label two arm parallel group study | Raghu 2021 | Low | Low | Low | Low | Low | Low |
| Safety and tolerability of non-neutralizing adrenomedullin antibody adrecizumab (HAM8101) in septic shock patients: the AdrenOSS-2 phase 2a biomarker-guided trial | Laterre 2021 | Low | Low | Low | Low | Low | High |
| Safety and vasopressor effect of rosuvastatin in septic patients | ElGendy 2014 | Low | Low | Low | Low | Unsure | High |
| Safety of megadose meropenem in the empirical treatment of nosocomial sepsis: a pilot randomized clinical trial | Salehi 2023 | Unsure | High | Low | High | Unsure | High |
| Safety, tolerability, pharmacokinetics, and efficacy of kukoamine B in patients with sepsis: a randomized phase IIa trial | Hu 2023 | Low | Low | Low | Low | Low | High |
| Score-based immunoglobulin G therapy of patients with sepsis: the SBITS study | Werdan 2007 | Low | Low | Low | Low | Low | High |
| Selepressin, a novel selective vasopressin V1A agonist, is an effective substitute for norepinephrine in a phase IIa randomized, placebo-controlled trial in septic shock patients | Russell 2017 | Low | Low | Low | Low | Low | High |
| Serum IL-6 and IL-1-ra with sequential organ failure assessment scores in septic patients receiving high-volume haemofiltration and continuous venovenous haemofiltration | Ghani 2006 | Unsure | High | High | High | Unsure | High |
| Serum procalcitonin as a biomarker to determine the duration of antibiotic therapy in adult patients with sepsis and septic shock in intensive care units: a prospective study | Vishalashi 2021 | Unsure | High | High | Low | Low | High |
| Shenfu injection for improving cellular immunity and clinical outcome in patients with sepsis or septic shock | Zhang 2017 | Unsure | Low | Low | Low | Unsure | High |
| Single-blinded, randomized, and controlled clinical trial evaluating the effects of Omega-3 fatty acids among septic patients with intestinal dysfunction: a pilot study | Chen 2017 | Unsure | High | Unsure | Low | High | High |
| Soluble fiber reduces the incidence of diarrhea in septic patients receiving total enteral nutrition: a prospective, double-blind, randomized, and controlled trial | Spapen 2001 | Unsure | Low | Low | High | Low | High |
| Solumedrol Treatment for Severe Sepsis in Humans with a Blunted Adrenocorticotropic Hormone-Cortisol Response: a Prospective Randomized Double-Blind Placebo-Controlled Pilot Clinical Trial | Birudaraju 2022 | Low | Low | Unsure | High | High | High |
| StO2 guided early resuscitation in subjects with severe sepsis or septic shock: a pilot randomised trial | Nardi 2013 | Low | High | Unsure | Low | Low | High |
| Stress doses of hydrocortisone in septic shock: beneficial effects on opsonization-dependent neutrophil functions | Kaufmann 2008 | Unsure | Low | Low | Low | Low | High |
| Stress doses of hydrocortisone reverse hyperdynamic septic shock: a prospective, randomized, double-blind, single-center study | Briegel 1999 | High | High | Unsure | Low | High | High |
| Talactoferrin in Severe Sepsis: results From the Phase II/III Oral tAlactoferrin in Severe sepsIS Trial | Vincent 2015 | Low | Low | Low | Low | Low | High |
| Targeted Fluid Minimization Following Initial Resuscitation in Septic Shock: a Pilot Study | Chen 2015 | Unsure | High | Unsure | Low | Low | High |
| Targeting matrix metalloproteinases with intravenous doxycycline in severe sepsis--A randomised placebo-controlled pilot trial | Nukarinen 2015 | Low | Low | Low | Low | Low | High |
| Targeting skeletal muscle tissue oxygenation (StO2) in adults with severe sepsis and septic shock: a randomised controlled trial (OTO-StS Study) | Nardi 2018 | Low | High | High | Low | Low | High |
| Terlipressin or norepinephrine in hyperdynamic septic shock: a prospective, randomized study | Albanese 2005 | Unsure | High | Low | Low | Unsure | Unsure |
| Terlipressin versus norepinephrine as infusion in patients with septic shock: a multicentre, randomised, double-blinded trial | Liu 2018 | Low | Low | Low | Low | Low | Unsure |
| Testing a conceptual model on early opening of the microcirculation in severe sepsis and septic shock <i>A randomised controlled pilot study</i> | vanderVoort 2015 | Unsure | High | High | High | Low | High |
| The clinical and paraclinical effectiveness of four-hour infusion vs. half-hour infusion of high-dose ampicillin-sulbactam in treatment of critically ill patients with sepsis or septic shock: an assessor-blinded randomized clinical trial | Mirjalili 2023 | Low | Low | Low | High | Low | High |
| The clinical effectiveness of sivelestat in treating sepsis patients with both acute respiratory distress syndrome and septic cardiomyopathy. | Lv 2024 | Unsure | High | Unsure | Low | Low | High |
| The contrasting effects of dobutamine and dopamine on gastric mucosal perfusion in septic patients | Neviere 1996 | | Unsure | High | Unsure | Low | Low |
| The contrasting effects of dopamine and norepinephrine on systemic and splanchnic oxygen utilization in hyperdynamic sepsis | Marik 1994 | Unsure | High | High | Low | Unsure | High |
| The effect of a novel extracorporeal cytokine hemoadsorption device on IL-6 elimination in septic patients: A randomized controlled trial | Schadler 2017 | Unsure | High | Unsure | High | Low | High |
| The effect of atrial natriuretic peptide infusion on intestinal injury in septic shock | Elbaradey 2016 | Low | High | Low | Low | Unsure | High |
| The effect of body position changes on stroke volume variation in 66 mechanically ventilated patients with sepsis | Daihua 2012 | Unsure | High | High | Low | Unsure | Unsure |
| The effect of continuous infusion of meropenem antibiotic on clinical signs and changes of procalcitonin in patients with acute sepsis related to cancer | Farokhi 2020 | Unsure | Low | Unsure | Unsure | Unsure | High |
| The effect of glutathione and N-acetylcysteine on lipoperoxidative damage in patients with early septic shock | Ortolani 2000 | Unsure | Unsure | Low | High | Unsure | High |
| The effect of N-acetylcysteine on nuclear factor-kappa B activation, interleukin-6, interleukin-8, and intercellular adhesion molecule-1 expression in patients with sepsis | Paterson 2003 | Low | Low | Low | High | Unsure | High |
| The effect of Na-selenite treatment on the oxidative stress-antioxidants balance of multiple organ failure | Woth 2014 | Unsure | High | Unsure | Low | Unsure | High |
| The effect of polymyxin B hemoperfusion on modulation of human leukocyte antigen DR in severe sepsis patients | Srisawat 2018 | Low | High | Low | Low | Low | High |
| The Effect of Xinmailong Infusion on Sepsis-Induced Myocardial Dysfunction: a Pragmatic Randomized Controlled Trial | He 2021 | Low | Low | Low | Low | Low | Unsure |
| The Effects of Atorvastatin on Inflammatory Responses and Mortality in Septic Shock: a Single-center, Randomized Controlled Trial | Singh 2017 | Low | Low | Low | Low | Low | High |
| The Effects of Genistein as Supplement to Oral/ Enteral Nutrition on Inflammatory Cytokines in Septic ICU patients: a Prospective, Single-center, Randomized Controlled Pilot Study | Elay 2023 | Unsure | High | High | Low | Unsure | High |
| The effects of hydroxyethyl starch solution in critically ill patients | Palumbo 2006 | Unsure | Unsure | Low | Low | Unsure | High |
| The effects of hypertonic fluid administration on the gene expression of inflammatory mediators in circulating leucocytes in patients with septic shock: a preliminary study. | vanHaren 2011 | Low | Low | Low | Low | Low | High |
| The effects of ibuprofen on the physiology and survival of patients with sepsis. The Ibuprofen in Sepsis Study Group | Bernard 1997 | Low | Low | Low | Low | Unsure | Unsure |
| The effects of IgM-enriched immunoglobulin preparations in patients with severe sepsis [ISRCTN28863830] | Tugrul 2002 | Unsure | High | Unsure | Unsure | Low | High |
| The effects of intravenous antioxidants in patients with septic shock | Galley 1997 | Unsure | Low | Unsure | Low | Unsure | High |
| The effects of L-carnitine supplementation on inflammation, oxidative stress, and clinical outcomes in critically Ill patients with sepsis: a randomized, double-blind, controlled trial | Keshani 2024 | Low | Low | Low | Low | Low | High |
| The effects of levosimendan vs dobutamine added to dopamine on liver functions assessed with noninvasive liver function monitoring in patients with septic shock | Memis 2012 | Unsure | High | High | Low | Unsure | High |
| The effects of moderate-dose steroid therapy in sepsis: A placebo-controlled, randomized study | Yildiz 2011 | Unsure | Low | Low | Low | Low | Unsure |
| The effects of saffron supplementation on inflammation and hematological parameters in patients with sepsis: a randomized controlled trial. | Hassanizadeh 2025 | Low | Low | Low | High | Low | High |
| The efficacy and safety of thymosin alpha1 for sepsis (TESTS): multicentre, double blinded, randomised, placebo controlled, phase 3 trial. | Wu 2025 | Low | Low | Low | Low | Low | Low |
| The efficacy of modified HuangLian JieDu decoction for early enteral nutrition in patients with sepsis: a randomized controlled study | Wang 2022 | Unsure | High | Low | Low | Low | High |
| The efficacy of thymosin alpha 1 for severe sepsis (ETASS): a multicenter, single-blind, randomized and controlled trial | Wu 2013 | Low | High | Low | Low | Low | Unsure |
| The immunological benefit of higher dose N-acetyl cysteine following mechanical ventilation in critically ill patients | Najafi 2014 | Unsure | High | High | High | Unsure | High |
| The impact of an omega-3 fatty acid rich lipid emulsion on fatty acid profiles in critically ill septic patients | Hall 2016 | Unsure | High | High | High | Unsure | High |
| The impact of lactate-buffered high-volume hemofiltration on acid-base balance | Cole 2003 | Unsure | High | Unsure | Low | High | High |
| The influence of alpha-tocopherol on cytokine levels and gastric intramucosal pH in severe sepsis | Memis 2008 | Unsure | Low | Low | Low | Unsure | High |
| The influence of methylene blue infusion on cytokine levels during severe sepsis | Memis 2002 | Unsure | Low | Low | Low | Unsure | High |
| The influence of N-acetyl cysteine infusion oncytokine levels and gastric intramucosal pH during severe sepsis | Emet 2004 | Unsure | Low | Low | Low | Unsure | High |
| The premature closure of ROMPA clinical trial: mortality reduction in septic shock by plasma adsorption | Gimenez-Esparza 2019 | Unsure | High | Low | High | Low | High |
| The protective effect of ulinastatin in severe sepsis. A mechanistic approach | Zhou 2020 | High | High | Low | Low | Unsure | High |
| The Restrictive IV Fluid Trial in Severe Sepsis and Septic Shock (RIFTS): a Randomized Pilot Study | Corl 2019 | Low | High | Low | Low | Low | Unsure |
| Therapeutic effects of curcumin and piperine combination in critically ill patients with sepsis: a randomized double-blind controlled trial. | Alikiaii 2025 | Low | Low | Low | Low | Low | Unsure |
| Therapeutic Hyperthermia Is Associated With Improved Survival in Afebrile Critically Ill Patients With Sepsis: a Pilot Randomized Trial | Drewry 2022 | Low | High | Low | Low | Low | High |
| Therapeutic Plasma Exchange Protects Patients with Sepsis-Associated Disseminated Intravascular Coagulation by Improving Endothelial Function | Weng 2021 | Unsure | High | Low | Low | Unsure | High |
| Thiamine for Renal Protection in Septic Shock (TRPSS): a Randomized, Placebo-controlled, Clinical Trial | Moskowitz 2023 | Low | Low | Low | Low | Low | Low |
| Time course of organ failure in patients with septic shock treated with hydrocortisone: results of the Corticus study | Moreno 2011 | Low | Low | Low | Low | Low | Low |
| Timing of Renal-Replacement Therapy in Patients with Acute Kidney Injury and Sepsis | Barbar 2018 | Low | High | Low | Low | Low | Unsure |
| Traditional Chinese medicine bundle therapy for septic acute gastrointestinal injury: a multicenter randomized controlled trial | Xing 2019 | Low | High | Low | Low | Low | High |
| Treatment of patients with severe sepsis using ulinastatin and thymosin alpha1: a prospective, randomized, controlled pilot study | Chen 2009 | Unsure | Unsure | Unsure | Low | High | High |
| Treatment of septic shock with the tumor necrosis factor receptor: fc fusion protein. The Soluble TNF Receptor Sepsis Study Group | Fisher 1996 | Low | Low | Low | Low | Unsure | Low |
| Triiodothyronine hormone supplementation therapy in septic shock patients with euthyroid sick syndrome: two pilot, placebo-controlled, randomized trials. | Kovacevic 2004 | Low | Low | Low | Low | Low | High |
| Ubiquinol (reduced coenzyme Q10) in patients with severe sepsis or septic shock-a randomized, double-blind, placebo-controlled pilot trial | Donnino 2015 | Low | Low | Low | Low | Low | High |
| Ultrasound-Guided Fluid Volume Management in Patients With Septic Shock: A Randomized Controlled Trial. | Li 2025 | Low | Low | High | Low | Unsure | Unsure |
| Use of heptaminol hydrochloride for catecholamine weaning in septic shock | Bahloul 2012 | High | High | High | Low | Unsure | High |
| Use of military anti-shock trousers in resuscitation of acute circulatory failure patients in intensive care unit | Boontoterm 2022 | Low | High | Unsure | Low | High | High |
| Use of procalcitonin to shorten antibiotic treatment duration in septic patients: a randomized trial | Nobre 2008 | Low | High | Unsure | Low | Low | High |
| Use of stepwise lactate kinetics-oriented hemodynamic therapy could improve the clinical outcomes of patients with sepsis-associated hyperlactatemia | Zhou 2017 | Low | High | Low | Low | Low | Unsure |
| Using Body Composition Analysis for Improved Nutritional Intervention in Septic Patients: a Prospective Interventional Study | Hung 2023 | Unsure | Low | Low | Low | Low | Unsure |
| Utilization of NIRS Monitor to Compare the Regional Cerebral Oxygen Saturation Between Dexmedetomidine and Propofol Sedation in Mechanically Ventilated Critically ill Patients with Sepsis- A Prospective Randomized Control Trial. | Patidar 2025 | Low | Low | Low | Low | Low | High |
| Vasopressin in Septic Shock; Assessment of Sepsis Biomarkers: a Randomized, Controlled Trial | Barzegar 2017 | Unsure | High | Unsure | Low | Unsure | High |
| Vasopressin Versus Norepinephrine for the Management of Septic Shock in Cancer Patients: the VANCS II Randomized Clinical Trial | Hajjar 2019 | Low | Low | Low | Low | Low | Low |
| Very Early Use of Esmolol in Hyperkinetic Septic Shock Patients with Persistent Tachycardia: A Randomized Controlled Pilot Study | Lu 2025 | Low | High | Unsure | Low | Low | High |
| Very high volume hemofiltration with the Cascade system in septic shock patients | Quenot 2015 | Low | High | High | Low | Low | High |
| Vitamin A treatment for severe sepsis in humans; a prospective randomized double blind placebo-controlled clinical trial | Cherukuri 2019 | Low | Low | Unsure | Low | Low | High |
| Vitamin c therapy or routine care in septic shock (ViCTOR) trial: effect of intravenous vitamin C, thiamine, and hydrocortisone administration on inpatient mortality among patients with septic shock | Mohamed 2020 | Unsure | High | Low | High | Low | High |
